# Targeting METTL3 protein by proteolysis-targeting chimeras: A novel therapeutic approach for acute myeloid leukemia

**DOI:** 10.1016/j.gendis.2024.101452

**Published:** 2024-11-07

**Authors:** Rukiye Nar, Zhixing Wu, Yafang Li, Alexis Smith, Yutao Zhang, Jue Wang, Fang Yu, Sanhui Gao, Chunjie Yu, Zhiguang Huo, Guangrong Zheng, Zhijian Qian

**Affiliations:** a Department of Medicine, UF Health Cancer Center, University of Florida, Gainesville, FL 32610, USA; b Department of Medicine, and Department of Biochemistry and Molecular Biology, University of Florida, Gainesville, FL 32610, USA; c Department of Medicinal Chemistry, University of Florida, Gainesville, FL 32610, USA; d Department of Biostatistics, University of Florida, Gainesville, FL 32610, USA

**Keywords:** Acute myeloid leukemia, METTL3, PROTAC, Protein degradation, ZW27941

## Abstract

Despite numerous studies suggesting that RNA m^6^A transferase core complex including METTL3 and METTL14 play essential roles in both the initiation and maintenance of acute myeloid leukemia (AML), effective pharmacological targeting of these two proteins remains elusive. Here, we report the development and evaluation of a novel METTL3 degrader, ZW27941, designed to induce METTL3 degradation via the VHL-mediated proteasomal degradation pathway. ZW27941 exhibited potent and selective degradation of METTL3 and its binding partner METTL14, leading to significant anti-leukemic activity in AML cell lines. Furthermore, ZW27941 demonstrated synergistic or additive effects when combined with standard AML therapeutics, such as cytarabine and venetoclax. Our findings suggest that selective METTL3 degraders, exemplified by ZW27941, hold promise as a novel therapeutic approach for AML, particularly when used in combination with existing treatments to enhance efficacy and overcome resistance mechanisms.

## Introduction

Acute myeloid leukemia (AML) stands as the most prevalent leukemia in the adult population, with approximately 20,000 new cases diagnosed annually in the United States.^1^^,^^2^ This aggressive blood cancer is characterized by uncontrolled proliferation of malignant hematopoietic stem and progenitor cells.^3^ Current treatment modalities for AML rely predominantly on chemotherapy, employing agents such as cytarabine and anthracyclines.^4^^,^^5^ Despite advancements in genetic characterization and personalized therapies, relapsed and refractory AML remains a formidable challenge.^6^^,^^7^ Population-based studies suggest a 5-year overall survival of 31.7% for all AML patients, estimated to reach 50% for younger patients and as low as 10% for those ≥60 years old.^8^^,^^9^ This underscores the critical need for improved options in effective and targeted anti-cancer therapies without toxicities to normal tissues for individuals with AML.

One of the most important post-transcriptional modifications implicated in AML initiation and progression is mRNA m^6^A modification.^10^ This modification is dynamic and reversible, managed by proteins known as writers (m^6^A methyltransferases), erasers (demethylases), and readers (m^6^A-binding proteins). The writer complex serving as the primary source of m^6^A in the cell is the methyltransferase-like (METTL)-associating complex (MACOM) including METTL3 and METTL14, along with other cofactors. METTL3 acts as the key catalytic component within the m^6^A methyltransferase complex.^11^^,^^12^ METTL3 plays a critical role in a diversity of diseases, notably cancer, heart failure, viral infection, and type 2 diabetes.^13^, ^14^, ^15^ AML cells exhibit elevated METTL3 protein levels compared with normal hematopoietic progenitors, establishing its significance as an essential gene for cancer cell growth.^16^, ^17^, ^18^ METTL3 down-regulation in AML cells induces cell cycle arrest, inhibits cell growth, accelerates apoptosis, and promotes differentiation.^16^^,^^17^^,^^19^ Recent developments in selective METTL3 inhibitors, such as UZH2 and STM2457, indicate the potential of pharmacological potential of targeting this protein. Both compounds, exhibiting high binding affinity to METTL3, significantly inhibit growth and enhance differentiation and apoptosis in various human AML cell lines.^20^^,^^21^ Despite promising low nanomolar potency in enzymatic and binding assays, UZH2 or STM2457 requires considerably higher concentrations in cells (IC_50_ at μM level) to inhibit METTL3 and reduce overall m^6^A mRNA modification levels in cells. This discrepancy is consistent with their S-adenosylmethionine-competitive mechanism of action and the competition from abundant intracellular S-adenosylmethionine/S-adenosylhomocysteine,^21^^,^^22^ limiting their potential for clinical translation. Several studies suggest that cytoplasmic METTL3 functions as an m^6^A reader, influencing the translation of RNA molecules marked with m^6^A modifications.^23^ Therefore, it is plausible that METTL3 also plays a role in regulating the oncogenesis of AML, partially independent of its m^6^A methyltransferase activity. Consequently, targeting the METTL3 enzymatic activity alone may only partially suppress its function in leukemogenesis. Alternative strategies, such as PROteolysis TArgeting Chimeras (PROTACs) targeting METTL3 may offer a solution to overcome the limitations of current small molecular inhibitors of METTL3 in the treatment of AML and other cancers.

PROTACs are bivalent small molecules containing a ligand that recognizes the target protein linked to an E3 ligase ligand. The interaction between the target protein and the E3 ligase, induced by the PROTAC, leads to polyubiquitination of the target protein and subsequent target protein degradation through the ubiquitin-proteasome system.^24^, ^25^, ^26^ PROTACs catalytically induce protein degradation in a sub-stoichiometric manner, so a much lower concentration is needed compared with small molecule inhibitors. The lower working concentration is important to provide a clinically meaningful therapeutic effect, and can also significantly reduce off-target, dose-limiting toxicities.^27^ Target selectivity can be achieved by converting a nonselective inhibitor to a PROTAC with the advantage of reducing systemic drug exposure.^28^ The multiple roles of METTL3 in driving and maintaining tumor growth make it a model candidate for PROTAC development with promising potential in future cancer therapies.

In this study, for more efficient targeting of METTL3 in AML, we developed and evaluated a Von Hippel-Lindau (VHL)-recruiting METTL3 PROTAC, ZW27941, that effectively degrades METTL3, thus temporarily eliminating all functions of the protein and demonstrating enhanced anti-leukemic activity.

## Materials and methods

### Chemical synthesis

**Scheme 1:**
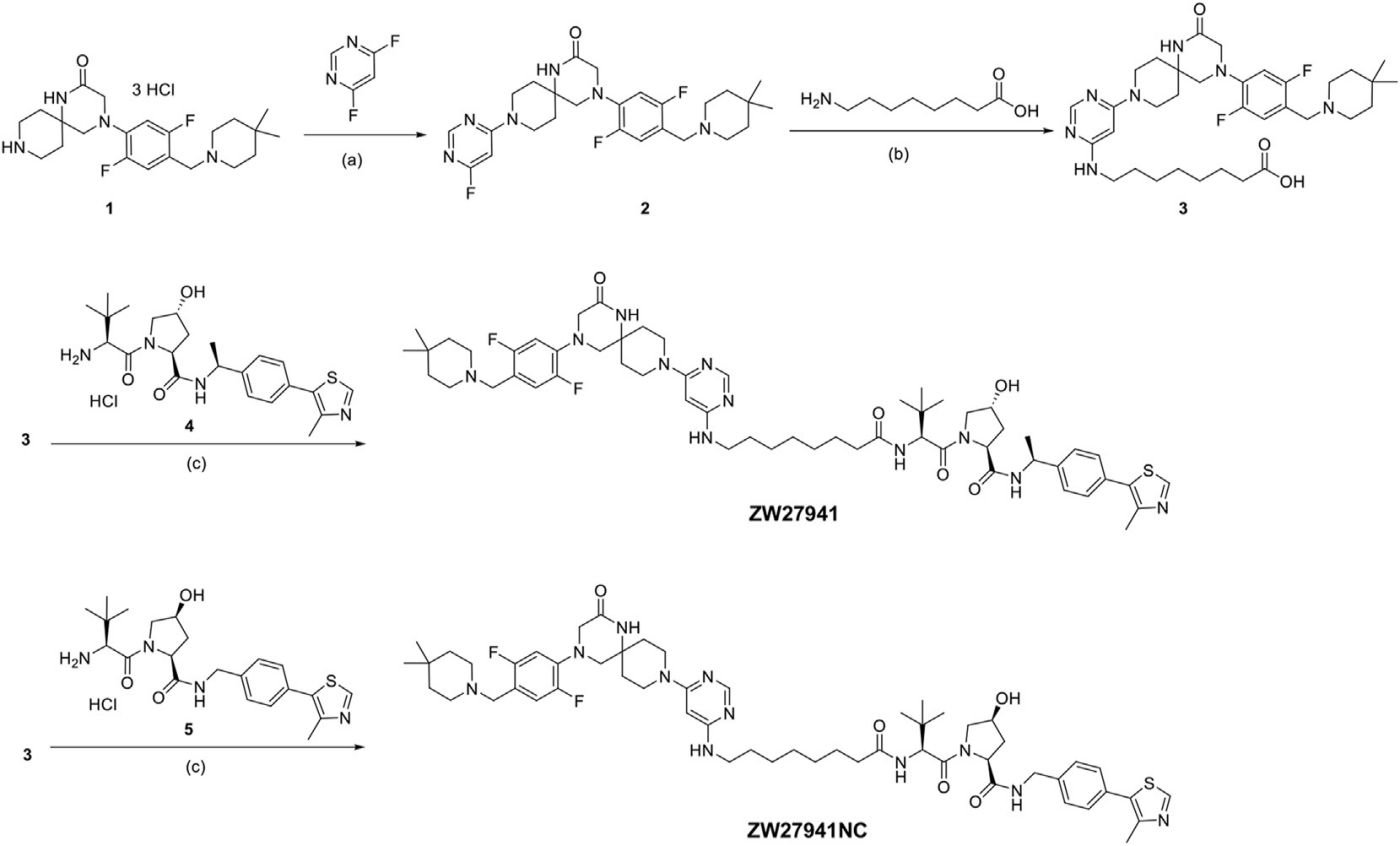
Synthesis of ZW27941 and ZW27941NC (negative control). Reagents and conditions: (a) DIPEA, isopropanol, 80 °C, microwave irradiation, 3 h; (b) DIPEA, isopropanol, H_2_O, 130 °C, microwave irradiation, 5 h; (c) HATU, DMF, DIPEA, room temperature, 2 h. DIPEA, diisopropylethylamine; DMF, dimethylformamide; HATU, hexafluorophosphate azabenzotriazole tetramethyl uronium.

The chemical structures, detailed synthetic procedures, and nuclear magnetic resonance spectra of METTL3 PROTACs are presented in supplementary data. The synthesis of ZW27941 and the negative control ZW27941NC is depicted in Scheme 1. First, a S_N_Ar reaction between the spiro ring starting material 1 (prepared following reported synthesis from Dolbois et al^20^) and 4,6-difluoropyrimdine afforded the fluoride intermediate 2 in quantitative yield. Intermediate 2 then underwent another S_N_Ar reaction with 8-aminooctanoic acid in isopropanol and H_2_O to give the acid intermediate 3 after trituration in methanol and dichloromethane. Finally, hexafluorophosphate azabenzotriazole tetramethyl uronium-mediated amide coupling of VHL ligand 4 or its corresponding negative ligand 5 with 3 in dimethylformamide yielded ZW27941 and ZW27941NC, respectively.

### Cell lines and cell culture

Human AML cell lines MOLM13, MV4.11, and NB4 and epithelial kidney HEK 293T cell lines were obtained from the American Type Culture Collection (ATCC, USA). AML cells were cultured in RPMI 1640 medium (Gibco, USA) supplemented with 10% (vol/vol) fetal bovine serum, and 1% penicillin-streptomycin (Sigma, Germany). HEK 293T cell lines were cultured in complete Dulbecco's modified Eagle medium with 10% fetal bovine serum and 1% penicillin-streptomycin (Sigma). All the cell lines were maintained in a humidified incubator at 37 °C and 5% CO_2_.

### Cell viability assay

AML cells were seeded at a density of 5000 cells in a 96-well plate and incubated with increasing concentrations of ZW27941, inhibitor, or vehicle (dimethylsulfoxide, DMSO). All experimental concentrations were replicated in triplicate. After treatment, cell viability was measured by cell counting kit-8 (CCK-8) according to instructions from the manufacturer (APExBIO, USA, Cat# K1018). The cell viability was determined by optical density values at 450 nm using a SPECTROstar Microplate Reader (BMG LABTECH, USA).

### Cell growth assay

In brief, 0.1 million cells per well were seeded in triplicate in 24-well plates and treated with varying concentrations of compounds. The medium, containing the compound, was refreshed every two days, and cell density was maintained below 1 × 10^6^/mL through periodic dilutions. Cell counts were assessed using an automated DS-11 cell counter (DeNovix, USA) every two days.

### Quantitative real-time PCR

Total RNA was extracted from cells using TRIzol Reagent (Life Technologies, USA) and reverse transcribed using iScript Reverse Transcription Supermix (BioRad, 1708841). Quantitative real-time PCR was performed on a QuantStudio 3 Real-Time PCR System (Applied Biosystems, USA) using SsoAdvanced Universal SYBR Green Supermix (BioRad, USA, 1725275). Transcript levels were normalized to glyceraldehyde-3-phosphate dehydrogenase (GAPDH) and fold changes were determined using the comparative Ct method. Student's *t*-test was performed for statistical significance. All quantitative real-time PCR primers are listed in Table S2.

### Lentivirus construction and packaging and lentiviral infection

Third-generation self-inactivating lentiviral transfer vectors, the plasmids encoding the different envelope proteins (pMD.G) and packaging vector pCMV-△R8.91 were co-transfected overnight into 60%–70 % confluent 293T cells (human Ad5-E1 transformed embryonic kidney cells containing expressing the Simian Virus 40 (SV40) T-antigen gene, obtained from ATCC, Manassas, Virginia, USA) using the polyethyleneimine (PEI) method.^29^^,^^30^

### Viral transduction

For viral transduction, MOLM13 cells were plated in a 24-well culture dish and spin infected with viral supernatants of the short hairpin RNA (shRNA) PLKO.1 METTL3 #1 and #2 or VHL #1 and #2 and Scramble Control supplemented with 8 μg/mL polybrene at 2000 rpm and 32 °C for 180 min using an Eppendorf Centrifuge 5810/5810R centrifuge. Then, the medium was removed and replaced with fresh medium overnight, and repeated spin infection was repeated for the second time. After 24 h, puromycin was added for infected cell selection and cultured for 3 days further before analysis.

### Western blot analysis

After specific treatment, total cellular proteins were extracted from cells using RIPA Buffer (Auragene, Changsha, China). Equal amounts of protein samples were resolved by 12% SDS-PAGE and then transferred onto polyvinylidene difluoride blotting membranes (Thermo Fisher, USA, LC2002). The following antibodies were used: ACTIN (Proteintech, Germany, 23660-1-AP), METTL3 (Abcam, UK, ab195352), METTL14 (Sigma–Aldrich, SAB5700855), METTL16 (Proteintech, 19924–1-AP), WTAP (Wilms tumor 1-associated protein; Proteintech, 10200-1-AP), and RBM15 (RNA binding motif protein 15; Proteintech, 10587-1-AP), VHL (Thermo Fisher, PA5-13488), GAPDH (Proteintech, 60004-1-IG), C-MYC (Invitrogen, USA, cat #13–2500). The immunoblots were quantified by densitometry using ImageJ Software, and the data were expressed as relative band intensities normalized to equal loading control and analyzed in GraphPad Prism 9.

### Cellular thermal shift assay

These experiments were performed according to published procedures.^31^^,^^32^

### Dot blot analysis of mRNA m^6^A methylation

To assess mRNA m^6^A methylation, dot-blot, and spectrophotometric assays were conducted following previously published protocols.^29^^,^^33^ For dot-blot assay membranes were scanned using Image Studio. Methylene blue staining served as a loading control, ensuring an equal amount of mRNA was used for dot-blot analysis. For RNA m^6^A quantification, EpiQuik m^6^A RNA methylation quantification kits (Epigentek, Farmingdale, NY, USA) were used as described by the manufacturer and determined by optical density values at 450 nm using a SPECTROstar microplate reader (BMG LABTECH, USA).

### Flow cytometric analysis

For the analysis of cellular apoptosis, transfected cells were stained using an antibody conjugate of APC annexin V (BD Biosciences, USA) with DAPI in the binding buffer, following the manufacturer's instructions. Incubation in the dark was carried out for 15 min at room temperature. In each measurement, a minimum of 10,000 cells were counted. Flow cytometric analysis was utilized to detect the stained cells. For cell cycle analysis, BrdU incorporation and DAPI staining were conducted following the manufacturer's guidelines (BD Biosciences, cat. 51-2090KZ). The data were acquired on a BD CytoFLEX flow cytometer (Becton Dickinson, USA) and analyzed using FlowJoTM 10 software (Tree Star Inc, Ashland, OR, USA).

### Colony-forming unit assay

Human cell lines were counted for cell viability and an equal number of live cells (1 × 10^3^ cells/mL) were plated in methylcellulose medium (M3134; StemCell Technologies, Inc., Vancouver, CA, USA), with different concentrations of ZW27941 or DMSO in 24-well culture plates for 7 days. The colony number was counted 7 days after plating under a reversed microscope. Aside from the colony count, the cell number was also counted to determine the colony size. Three independent experiments were carried out.

### RNA sequencing

RNA sequencing analysis was carried out to determine the differential gene expression profiles of MOLM13 cells treated with METTL3-targeting PROTAC. MOLM13 cells were cultured and treated with DMSO or ZW27941 (1 μM) for 24 h. Total RNAs were isolated using the Trizol reagent. RNA quality and quantity were accessed using a DeNovix DS-11 FX + spectrophotometer (DeNovix Inc., Wilmington, DE, USA). Isolated RNA was then subjected to quality control and quantification using an Agilent TapeStation Bioanalyzer. Total RNAs were used for RNA sequencing. The library constructions and sequencing were done with 50M raw reads/sample using the Illumina NovaSeq 6000 S4 2 × 150 platform at the Interdisciplinary Center for Biotechnology Research, University of Florida. The datasets related to ZW27941 used in this study can be accessed from the Gene Expression Omnibus (GEO) database with accession number GSE273361.

### Statistical analysis

In general, the results are expressed as mean ± standard deviation from triplicate experiments. The student's paired *t*-test was utilized to evaluate differences between groups, with *P* values < 0.05 considered statistically significant.

## Results

### Design, synthesis, and evaluation of VHL-based METTL3 PROTACs

**Figure 1:**
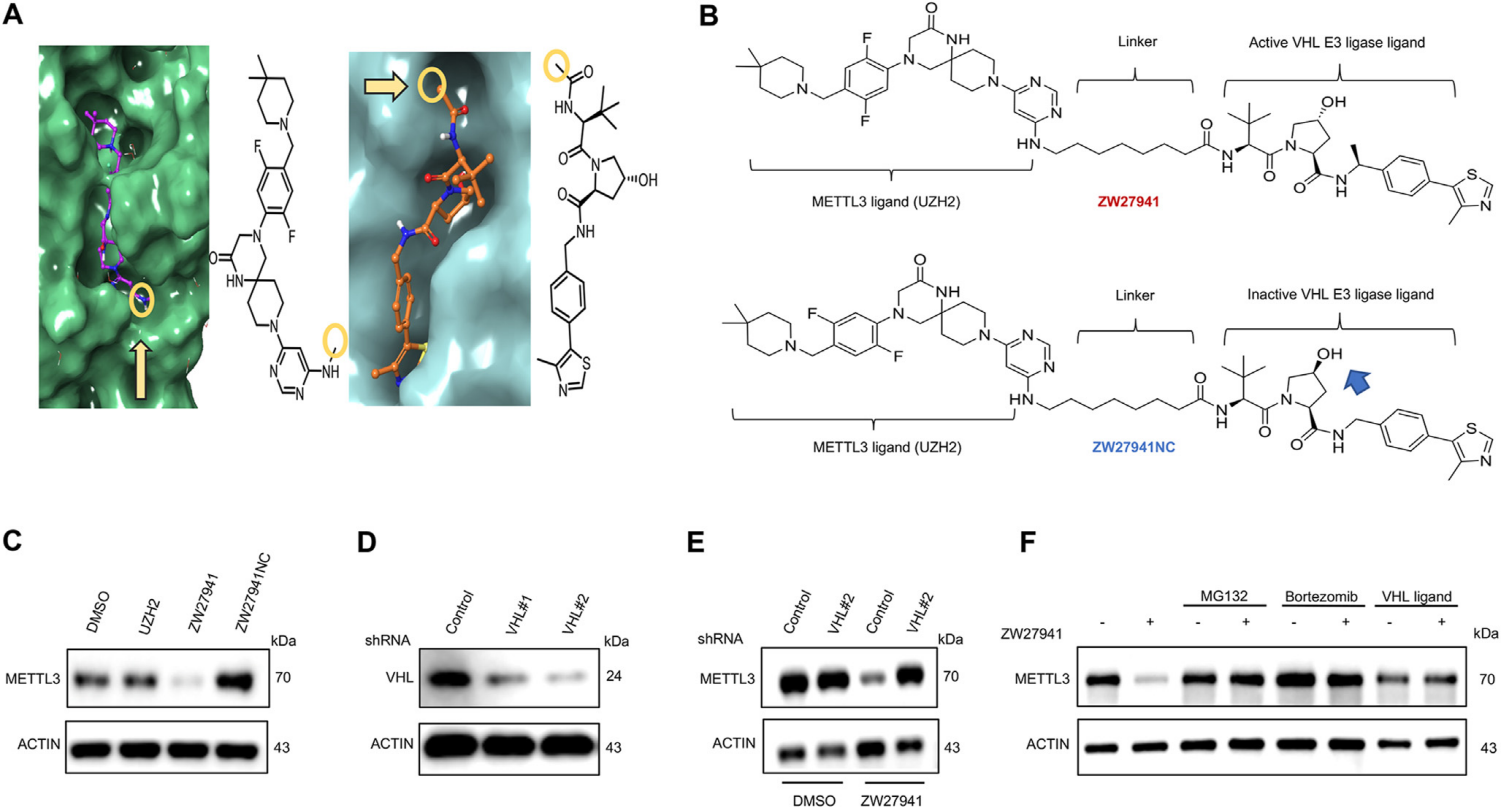
Structure-activity relationship and proteasomal degradation of METTL3 via VHL. **(A)** Selection of linkage points (yellow circle) on targeted protein ligands. The left is the crystal structure of UZH2-bound METTL3 (PDB ID 7O2F) and the right is the crystal structure of a VHL ligand bound to the VCB E3 ligase complex (PDB ID 4W9H). **(B)** The chemical structure of ZW27941 and ZW27941NC. **(C)** Western blot analysis of METTL3 in MV4.11 cells. The cells were treated with 1 μM of UZH2, ZW27941, and ZW27941NC for 24 h before being assayed. **(D)** MOLM13 cells were transfected with nontargeting shRNA control (SC) or VHL shRNA (shVHL), and VHL protein levels were analyzed by Western blot. **(E)** Transfected cells were treated with DMSO or 1 μM ZW27941 for 24 h, and METTL3 levels were analyzed by Western blot. **(F)** Pretreatment with VHL-L and proteasome inhibition blocks the METTL3 degradation by ZW27941. A representative of immunoblot analyses of METTL3 in HEK293T cells after they were either left untreated or pretreated with the proteasome inhibitor MG132 (1 μM) and bortezomib (0.1 μM) and VHL ligand (10 μM) for 1 h, and then treated with or without ZW27941 (5 μM) for 24 h.

To design a PROTAC degrader for METTL3, we selected UZH2 as the small molecule ligand scaffold. UZH2 exhibited single-digit nanomolar IC_50_ (5 nM) in the TR-FRET assay, high cell permeability (12 × 10^−6^ cm/s), and favorable ligand efficiency (LE) and ligand-lipophilicity efficiency (0.3 and 5.3, respectively), as well as acceptable metabolic stability (*t*_1/2_ = 24 min, rat liver microsomes).^20^ The linkage points are crucial for understanding the interaction between the protein ligands and their respective targets, providing valuable insights into the mechanisms underlying protein–protein interactions and ligand binding specificity. Figure 1A illustrates the selection of linkage points on targeted protein ligands. We systematically designed and synthesized a range of METTL3 PROTACs aimed at directing METTL3 to VHL for ubiquitination and subsequent degradation. These PROTACs featured alkane linkers with linker lengths ranging from 5 to 12 carbon atoms. The optimal linker length was determined through a comparative analysis of their potencies in inducing METTL3 degradation using different concentrations (1 and 10 μM) of 28 degraders in MOLM13 cells for 24 h (Fig. S1). The most significant METTL3 degradation was observed with PROTAC ZW27941, demonstrating a 76% reduction in METTL3 protein levels at 1 μM as quantified by Western blot (Table S1).

### ZW27941 initiates proteasomal degradation of METTL3 via VHL

To confirm that ZW27941 degrades METTL3 via VHL, the dependence of PROTAC activity on VHL binding was tested using a negative control compound, ZW27941NC, which was designed with an inversion of the stereocenters on the hydroxyproline moiety of the VHL ligand, resulting in the loss of affinity to VHL (Fig. 1B). This compound did not degrade METTL3 at a concentration of 1 μM (Fig. 1C). To further confirm the ZW27941-induced degradation of METTL3 was through the VHL ubiquitin E3 ligase, MOLM13 cells stably expressing scramble or shRNAs against VHL were generated and the knockdown efficiency of VHL shRNAs was confirmed by western blotting (Fig. 1D). We investigated whether reduced VHL expression would impact drug sensitivity. shVHL#2 exhibited more efficient inhibition of VHL expression, although VHL knockdown did not affect METTL3 levels (Fig. 1D, E). Upon testing ZW27941 at 1 μM in this cell line, while ZW27941 effectively degraded METTL3 in shControl cells, METTL3 levels remained unaffected by ZW27941 in shVHL#2 cells, resulting in decreased potency of ZW27941 (Fig. 1E). These data suggest that VHL is essential for ZW27941 to enhance its anti-cancer effects in AML cell lines by facilitating the protein turnover of METTL3.

To further elucidate the mechanism underlying the action of ZW27941, HEK293T cells were pre-treated with the proteasomal inhibitors MG132 (1 μM) or Bortezomib (0.1 μM) for 2 h before ZW27941 (5 μM) administration. Results showed that these proteasomal inhibitors effectively blocked the degradation of METTL3 induced by ZW27941. Furthermore, preincubation of HEK293T cells with a VHL ligand (10 μM) displayed a notable inhibition of ZW27941-induced METTL3 degradation (Fig. 1F). These findings collectively establish that ZW27941 induces METTL3 degradation through a VHL-mediated proteasomal degradation pathway.

### ZW27941 reduces cell viability and degrades METTL3 in a dose-dependent and time-dependent manner

**Figure 2:**
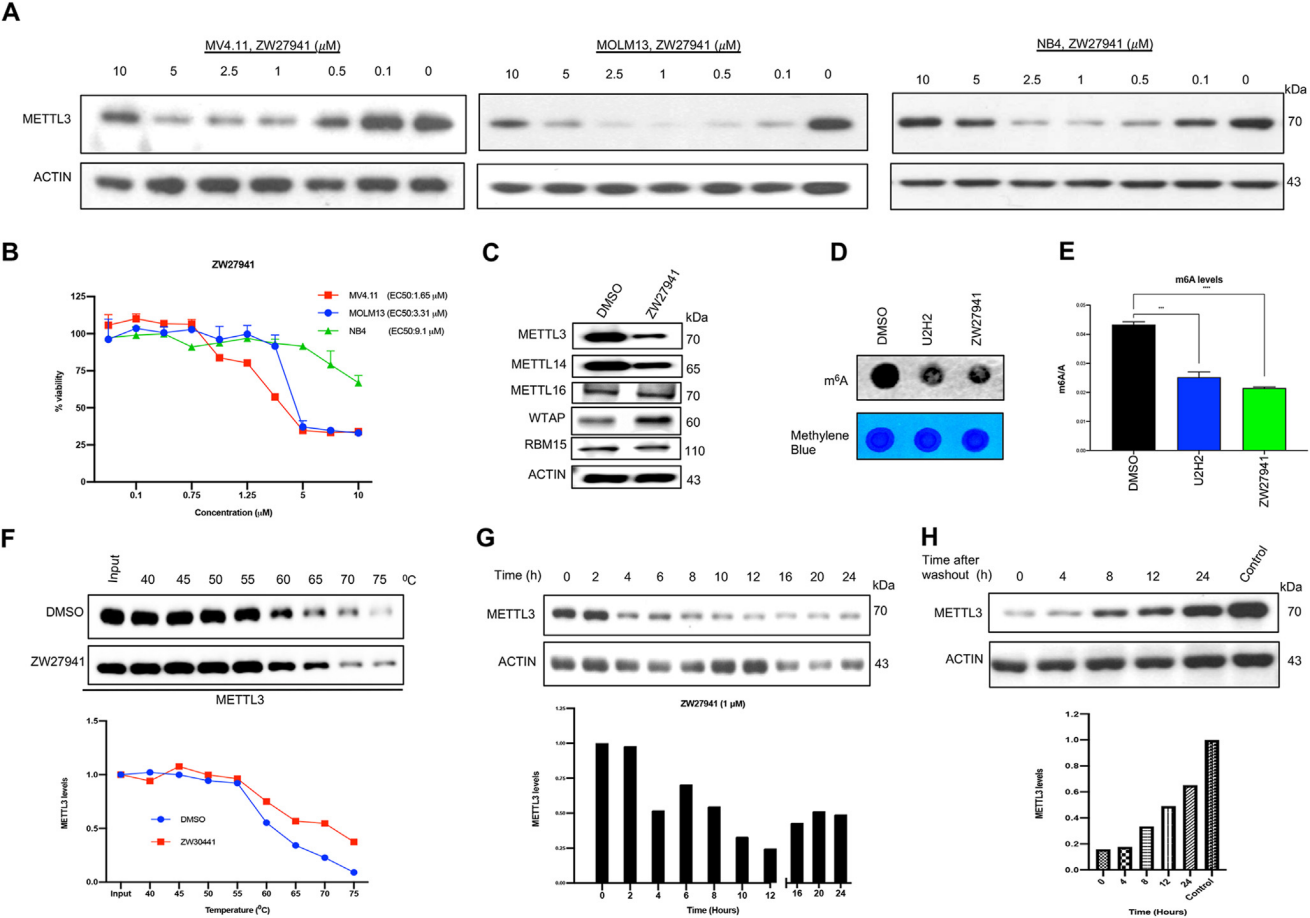
ZW27941 reduces cell viability and degrades METTL3 in a dose- and time-dependent manner. **(A)** The levels of the METTL3 protein in MV4.11, MOLM13, and NB4 cells treated with ZW27941 for 24 h detected by Western blot. METTL3 band intensity was normalized against ACTIN in each sample and DC_50_ (the drug concentration causing 50 % protein degradation) concentration was calculated. **(B)** Viability of MOLM13 and MV4.11 cells after they were treated with increasing concentrations of ZW27941 for 72 h. EC_50_ values are the average of three independent experiments. EC50 values were calculated based on the percentage of viable cells, normalized to control, as determined by CCK8 assay. **(C)** Western blot analysis of METTL3, METTL14, METTL16, WTAP, and RBM15 in NB4 cells after treated with 1 μM ZW27941 for 24 h. **(D)** The effect of UZH2 or ZW27941 (1 μM) treatment on global mRNA m^6^A levels in MOLM13 cells detected by dot-blot assay. **(E)** Quantification of m^6^A levels on polyA mRNA following 24 h treatment of MOLM13 cells with UZH2 or ZW27941 (1 μM). Data shown were mean ± standard deviation of triplicate experiments; ∗∗∗*P* < 0.001, ∗∗∗∗*P* < 0.0001. **(F)** Cellular thermal shift assay (CETSA) was conducted with MOLM13 cell lysate to monitor cellular target engagement. The cell lysate was incubated with either vehicle (DMSO) or ZW27941 (100 μM) for 30 min before melting at the indicated temperatures. The assay was performed in duplicate and western blots of METTL3 are shown. **(G)** Immunoblot analysis of METTL3 expression in MV4.11 cells after they were treated with 1 μM ZW27941 for various durations as indicated. Densitometric analysis of METTL3 expression is presented on the bottom panel as the mean of two independent experiments. **(H)** Analysis of METTL3 expression by immunoblot in MV4.11 cells treated with ZW27941 for 12 h followed by drug withdrawal and then culturing without ZW27941 for 0–24 h.

Our findings highlight the dose-dependent reduction of METTL3 protein by ZW27941 within 24 h, with a half-maximal degradation (DC_50_) of 0.70 μM in MV4.11, 0.17 μM in MOLM13, and 0.13 μM in NB4 cell lines (Fig. 2A). The “hook effect” is a distinct concentration-dependent activity pattern observed in PROTAC degraders. At high concentrations, PROTAC molecules can saturate binding sites on either the protein of interest or the E3 ligase, impeding the formation of the essential ternary complex and leading to the creation of binary complexes. This saturation at higher concentrations results in decreased activity, offering protection from induced degradation.^34^^,^^35^ Notably, our investigation confirms the occurrence of this phenomenon, prominently observed at 10 μM of ZW27941 (Fig. 2A).

We evaluated the cytotoxic activity of ZW27941 in AML cell lines by treating them with increasing doses of the compound for 72 h. AML cell viability was reduced in a dose-dependent manner, as shown by the CCK8 assay (Fig. 2B). Half-maximal effective concentration (EC_50_) of ZW27941 was calculated after normalization to the DMSO control. The degradation efficiency of a PROTAC is predominantly determined by its capacity to form a stable and cooperative ternary complex with its targeted protein(s) and E3 ligase.^36^ Treatment with 1 μM ZW27941 for 24 h in the NB4 cell line resulted in potent degradation of METTL3 and METTL14, key components of the m^6^A methyltransferase complex. Notably, ZW27941 exhibited no degradation on METTL16, WTAP, and RBM15, other proteins within the complex (Fig. 2C). To assess the impact of METTL3 degradation on global mRNA m^6^A methylation, MOLM13 cells were treated with ZW27941 and UZH2 at a concentration of 1 μM for 24 h. The outcomes revealed that both METTL3 inhibition and degradation led to a reduction in global RNA m^6^A levels (Fig. 2D, E).

To unravel the mechanism underlying the METTL3 degradation induced by ZW27941, we conducted measurements of the METTL3–ZW27941–VHL target engagement study *in vitro* using cellular thermal shift assay. This was achieved by measuring compound-induced alterations in protein thermal stability. The thermal stability shifts in METTL3 were notably more pronounced at each melting temperature when cells were incubated with ZW27941 compared with DMSO (Fig. 2F).

Treating cells with a PROTAC allows for reversible control of protein levels, a phenomenon dependent on both time and concentration.^35^ Efficient degradation of METTL3 was noted 4 h after treatment (Fig. 2G). Following the ZW27941 washout, protein levels began to recover (Fig. 2H). However, the impact persisted for over 24 h, suggesting that the observed degradation is not only rapid but also long-lasting.

### METTL3 degradation inhibits cell growth and colony formation in AML

METTL3 has been previously reported to sustain leukemia cells in a proliferating and undifferentiated state.^18^^,^^19^^,^^21^ After confirming the potency and selectivity of our lead METTL3 degrader, we examined its effects on AML cell lines. Initially, to validate METTL3 as a critical target for AML cell viability, we generated METTL3 knockdown MOLM13 cell lines (shMETTL3 #1 and shMETTL3 #2) using a shRNA vector system. We observed a correlation between METTL3 knockdown and the inhibition of AML cell growth along with the induction of apoptosis (Fig. S2). In summary, our findings indicate that METTL3 knockdown promotes apoptosis and inhibits the proliferation of the AML cell line, confirming the dependency of METTL3 in AML.

**Figure 3:**
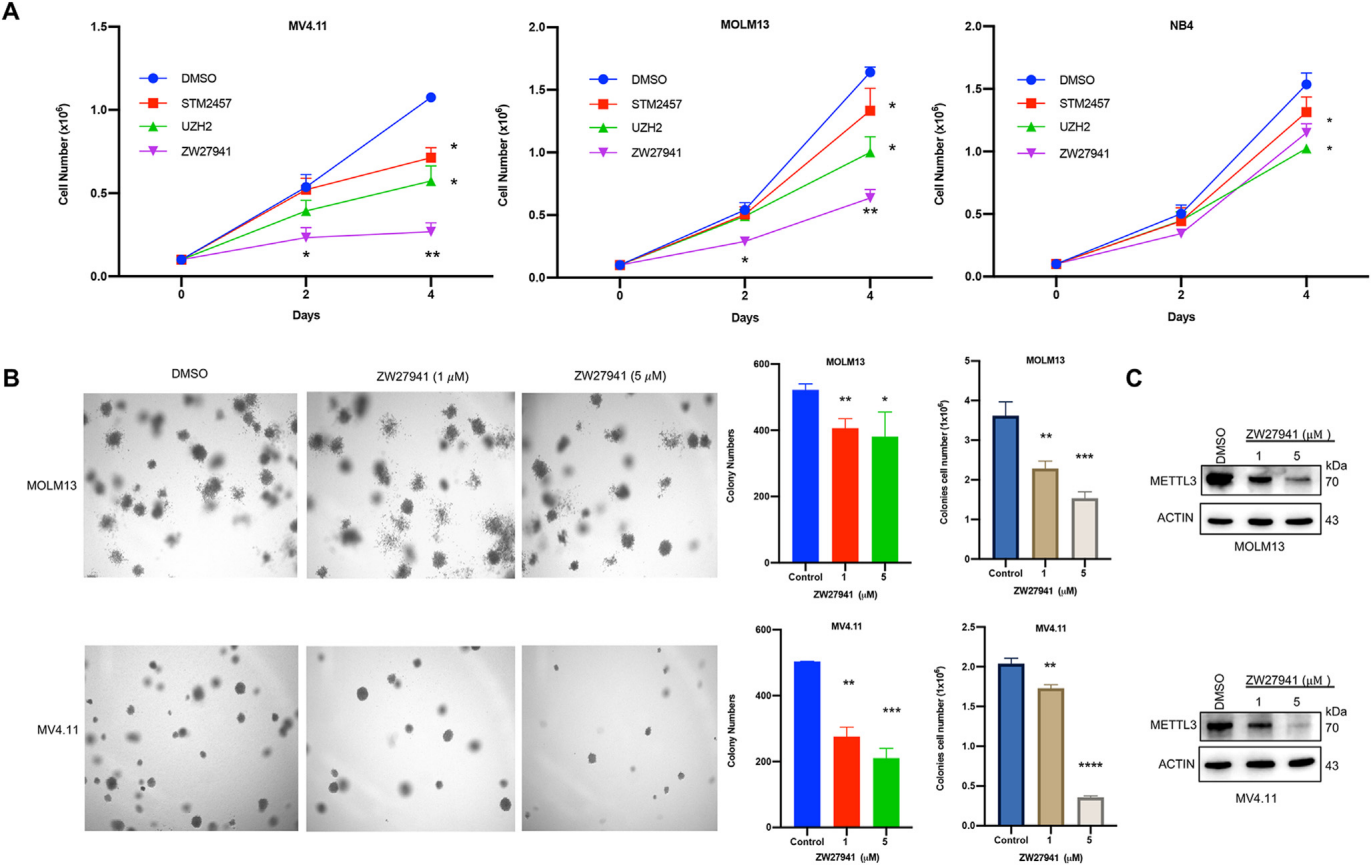
METTL3 inhibition affects the proliferation and survival of human acute myeloid leukemia cell lines. **(A)** Cell proliferation of leukemia cell lines treated with DMSO, STM2457, UZH2, or ZW27941 (5 μM) determined by trypan blue exclusion. **(B)** Colony formation assays used the indicated cell lines to expose to DMSO or ZW27941 at the indicated concentrations. Representative images are shown in the left panel, and the quantification of three independent experiments is shown in the right panel as bar graphs. Data shown were mean ± standard deviation of triplicate experiments; ∗*P* < 0.05, ∗∗*P* < 0.01, ∗∗∗*P* < 0.001, ∗∗∗∗*P* < 0.0001. **(C)** Analysis of METTL3 protein levels by immunoblot in MOLM13 and MV4.11 cells plated in methylcellulose medium and treated with DMSO, 1 μM ZW27941, and 5 μM ZW27941 for 7 days parallel to colony formation assay.

Furthermore, following a 4-day treatment with ZW27941, there was a significant decrease in the number of MOLM13, MV4.11, and NB4 cells in a time-dependent manner compared with DMSO and cells treated with METTL3 inhibitors STM2457 or UZH2 (Fig. 3A). To further assess the significance of degrader activity in inhibiting colony-forming ability, clonogenic growth assays were performed on various AML cell lines treated with DMSO and increasing doses of ZW27941. The number of colonies exhibited a significant decrease in a dosage-dependent manner for both MOLM13 and MV4.11 AML cell lines upon treatment with ZW27941 (Fig. 3B). To analyze the protein expression profile parallel to the colony-forming unit assay, we checked METTL3 protein levels by Western blot (Fig. 3C). The result shows sustained degradation of METTL3 in MOLM13 and MV4.11 AML cell lines treated with ZW27941 compared with DMSO control, validating the prolonged efficacy of the PROTAC treatment observed in the colony-forming unit assays. In conclusion, these data demonstrate that ZW27941 exhibits selectivity toward METTL3 as a protein target and possesses more potent anti-leukemic activity compared with METTL3 inhibitors.

### METTL3 degradation inhibits cell cycle progression and induces apoptosis

**Figure 4:**
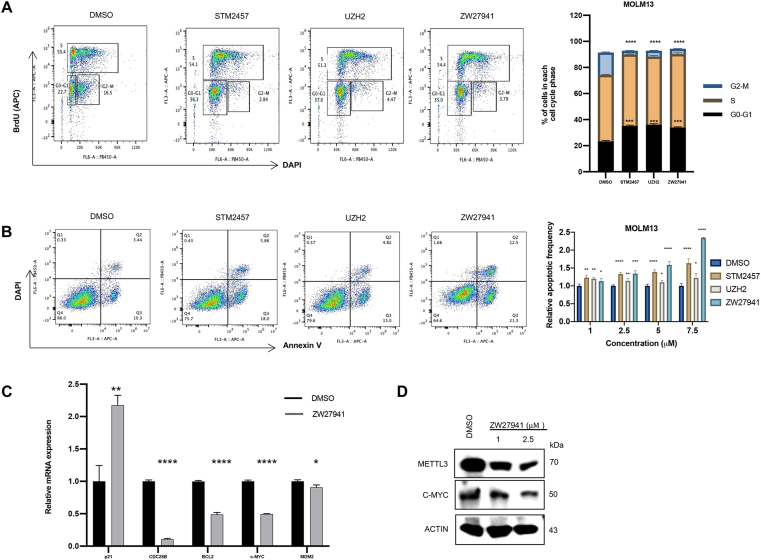
ZW27941 is highly potent in cell cycle progression and inducing apoptosis. **(A)** Flow cytometric analysis of the cell cycle of MOLM13 cells treated with DMSO, STM2457, UZH2, or ZW27941 (1 μM) for 24 h. The gating strategy is shown in the left upper panel. Cells were stained with BrdU (to determine the S phase) and DAPI (to determine the DNA content). **(B)** Flow cytometric analysis of the apoptosis of MOLM13 cells *in vitro*. The gating strategy is shown in the left panel. Cells were stained with annexin V and DAPI. AnnexinV ^+^ DAPI^−^ and AnnexinV^+^ DAPI^+^cells represent early and late apoptotic cells, respectively. All data are representative of two to three independent experiments. **(C)** Real-time PCR analysis of MOLM13 cells treated with ZW27941 (1 μM) assessed for relative mRNA expression of several genes. **(D)** Western blot analysis showed that the c-Myc protein levels were down-regulated in MOLM13 cells after treatment with different concentrations of ZW27941. Data shown were mean ± standard deviation of triplicate experiments; ∗*P* < 0.05, ∗∗*P* < 0.01, ∗∗∗*P* < 0.001, ∗∗∗∗*P* < 0.0001.

We next investigated whether ZW27941 and METTL3 inhibitors could regulate the cell cycle of AML cell lines. Cell cycle analysis using propidium iodide staining revealed an increased ratio of G0/G1 phase cells and a decreased ratio of G2/M phase cells in the treatment groups versus the DMSO-treated control group (Fig. 4A). METTL3 contributes to the regulation of cell cycle progression. It is well-documented that METTL3 influences the expression of key genes involved in cell cycle checkpoints including CDC25B.^37^, ^38^, ^39^ The results confirmed that the CDC25B mRNA expression was markedly decreased after ZW27941 treatment (Fig. 4C). This suggests that METTL3 inhibition, whether through degradation or enzymatic inhibition, can interfere with AML cell proliferation by inhibiting the cell cycle at multiple points. Additionally, AML cell apoptosis was observed after ZW27941 treatment. Annexin V staining analysis demonstrated that ZW27941 induced AML cell apoptosis (Fig. 4B). Overall, these results indicate that ZW27941 could block cell cycle progression and induce apoptosis in AML cell lines.

It has been reported that METTL3 can promote translation independently of its methyltransferase activity.^23^ Additionally, m^6^A has been shown to enhance the translation of c-MYC, BCL2, and PTEN mRNAs in human myeloid leukemia MOLM13 cells.^19^ Depletion of METTL3 and METTL14 in the K562 cellular model induces numerous alterations in the modulation of the p53 signaling cascade, characterized by the elevation of p53, up-regulation of cyclin-dependent kinase inhibitor 1A (CDKN1A/p21), and the suppression of MDM2 expression.^40^ Consistently, quantitative real-time PCR analysis showed that the transcriptional target of CDKN1A/p21 exhibited elevated levels, while BCL2, c-MYC, and MDM2 levels showed a significant decrease after treatment with the degrader ZW27941 (Fig. 4C, D). These findings confirm the regulatory role of METTL3 in the expression of these genes. Moreover, suppressing METTL3 via protein degradation produces noticeable effects on mRNA levels.

### Transcriptome profile of METTL3-degraded cells

**Figure 5:**
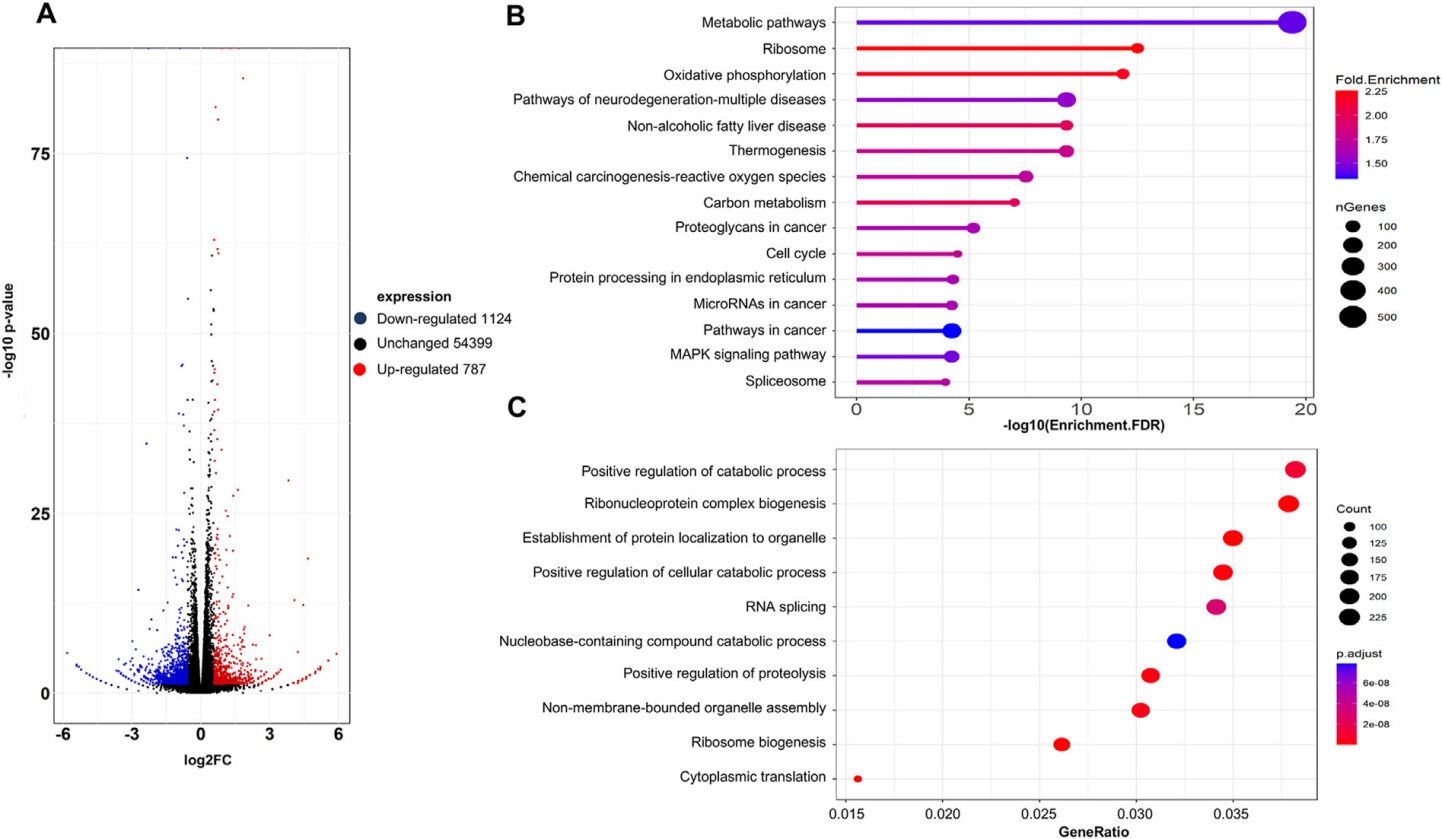
Differential expression analysis of MOLM13 cells after being treated with DMSO or ZW27941. **(A)** Volcano plot for MOLM13 cells treated with 1 μM ZW27941 versus DMSO samples after 24 h of treatment, showing significantly dysregulated genes (*P* < 0.05). **(B)** Gene ontology (GO) analysis of the differentially expressed genes post-treatment with 1 μM ZW27941 in MOLM13 cells. **(C)** KEGG pathway analysis of differentially expressed genes between DMSO and ZW27941 treated cells. Size is proportional to the number of differential expressed genes. Red to blue colors represent different adjusted *P* values.

To obtain a comprehensive understanding of the cellular changes in mRNA transcription following METTL3 degradation, we conducted RNA sequencing and performed data analysis to generate a transcriptome profile. MOLM13 cells treated with ZW27941 revealed 1911 differentially expressed genes, with 787 up-regulated and 1124 down-regulated (log_2_foldchange) > 1 and *P* < 0.05) (Fig. 5A; Table S4). Previous study indicates that METTL3 is involved in the regulation of translation through m^6^A methyltransferase-independent mechanism.^23^ Consistently, gene set enrichment analysis (GSEA) has shown that treatment of ZW27941 is associated with the reduced expression of genes responsible for protein translation initiation (Fig. S3). Furthermore, through GO and KEGG analysis, we discovered that the genes down-regulated after ZW27941 treatment were extensively involved in the metabolic process, cell cycle, and RNA regulation and processing pathways (Fig. 5B, C).

### Combination of ZW27941 with venetoclax or Ara-C exhibits cooperative antiproliferative activity in AML cell lines

**Figure 6:**
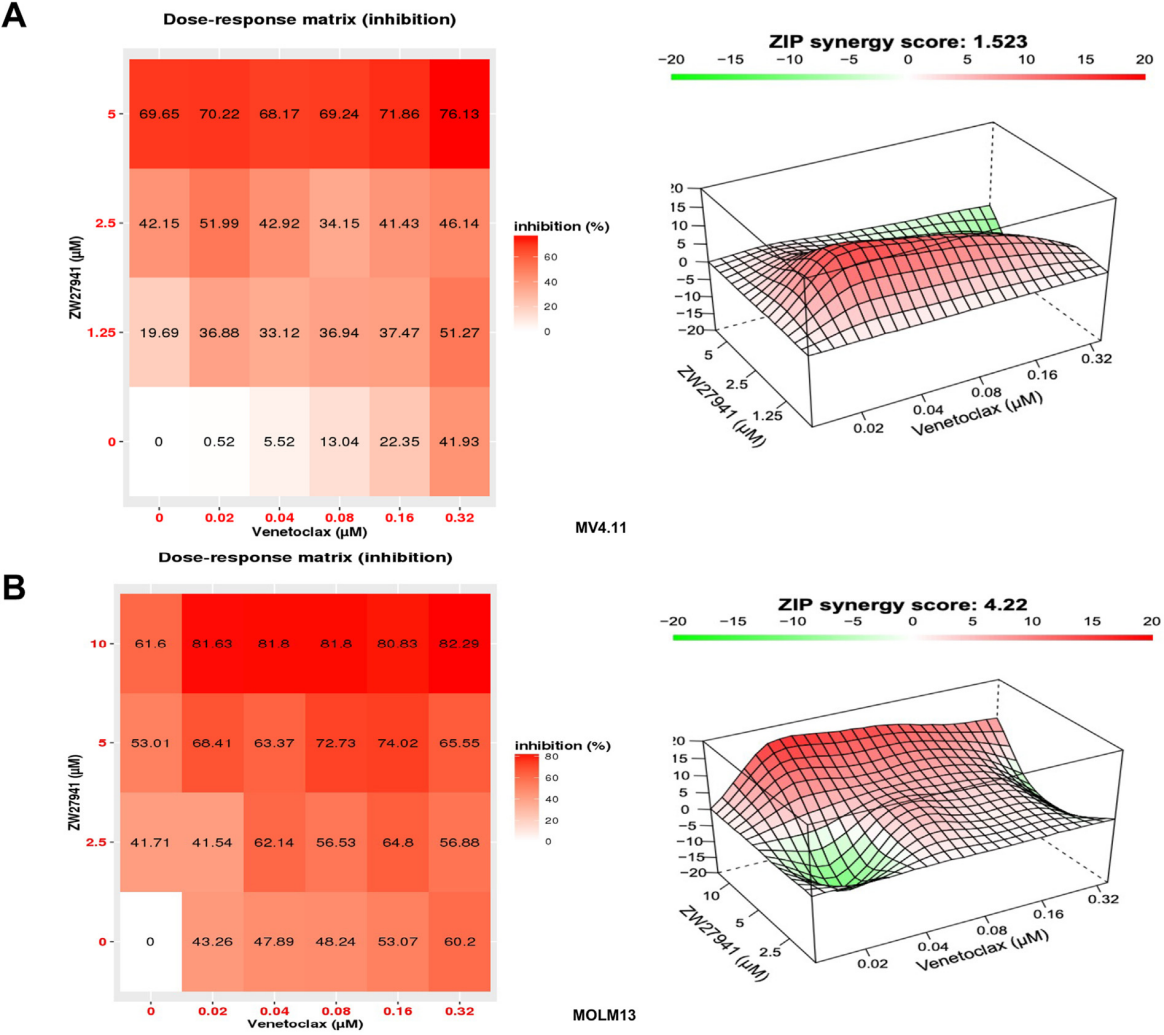
ZW27941 enhanced the efficacy of other therapeutic agents used for acute myeloid leukemia treatment. Drug dose matrix and synergy landscapes of human MV4.11 **(A)** and MOLM13 **(B)** cells treated with increasing concentrations of ZW27941, venetoclax, or their combinations for 72 h. Cell viability was evaluated using the CCK-8 assay. The presented results are representative of three independent experiments. The matrix illustrates the percentage of inhibition of treated cells versus vehicle controls. Drug combination landscapes were built using the bioconductor package “synergyfinder".

Targeted immunotherapy in AML faces challenges due to clonal heterogeneity, lack of surface protein expression, toxicity, and relapse. However, combination therapy for AML holds the potential to enhance therapeutic efficacy without increasing toxicity.^41^ We investigated the potential of METTL3 degradation to improve the effectiveness of agents such as venetoclax (BCL-2 inhibitor) and Ara-C (cytarabine or cytosine arabinoside) in combination with CCK8 assays. Our findings demonstrate that ZW27941 exhibits synergistic or additive anti-proliferative activity when combined with these agents (Fig. 6; Fig. S4). These results support the concept of METTL3 targeting as a promising therapeutic strategy for AML when used in combination with other agents.

## Discussion

As the most studied m^6^A methyltransferase, METTL3 has been reported to play roles in a variety of diseases, especially in cancer.^13^^,^^14^^,^^42^ Previous findings reveal the potential of pharmacological inhibition of METTL3 as an important therapeutic strategy for AML.^16^, ^17^, ^18^^,^^20^^,^^21^^,^^43^ Screening our library of VHL-directed METTL3 degraders against a panel of AML cell lines resulted in the identification of ZW27941. We demonstrate that ZW27941 is a potent, reversible, and efficacious METTL3 protein degrader. Interestingly, we also show that ZW27941 leads to the degradation of METTL14 protein, which is required for the maintenance of AML cells.^17^ ZW27941 degrades METTL3/METTL14 in a concentration- and time-dependent manner that requires proteasomal and ubiquitin ligase activities and VHL binding. We evaluated the anti-tumor effects of ZW27941 on AML cell lines, and the results indicated that ZW27941 exhibited good inhibitory effects on leukemia cell lines. In comparison to the prototype compound UZH2 used to construct ZW27941, the degrader ZW27941 exhibited enhanced potency to inhibit cancer cell proliferation/viability and colony formation, promote cell apoptosis, and cause cell cycle arrest at G0/G1.

Our study holds significance for AML drug development. Our results suggest that PROTAC degradation may be proven to be more effective than traditional binding inhibitors acting as enzymatic inhibitors. As reported by Ke et al, METTL3 acts as a positive regulator of mRNA translation, regardless of its methyltransferase activity.^44^ Additionally, non-enzymatic, potential oncogenic functions of METTL3 in metabolism and transcription are also inhibited by PROTACs, which remain unaffected by existing inhibitors. In the context of human myeloid leukemia, specifically the MOLM13 cell line, prior studies have emphasized the significance of METTL3 in regulating the expression of BCL2, c-Myc, and PTEN.^19^ Significantly, AML often exhibits elevated expression of both c-MYC and BCL-2. This overexpression is closely associated with chemotherapy resistance and is a predictor of poor clinical outcomes in patients with AML.^45^^,^^46^ These investigations have indicated that METTL3 influences genes associated with differentiation and cell survival in AML cells, operating through both direct and indirect mechanisms.^19^ Our findings demonstrate the down-regulation of c-Myc and BCL2 in MOLM13 cell line following METTL3 degradation. Importantly, our results confirm the dose-dependent decrease in c-Myc expression after treatment with ZW27941. Therefore, we conclude that METTL3 likely plays a role in sustaining the expression of c-Myc and BCL2 in specific acute leukemia cells, particularly implicating its involvement in c-Myc regulation.

Our study employed RNA sequencing to comprehensively elucidate the cellular changes in mRNA transcription following METTL3 degradation. Treatment of MOLM13 cells with ZW27941 resulted in significant differential expression of 1911 genes, with a notable proportion being down-regulated. Consistent with prior research, our findings suggest that METTL3 plays a role in regulating translation through an m^6^A-independent mechanism,^23^ as evidenced by reduced expression of genes involved in protein translation initiation upon ZW27941 treatment. Furthermore, GO and KEGG analyses revealed that genes down-regulated after ZW27941 treatment were enriched in metabolic process, cell cycle regulation, and RNA processing pathways.

Considering the likely necessity of combination therapies for AML treatment,^47^ we evaluated the efficacy of ZW27941 in combination with cytarabine (Ara-C) and BCL-2 inhibitor venetoclax. Our results demonstrate that ZW27941 can synergize with standard AML therapeutics, effectively inhibiting leukemia cell growth and enhancing overall efficacy.

While we were preparing our manuscript, a similar VHL-based PROTAC with a different linker structure, WD6305, for the METTL3-METTL14 complex was published.^48^ Similar to our findings, this study reported significant degradation of both METTL3 and METTL14 by WD6305, leading to reduced cell viability in AML models.^48^ Another study designed PROTACs against the m^6^A-RNA writer METTL3-METTL14 by using UZH2 and Cereblon (CRBN) ligand, achieving substantial METTL3−14 degradation in multiple AML cell lines.^49^ However, both studies also share limitations, such as the need for further optimization and validation in animal models to fully establish the therapeutic potential and safety of these degraders *in vivo*.

In summary, our study presents an exciting case for the use of selective METTL3 degraders in the context of leukemia. The inhibition profile of ZW27941 on various cell lines strongly indicates its efficacy in targeting METTL3, showcasing significant potency over UZH2. Beyond this specific comparison, our findings underscore the broader potential of a diverse library of METTL3 degraders in advancing lead discovery. Moreover, our study highlights the feasibility of rationally optimizing the potency and selectivity of molecular compounds. ZW27941 can cooperate with other anti-leukemic therapies and these combination therapies may provide greater therapeutic potential and overcome resistance mechanisms when treating AML.

## CRediT authorship contribution statement

**Rukiye Nar:** Investigation, Data curation, Methodology, Writing – original draft, Writing – review & editing. **Zhixing Wu:** Investigation, Writing – original draft, Writing – review & editing. **Yafang Li:** Data curation, Formal analysis, Methodology, Writing – original draft, Writing – review & editing. **Alexis Smith:** Formal analysis, Writing – review & editing. **Yutao Zhang:** Data curation, Formal analysis, Software, Writing – review & editing. **Jue Wang:** Formal analysis, Writing – review & editing. **Fang Yu:** Formal analysis, Methodology, Writing – review & editing. **Sanhui Gao:** Methodology, Writing – review & editing. **Chunjie Yu:** Methodology, Writing – review & editing. **Zhiguang Huo:** Conceptualization, Formal analysis, Supervision, Writing – review & editing. **Guangrong Zheng:** Conceptualization, Formal analysis, Writing – review & editing. **Zhijian Qian:** Conceptualization, Funding acquisition, Investigation, Project administration, Resources, Supervision, Writing – original draft, Writing – review & editing.

## Conflict of interests

R.N., Z.W., Y.L., A.S., G.Z., and Z.Q. are listed as inventors of a provisional patent application filed for METTL3/METTL14 PROTACs.

## Funding

This work was supported in part by the US National Institutes of Health (NIH) R01 grants (No. CA259576-01, CA266659 to Z.Q.) and UF (University of Florida) start-up grant (to Z.Q.). Z.Q. is a Leukemia & Lymphoma Society (LLS) Scholar.
