# Supplementary material for: Targeting METTL3 protein by proteolysis-targeting chimeras: A novel therapeutic approach for acute myeloid leukemia
Source: Genes Dis. 2024 Nov 7;12(4):101452. doi: 10.1016/j.gendis.2024.101452 (PMC12126954; doi:10.1016/j.gendis.2024.101452)
Supplement: Multimedia component 1 [file mmc1.pdf]

Supplementary material for

Targeting METTL3 protein by PROTAC: A Novel Therapeutic Approach for Acute Myeloid Leukemia

Rukiye Nar [ab1], Zhixing Wu [c1], Yafang Li [ab1], Alexis Smith[c], Yutao Zhang[d], Jue Wang[ab], Fang Yu[ab], Sanhui Gao[ab], Chunjie Yu[ab], Zhiguang Huo[d], Guangrong Zheng[c*] and Zhijian Qian[ab*]

[a] Department of Medicine, UF Health Cancer Center, University of Florida, Gainesville, FL 32610, USA

[b] Department of Medicine, and Department of Biochemistry and Molecular Biology, University of Florida, Gainesville, FL 32610, USA

[c] Department of Medicinal Chemistry, University of Florida, Gainesville, FL, 32610, USA

[d] Department of Biostatistics, University of Florida, Gainesville, FL, 32610, USA

[1] These authors contributed equally to the work and shared first authorship.

[*]Corresponding author.

E-mail addresses: zhijian.qian@medicine.ufl.edu (Z. Qian)

zhengg@cop.ufl.edu (G. Zheng)

Table of Contents

Chemistry 3
Experimental section 4
NMR spectra of degraders 27
SUPPLEMENTARY FIGURE S1 56
SUPPLEMENTARY TABLE S1 58
SUPPLEMENTARY TABLE S2 59
SUPPLEMENTARY TABLE S3 59
SUPPLEMENTARY FIGURE S2 60
SUPPLEMENTARY FIGURE S3 61

Chemistry

All reagents were purchased from commercial suppliers and used as received. DMF was obtained via a solvent purification system by filtering through two columns packed with activated alumina and 4 Å molecular sieve, respectively. Reactions run at elevated temperatures were carried out in the oil bath or under microwave irradiation using the Biotage® Initiator Microwave Synthesizer. Reaction progress was monitored by thin-layer chromatography (silica-coated glass plates) and visualized by 256nm and 365 nm UV light, and/or by LC-MS. Flash chromatography was performed using silica gel (230-400 mesh) as the stationary phase on the Biotage® Isolera purification system. Preparative thin-layer chromatography (Prep-TLC) was performed on silica gel 60 F_{254} 20 cm x 20 cm glass plates from Sigma-Aldrich. ^{1}H-NMR spectra were recorded in $CDCl_3$ at 600 MHz and ^{13}C-NMR spectra were recorded at 151 MHz using a Bruker (Billerica, MA) DRX Nuclear Magnetic Resonance (NMR) spectrometer. Chemical shifts are given in ppm using tetramethylsilane as an internal standard. Multiplicities of NMR signals are designated as singlet (s), doublet (d), doublet of doublets (dd), triplet (t), quartet (q), multiplet (m), and broad singlet (bs). All final compounds for biological testing were of ≥ 95.0% purity as analyzed by LC-MS, performed on an Advion AVANT LC system with the expression CMS using a Thermo Accucore™ Vanquish™ C18+ UHPLC Column (1.5 µM, 50 x 2.1 mm) at 40°C. Gradient elution was used for UHPLC with a mobile phase of acetonitrile and water containing 0.1% formic acid. High-resolution mass spectra (HRMS) were recorded on an Agilent 6230 Time-of-Flight (TOF) mass spectrometer.

Experimental section

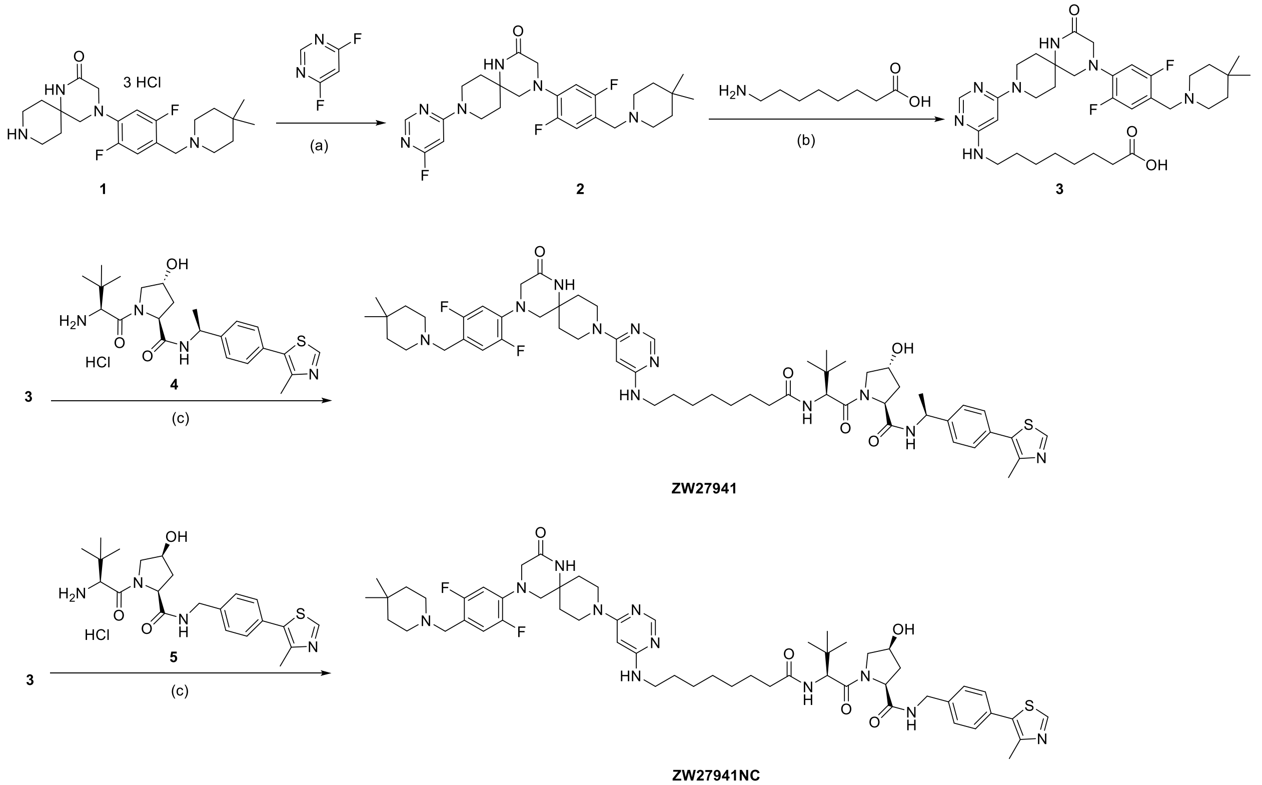

Scheme 1. Synthesis of ZW27941 and the negative control ZW27941NC[a]

[a]Reagents and conditions: (a) DIPEA, isopropanol, 80°C, microwave irradiation, 3 hrs; (b) DIPEA, isopropanol, H_2O, 130°C, microwave irradiation, 5 hrs; (c) HATU, DMF, DIPEA, rt, 2 hrs.

4-(4-((4,4-dimethylpiperidin-1-yl)methyl)-2,5-difluorophenyl)-9-(6-fluoropyrimidin-4-yl)-1,4,9-triazaspiro[5.5]undecan-2-one (2)

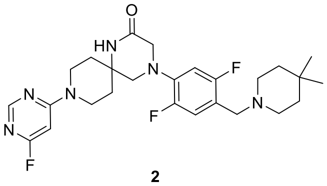

2

To a microwave reaction tube containing a suspension of 4-(4-((4,4-dimethylpiperidin-1-yl)methyl)-2,5-difluorophenyl)-1,4,9-triazaspiro[5.5]undecan-2-one hydrochloride [1] (100 mg, 0.2 mmol) and 4,6-difluoropyrimidine (92 mg, 0.8 mmol) in isopropanol (1 mL) was added DIPEA (260 mg, 2.0 mmol). The resulting mixture was purged with nitrogen and stirred at 80 °C under microwave irradiation for 3 hours. The reaction mixture was cooled to room temperature and partitioned between EtOAc and saturated aqueous NH_4Cl solution. The organic layer was washed with water and brine, dried over anhydrous Na_2SO_4, filtered, and concentrated. The residue was purified by flash column chromatography (MeOH in DCM 0-8%) to give the desired product (98 mg, 100% yield) as an off-white solid. ^{1}H-NMR (600 MHz, Chloroform-*d*): δ 8.33

(d, J = 2.7 Hz, 1H), 7.75 (s, 1H), 7.13 (dd, J = 12.8, 6.5 Hz, 1H), 6.57 (dd, J = 10.8, 7.1 Hz, 1H), 6.03 (s, 1H), 3.87 – 3.79 (m, 2H), 3.78 – 3.72 (m, 2H), 3.71 (s, 2H), 3.50 (s, 2H), 3.27 (s, 2H), 2.42 (s, 4H), 2.00 – 1.92 (m, 2H), 1.92 – 1.85 (m, 2H), 1.40 (t, J = 5.6 Hz, 4H), 0.90 (s, 6H) ppm. LRMS (ESI) *m/z* $[M+H]^+$: calculated, 503.3; found, 503.2.

8-((6-(4-(4-((4,4-dimethylpiperidin-1-yl)methyl)-2,5-difluorophenyl)-2-oxo-1,4,9-triazaspiro[5.5]undecan-9-yl)pyrimidin-4-yl)amino)octanoic acid (3)

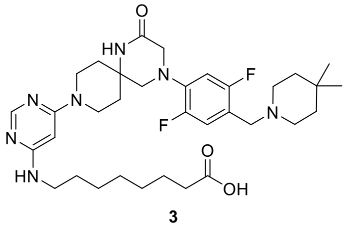

3

To a microwave tube containing a suspension of 4-(4-((4,4-dimethylpiperidin-1-yl)methyl)-2,5-difluorophenyl)-9-(6-fluoropyrimidin-4-yl)-1,4,9-triazaspiro[5.5]undecan-2-one (**2**, 20 mg, 0.04 mmol) in isopropanol (0.2 mL) was added 8-aminooctanoic acid (64 mg, 0.4 mmol, 10 eq.), DIPEA (52 mg, 0.4 mmol) and water (0.2 mL). The resulting mixture was stirred at 130 °C under microwave irradiation for 5 hours. The reaction mixture was diluted with MeOH (1 mL) and filtered through a short pad of anhydrous Na_2SO_4. The filtrate was concentrated, the residue was triturated with DCM/MeOH (2 mL, 10:1). The precipitated solid was filtered off and the filtrate with concentrated to give the desired product (quantitative yield) as a pale-yellow foam, which was used in the next step without further purification. LRMS (ESI) *m/z* $[M+H]^+$: calculated, 642.4; found, 642.4.

(2*S*,4*R*)-1-((*S*)-2-(8-((6-(4-(4-((4,4-dimethylpiperidin-1-yl)methyl)-2,5-difluorophenyl)-2-oxo-1,4,9-triazaspiro[5.5]undecan-9-yl)pyrimidin-4-yl)amino)octanamido)-3,3-dimethylbutanoyl)-4-hydroxy-*N*-((*S*)-1-(4-(4-methylthiazol-5-yl)phenyl)ethyl)pyrrolidine-2-carboxamide (ZW27941)

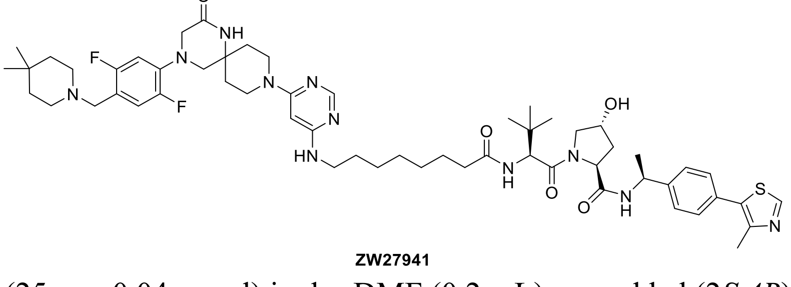

ZW27941

To a solution of **3** (25 mg, 0.04 mmol) in dry DMF (0.2 mL) was added (2*S*,4*R*)-1-((*S*)-2-amino-3,3-dimethylbutanoyl)-4-hydroxy-*N*-((*S*)-1-(4-(4-methylthiazol-5-yl)phenyl)ethyl)pyrrolidine-2-carboxamide HCl salt (44 mg, 0.08 mmol), HATU (30 mg, 0.08 mmol) and DIPEA (52 mg, 0.4 mmol). The resulting mixture was stirred at room temperature for 2 hours. The reaction mixture

was partitioned between EtOAc and water. The organic layer was washed with water, and brine, dried over anhydrous Na_2SO_4, filtered, and concentrated. The oil residue was purified by Prep-TLC (DCM/MeOH = 10 : 1) to give the desired product (5 mg, 12% yield) as a white foam. ^{1}H-NMR (600 MHz, Chloroform-*d*) δ 8.66 (s, 1H), 8.02 (s, 1H), 7.60 (d, *J* = 7.8 Hz, 1H), 7.54 (s, 1H), 7.41 – 7.31 (m, 4H), 7.12 (dd, *J* = 12.8, 6.6 Hz, 1H), 6.90 (s, 1H), 6.59 (dd, *J* = 10.8, 7.1 Hz, 1H), 5.43 (s, 1H), 5.31 – 5.28 (m, 1H), 5.13 – 5.05 (m, 1H), 4.63 (d, *J* = 9.1 Hz, 1H), 4.51 (t, *J* = 8.2 Hz, 1H), 4.48 – 4.43 (m, 1H), 4.08 (dt, *J* = 11.4, 1.8 Hz, 1H), 4.00 – 3.94 (m, 1H), 3.73 (d, *J* = 15.4 Hz, 1H), 3.70 (s, 2H), 3.60 (dd, *J* = 11.3, 3.6 Hz, 1H), 3.56 – 3.43 (m, 4H), 3.38 (d, *J* = 12.5 Hz, 1H), 3.20 (d, *J* = 12.5 Hz, 1H), 3.18 – 3.11 (m, 2H), 2.51 (s, 3H), 2.42 (s, 4H), 2.33 – 2.26 (m, 1H), 2.23 – 2.10 (m, 2H), 2.04 – 1.95 (m, 2H), 1.93 – 1.86 (m, 2H), 1.85 – 1.78 (m, 1H), 1.59 – 1.51 (m, 4H), 1.48 (d, *J* = 6.9 Hz, 3H), 1.42 – 1.37 (m, 4H), 1.36 – 1.18 (m, 5H), 1.02 (s, 9H), 0.90 (s, 6H) ppm. ^{13}C-NMR (151 MHz, $CDCl_3$) δ 173.65, 171.79, 169.63, 168.00, 163.09, 162.23, 157.99, 157.50, 156.38, 151.67, 150.22, 150.08, 148.36, 143.16, 137.43, 131.50, 130.72, 129.43, 126.36, 118.30, 105.63, 69.74, 58.65, 57.40, 56.88, 56.08, 54.48, 53.34, 52.83, 49.61, 48.65, 41.30, 40.45, 40.07, 38.33, 36.10, 35.93, 35.25, 35.01, 34.91, 29.59, 28.57, 28.48, 28.22, 26.46, 26.35, 25.17, 21.99, 16.00 ppm. HRMS (ESI) *m/z*: $[M+H]^+$ calculated for $C_{57}H_{79}F_2N_{11}O_5S$ 1068.5954; found, 1068.6003.

(2*S*,4*S*)-1-((*S*)-2-(8-((6-(4-(4-((4,4-dimethylpiperidin-1-yl)methyl)-2,5-difluorophenyl)-2-oxo-1,4,9-triazaspiro[5.5]undecan-9-yl)pyrimidin-4-yl)amino)octanamido)-3,3-dimethylbutanoyl)-4-hydroxy-N-(4-(4-methylthiazol-5-yl)benzyl)pyrrolidine-2-carboxamide (ZW27941NC)

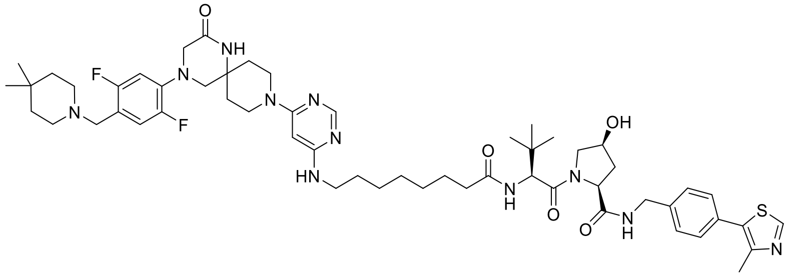

ZW27941NC

ZW27941NC was synthesized from intermediate **3** and (2*S*,4*S*)-1-((*S*)-2-amino-3,3-dimethylbutanoyl)-4-hydroxy-*N*-(4-(4-methylthiazol-5-yl)benzyl)pyrrolidine-2-carboxamide hydrochloride using the same procedure described for ZW27941. ^{1}H-NMR (600 MHz, Chloroform-*d*) δ 8.67 (s, 1H), 8.46 (s, 1H), 8.05 (s, 1H), 7.35 (s, 4H), 7.30 (s, 1H), 7.10 (dd, *J* = 12.8, 6.6 Hz, 1H), 6.56 (dd, *J* = 10.8, 7.1 Hz, 1H), 6.44 (d, *J* = 9.0 Hz, 1H), 5.40 (s, 1H), 5.36 (s, 1H), 4.67 (dd, *J* = 15.0, 7.0 Hz, 1H), 4.60 (dd, *J* = 7.8, 2.6 Hz, 1H), 4.57 (d, *J* = 9.1 Hz, 1H), 4.43

(t, *J* = 4.3 Hz, 1H), 4.27 (dd, *J* = 15.0, 4.9 Hz, 1H), 3.95 (dd, *J* = 11.1, 4.1 Hz, 1H), 3.90 (d, *J* = 11.1 Hz, 1H), 3.81 (td, *J* = 16.5, 15.5, 4.2 Hz, 2H), 3.71 – 3.62 (m, 2H), 3.50 – 3.39 (m, 4H), 3.29 (d, *J* = 12.5 Hz, 1H), 3.17 (dd, *J* = 15.5, 9.4 Hz, 3H), 2.50 (s, 3H), 2.40 (s, 4H), 2.22 – 2.14 (m, 5H), 1.99 – 1.92 (m, 2H), 1.84 – 1.75 (m, 3H), 1.62 – 1.54 (m, 4H), 1.38 (t, *J* = 5.6 Hz, 5H), 1.35 – 1.28 (m, 4H), 0.95 (s, 9H), 0.90 (s, 6H) ppm. ^{13}C NMR (151 MHz, $CDCl_3$) δ 172.98, 172.06, 167.90, 163.16, 162.36, 157.96, 157.62, 156.35, 151.73, 150.22, 148.43, 137.62, 137.19, 131.43, 130.97, 129.41, 128.04, 118.31, 105.69, 105.49, 71.15, 59.79, 58.55, 56.99, 55.96, 54.61, 53.39, 52.78, 49.71, 43.35, 41.24, 40.58, 40.37, 38.53, 35.84, 34.91, 29.63, 28.69, 28.58, 28.41, 28.28, 26.50, 26.32, 25.09, 16.02 ppm. HRMS (ESI) *m/z*: $[M+H]^+$ calculated for $C_{56}H_{77}F_2N_{11}O_5S$ 1054.5798; found, 1054.5851.

(2*S*,4*R*)-1-((*S*)-2-(5-((6-(4-(4-((4,4-dimethylpiperidin-1-yl)methyl)-2,5-difluorophenyl)-2-oxo-1,4,9-triazaspiro[5.5]undecan-9-yl)pyrimidin-4-yl)amino)pentanamido)-3,3-dimethylbutanoyl)-4-hydroxy-*N*-((*S*)-1-(4-(4-methylthiazol-5-yl)phenyl)ethyl)pyrrolidine-2-carboxamide (ZW27938)

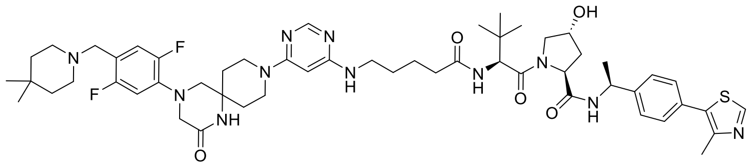

ZW27938

SnAr reaction between intermediate **2** and 5-aminovaleric acid (10 eq.) afforded the acid intermediate in quantitative yield and the subsequent amide coupling with (2*S*,4*R*)-1-((*S*)-2-amino-3,3-dimethylbutanoyl)-4-hydroxy-*N*-((*S*)-1-(4-(4-methylthiazol-5-yl)phenyl)ethyl)pyrrolidine-2-carboxamide HCl salt in the presence of HATU and DIPEA in DMF gave the titled degrader ZW27938 as a white foam. ^{1}H-NMR (600 MHz, Chloroform-*d*) *δ* 8.67 (s, 1H), 7.98 (s, 1H), 7.74 (d, *J* = 7.8 Hz, 1H), 7.53 – 7.49 (m, 1H), 7.41 – 7.35 (m, 4H), 7.13 (dd, *J* = 12.8, 6.5 Hz, 1H), 6.59 (dd, *J* = 10.8, 7.1 Hz, 1H), 5.49 (s, 1H), 5.44 (s, 1H), 5.15 – 5.07 (m, 1H), 4.62 (d, *J* = 9.1 Hz, 1H), 4.50 – 4.44 (m, 2H), 4.05 (d, *J* = 11.1 Hz, 1H), 3.95 (d, *J* = 13.7 Hz, 1H), 3.70 (s, 3H), 3.68 – 3.59 (m, 1H), 3.61 – 3.54 (m, 1H), 3.51 (s, 2H), 3.49 – 3.41 (m, 1H), 3.37 (d, *J* = 12.5 Hz, 1H), 3.24 (s, 1H), 3.20 (d, *J* = 12.5 Hz, 1H), 3.18 – 3.10 (m, 1H), 2.51 (s, 3H), 2.46 – 2.41 (m, 4H), 2.30 – 2.15 (m, 3H), 2.03 – 1.92 (m, 1H), 1.91 – 1.86 (m, 2H), 1.86 – 1.78 (m, 1H), 1.72 – 1.54 (m, 3H), 1.50 (d, *J* = 6.9 Hz, 3H), 1.40 (t, *J* = 5.6 Hz, 4H), 1.29 – 1.21 (m, 2H), 1.03 (s, 9H), 0.91 (s, 6H) ppm. LRMS (ESI) *m/z* $[M+H]^+$: calculated, 1026.6; found, 1026.4.

(2*S*,4*R*)-1-((*S*)-2-(6-((6-(4-(4-((4,4-dimethylpiperidin-1-yl)methyl)-2,5-difluorophenyl)-2-oxo-1,4,9-triazaspiro[5.5]undecan-9-yl)pyrimidin-4-yl)amino)hexanamido)-3,3-dimethylbutanoyl)-4-hydroxy-*N*-((*S*)-1-(4-(4-methylthiazol-5-yl)phenyl)ethyl)pyrrolidine-2-carboxamide (ZW27935)

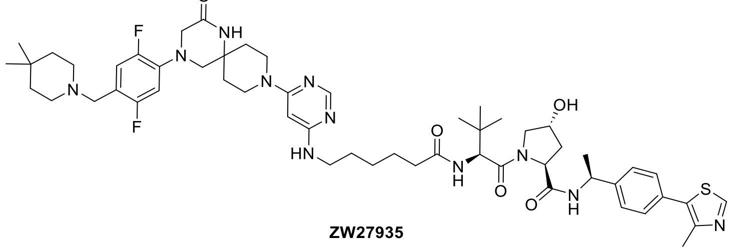

ZW27935

SnAr reaction between intermediate **2** and 6-aminohexanoic acid (10 eq.) afforded the acid intermediate in quantitative yield and the subsequent amide coupling with (2*S*,4*R*)-1-((*S*)-2-amino-3,3-dimethylbutanoyl)-4-hydroxy-*N*-((*S*)-1-(4-(4-methylthiazol-5-yl)phenyl)ethyl)pyrrolidine-2-carboxamide HCl salt in the presence of HATU and DIPEA in DMF gave the titled degrader ZW27935 as a white foam. ^{1}H-NMR (600 MHz, Chloroform-*d*) δ 8.66 (s, 1H), 7.92 (s, 1H), 7.82 (d, J = 7.7 Hz, 1H), 7.41 – 7.34 (m, 5H), 7.13 (dd, J = 12.9, 6.5 Hz, 1H), 6.87 – 6.84 (m, 1H), 6.59 (dd, J = 10.8, 7.1 Hz, 1H), 5.42 (s, 1H), 5.30 (s, 1H), 5.15 – 5.07 (m, 1H), 4.63 (d, J = 9.2 Hz, 1H), 4.52 (t, J = 8.2 Hz, 1H), 4.50 – 4.45 (m, 1H), 4.06 (d, J = 11.3 Hz, 1H), 3.97 – 3.90 (m, 1H), 3.70 (s, 3H), 3.61 (td, J = 14.9, 12.9, 4.8 Hz, 2H), 3.51 – 3.45 (m, 3H), 3.36 (d, J = 12.5 Hz, 1H), 3.20 (d, J = 12.5 Hz, 1H), 3.16 – 3.10 (m, 2H), 2.52 (s, 3H), 2.42 (s, 4H), 2.30 – 2.21 (m, 2H), 2.21 – 2.13 (m, 1H), 2.02 – 1.93 (m, 1H), 1.93 – 1.88 (m, 2H), 1.86 – 1.78 (m, 1H), 1.67 (dt, J = 14.5, 7.2 Hz, 1H), 1.63 – 1.53 (m, 3H), 1.50 (d, J = 7.0 Hz, 3H), 1.40 (t, J = 5.5 Hz, 4H), 1.38 – 1.31 (m, 2H), 1.04 (s, 9H), 0.91 (s, 6H) ppm. LRMS (ESI) *m/z* $[M+H]^+$: calculated, 1040.6; found, 1040.6.

(2*S*,4*R*)-1-((S)-2-(7-((6-(4-(4-((4,4-dimethylpiperidin-1-yl)methyl)-2,5-difluorophenyl)-2-oxo-1,4,9-triazaspiro[5.5]undecan-9-yl)pyrimidin-4-yl)amino)heptanamido)-3,3-dimethylbutanoyl)-4-hydroxy-*N*-((*S*)-1-(4-(4-methylthiazol-5-yl)phenyl)ethyl)pyrrolidine-2-carboxamide (ZW27940)

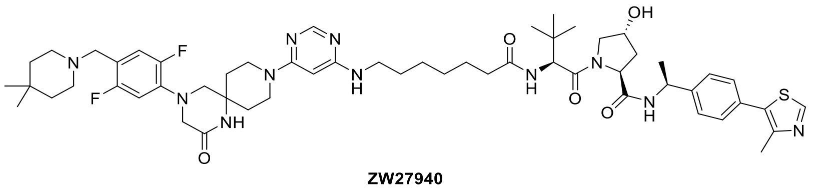

ZW27940

SnAr reaction between intermediate **2** and 7-aminoheptanoic acid (10 eq.) afforded the acid intermediate in quantitative yield and the subsequent amide coupling with (2*S*,4*R*)-1-((*S*)-2-amino-3,3-dimethylbutanoyl)-4-hydroxy-*N*-((*S*)-1-(4-(4-methylthiazol-5-

yl)phenyl)ethyl)pyrrolidine-2-carboxamide HCl salt in the presence of HATU and DIPEA in DMF gave the titled degrader ZW27940 as a white foam. ^{1}H NMR (600 MHz, Chloroform-*d*) δ 8.66 (s, 1H), 8.02 (s, 1H), 7.62 (d, *J* = 7.8 Hz, 1H), 7.40 – 7.34 (m, 5H), 7.14 (dd, *J* = 12.9, 6.6 Hz, 1H), 6.77 (s, 1H), 6.60 (dd, *J* = 10.8, 7.1 Hz, 1H), 5.46 (s, 1H), 5.28 (s, 1H), 5.12 – 5.06 (m, 1H), 4.65 – 4.54 (m, 2H), 4.46 (s, 1H), 4.08 (d, *J* = 11.2 Hz, 1H), 3.97 – 3.89 (m, 1H), 3.70 (s, 2H), 3.61 (dd, *J* = 11.2, 3.5 Hz, 1H), 3.55 (s, 2H), 3.48 (t, *J* = 9.4 Hz, 1H), 3.34 (d, *J* = 12.5 Hz, 1H), 3.24 (d, *J* = 12.5 Hz, 1H), 3.21 – 3.15 (m, 2H), 2.52 (s, 3H), 2.48 (s, 4H), 2.32 – 2.28 (m, 1H), 2.20 – 2.13 (m, 2H), 2.04 – 1.98 (m, 1H), 1.98 – 1.87 (m, 2H), 1.85 – 1.79 (m, 1H), 1.61 – 1.54 (m, 4H), 1.48 (d, *J* = 6.9 Hz, 4H), 1.44 – 1.40 (m, 5H), 1.37 – 1.28 (m, 5H), 1.03 (s, 9H), 0.92 (s, 6H) ppm. LRMS (ESI) *m/z* $[M+H]^+$: calculated, 1054.6; found, 1054.5.

(2*S*,4*R*)-1-((*S*)-2-(9-((6-(4-(4-((4,4-dimethylpiperidin-1-yl)methyl)-2,5-difluorophenyl)-2-oxo-1,4,9-triazaspiro[5.5]undecan-9-yl)pyrimidin-4-yl)amino)nonanamido)-3,3-dimethylbutanoyl)-4-hydroxy-*N*-((*S*)-1-(4-(4-methylthiazol-5-yl)phenyl)ethyl)pyrrolidine-2-carboxamide (ZW30405)

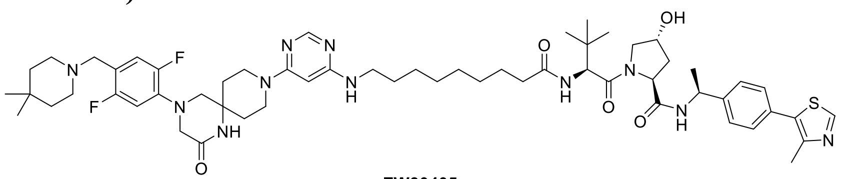

ZW30405

SnAr reaction between intermediate **2** and 9-aminononanoic acid (10 eq.) afforded the acid intermediate in quantitative yield and the subsequent amide coupling with (2*S*,4*R*)-1-((*S*)-2-amino-3,3-dimethylbutanoyl)-4-hydroxy-*N*-((*S*)-1-(4-(4-methylthiazol-5-yl)phenyl)ethyl)pyrrolidine-2-carboxamide HCl salt in the presence of HATU and DIPEA in DMF gave the titled degrader ZW30405 as a white foam. ^{1}H-NMR (600 MHz, Chloroform-*d*) δ 8.67 (s, 1H), 8.09 (s, 1H), 7.51 (d, *J* = 7.9 Hz, 1H), 7.42 – 7.35 (m, 5H), 7.15 (dd, *J* = 12.8, 6.5 Hz, 1H), 7.00 (s, 1H), 6.60 (dd, *J* = 10.8, 7.0 Hz, 1H), 6.46 (d, *J* = 8.8 Hz, 1H), 5.44 (s, 1H), 5.13 – 5.03 (m, 2H), 4.66 (t, *J* = 7.9 Hz, 1H), 4.61 (d, *J* = 8.9 Hz, 1H), 4.52 – 4.47 (m, 1H), 4.15 – 4.09 (m, 1H), 3.91 – 3.84 (m, 1H), 3.79 – 3.73 (m, 1H), 3.71 (s, 2H), 3.60 (dd, *J* = 11.3, 3.6 Hz, 1H), 3.58 – 3.49 (m, 5H), 3.34 (d, *J* = 12.5 Hz, 1H), 3.27 (d, *J* = 12.6 Hz, 1H), 3.22 – 3.14 (m, 2H), 2.52 (s, 3H), 2.51 – 2.47 (m, 5H), 2.21 – 2.13 (m, 2H), 2.08 – 2.01 (m, 1H), 2.01 – 1.91 (m, 1H), 1.91 – 1.80 (m, 1H), 1.60 – 1.56 (m, 4H), 1.48 (d, *J* = 7.0 Hz, 4H), 1.44 – 1.43 (m, 4H), 1.39 – 1.29 (m, 2H), 1.28 – 1.26 (m, 5H), 1.03 (s, 9H), 0.92 (s, 6H) ppm. LRMS (ESI) *m/z* $[M+H]^+$: calculated, 1082.6; found, 1082.6.

(2*S*,4*R*)-1-((*S*)-2-(10-((6-(4-(4-((4,4-dimethylpiperidin-1-yl)methyl)-2,5-difluorophenyl)-2-oxo-1,4,9-triazaspiro[5.5]undecan-9-yl)pyrimidin-4-yl)amino)decanamido)-3,3-dimethylbutanoyl)-4-hydroxy-*N*-((*S*)-1-(4-(4-methylthiazol-5-yl)phenyl)ethyl)pyrrolidine-2-carboxamide (ZW30406)

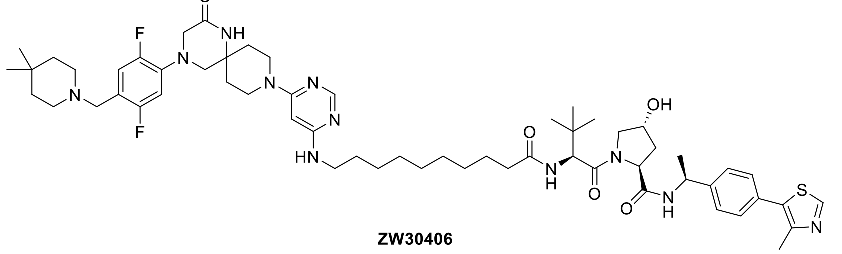

ZW30406

SnAr reaction between intermediate **2** and 10-aminodecanoic acid (10 eq.) afforded the acid intermediate in quantitative yield and the subsequent amide coupling with (2*S*,4*R*)-1-((*S*)-2-amino-3,3-dimethylbutanoyl)-4-hydroxy-*N*-((*S*)-1-(4-(4-methylthiazol-5-yl)phenyl)ethyl)pyrrolidine-2-carboxamide HCl salt in the presence of HATU and DIPEA in DMF gave the titled degrader ZW30406 as a white foam. ^{1}H-NMR (600 MHz, Chloroform-*d*) δ 8.67 (s, 1H), 8.07 (s, 1H), 7.56 (d, *J* = 7.8 Hz, 1H), 7.43 – 7.34 (m, 5H), 7.18 – 7.10 (m, 2H), 6.68 (d, *J* = 8.7 Hz, 1H), 6.60 (dd, *J* = 10.8, 7.1 Hz, 1H), 5.44 (s, 1H), 5.13 – 5.07 (m, 1H), 5.05 (s, 1H), 4.64 – 4.58 (m, 2H), 4.48 (s, 1H), 4.14 (d, *J* = 11.6 Hz, 1H), 3.95 – 3.86 (m, 1H), 3.72 (s, 3H), 3.63 – 3.51 (m, 5H), 3.37 (d, *J* = 12.5 Hz, 1H), 3.24 (d, *J* = 12.6 Hz, 1H), 3.21 – 3.14 (m, 2H), 2.52 (s, 3H), 2.49 (s, 4H), 2.43 – 2.38 (m, 1H), 2.18 (t, *J* = 8.3 Hz, 2H), 2.04 – 1.96 (m, 3H), 1.95 – 1.89 (m, 3H), 1.88 – 1.83 (m, 2H), 1.62 – 1.54 (m, 4H), 1.49 (d, *J* = 7.0 Hz, 4H), 1.46 – 1.41 (m, 6H), 1.38 – 1.34 (m, 2H), 1.32 – 1.29 (m, 2H), 1.04 (s, 9H), 0.92 (s, 6H) ppm. LRMS (ESI) *m/z* $[M+H]^{+}$: calculated, 1096.6; found, 1096.6.

(2*S*,4*R*)-1-((*S*)-2-(11-((6-(4-(4-((4,4-dimethylpiperidin-1-yl)methyl)-2,5-difluorophenyl)-2-oxo-1,4,9-triazaspiro[5.5]undecan-9-yl)pyrimidin-4-yl)amino)undecanamido)-3,3-dimethylbutanoyl)-4-hydroxy-*N*-((*S*)-1-(4-(4-methylthiazol-5-yl)phenyl)ethyl)pyrrolidine-2-carboxamide (ZW30407)

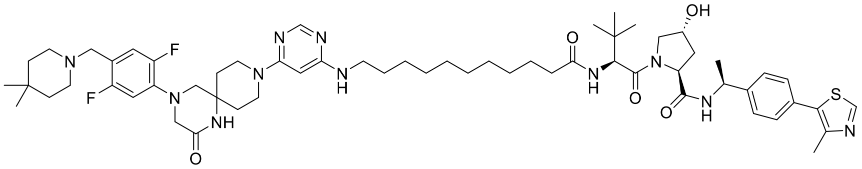

ZW30407

SnAr reaction between intermediate **2** and 11-aminoundecanoic acid (10 eq.) afforded the acid intermediate in quantitative yield and the subsequent amide coupling with (2*S*,4*R*)-1-((*S*)-2-amino-3,3-dimethylbutanoyl)-4-hydroxy-*N*-((*S*)-1-(4-(4-methylthiazol-5-yl)phenyl)ethyl)pyrrolidine-2-carboxamide HCl salt in the presence of HATU and DIPEA in

DMF gave the titled degrader ZW30407 as a white foam. ^{1}H-NMR (600 MHz, Chloroform-*d*) δ 8.67 (s, 1H), 8.07 (s, 1H), 7.55 (d, *J* = 7.8 Hz, 1H), 7.41 – 7.33 (m, 5H), 7.20 (s, 1H), 7.14 (dd, *J* = 12.8, 6.6 Hz, 1H), 6.60 (dd, *J* = 11.0, 6.9 Hz, 2H), 5.44 (s, 1H), 5.12 – 5.04 (m, 2H), 4.67 – 4.57 (m, 2H), 4.49 (s, 1H), 4.10 (d, *J* = 11.7 Hz, 1H), 3.91 – 3.83 (m, 1H), 3.71 (s, 3H), 3.60 (dd, *J* = 11.2, 3.7 Hz, 2H), 3.58 – 3.50 (m, 4H), 3.34 (d, *J* = 12.5 Hz, 1H), 3.25 (d, *J* = 12.5 Hz, 1H), 3.19 – 3.14 (m, 3H), 2.52 (s, 3H), 2.49 (s, 4H), 2.43 – 2.37 (m, 1H), 2.20 – 2.15 (m, 2H), 2.04 – 2.00 (m, 1H), 2.00 – 1.91 (m, 2H), 1.91 – 1.83 (m, 2H), 1.61 – 1.54 (m, 4H), 1.48 (d, *J* = 7.0 Hz, 4H), 1.44 – 1.40 (m, 6H), 1.38 – 1.33 (m, 2H), 1.32 – 1.26 (m, 4H), 1.03 (s, 9H), 0.92 (s, 6H) ppm. LRMS (ESI) *m/z* [M+H]$^+$: calculated, 1110.6; found, 1110.5.

(2*S*,4*R*)-1-((*S*)-2-(12-((6-(4-(4-((4,4-dimethylpiperidin-1-yl)methyl)-2,5-difluorophenyl)-2-oxo-1,4,9-triazaspiro[5.5]undecan-9-yl)pyrimidin-4-yl)amino)dodecanamido)-3,3-dimethylbutanoyl)-4-hydroxy-*N*-((*S*)-1-(4-(4-methylthiazol-5-yl)phenyl)ethyl)pyrrolidine-2-carboxamide (ZW30408)

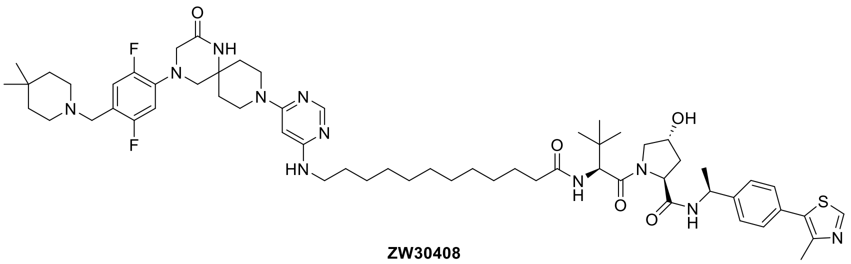

ZW30408

SnAr reaction between intermediate **2** and 12-aminododecanoic acid (10 eq.) afforded the acid intermediate in quantitative yield and the subsequent amide coupling with (2*S*,4*R*)-1-((*S*)-2-amino-3,3-dimethylbutanoyl)-4-hydroxy-*N*-((*S*)-1-(4-(4-methylthiazol-5-yl)phenyl)ethyl)pyrrolidine-2-carboxamide HCl salt in the presence of HATU and DIPEA in DMF gave the titled degrader ZW30408 as a white foam. ^{1}H-NMR (600 MHz, Chloroform-*d*) δ 8.67 (s, 1H), 8.07 (s, 1H), 7.54 (d, *J* = 7.8 Hz, 1H), 7.41 – 7.33 (m, 4H), 7.21 (s, 1H), 7.13 (dd, *J* = 12.8, 6.6 Hz, 1H), 6.65 (d, *J* = 8.9 Hz, 1H), 6.59 (dd, *J* = 10.9, 7.1 Hz, 1H), 5.43 (s, 1H), 5.13 – 5.04 (m, 2H), 4.62 (dd, *J* = 19.5, 8.5 Hz, 2H), 4.48 (s, 1H), 4.11 (d, *J* = 11.5 Hz, 1H), 3.91 – 3.82 (m, 1H), 3.71 (s, 3H), 3.60 (dd, *J* = 11.3, 3.6 Hz, 2H), 3.55 (s, 3H), 3.33 (d, *J* = 12.5 Hz, 1H), 3.24 (d, *J* = 12.5 Hz, 1H), 3.19 – 3.14 (m, 2H), 2.52 (s, 3H), 2.47 (s, 4H), 2.42 – 2.35 (m, 2H), 2.17 (t, *J* = 7.8 Hz, 2H), 2.04 – 1.94 (m, 2H), 1.93 – 1.84 (m, 2H), 1.62 – 1.54 (m, 4H), 1.48 (d, *J* = 6.9 Hz, 3H), 1.45 – 1.39 (m, 5H), 1.38 – 1.30 (m, 4H), 1.26 – 1.22 (m, 10H), 1.03 (s, 9H), 0.91 (s, 6H) ppm. LRMS (ESI) *m/z* [M+H]$^+$: calculated, 1124.7; found, 1124.5.

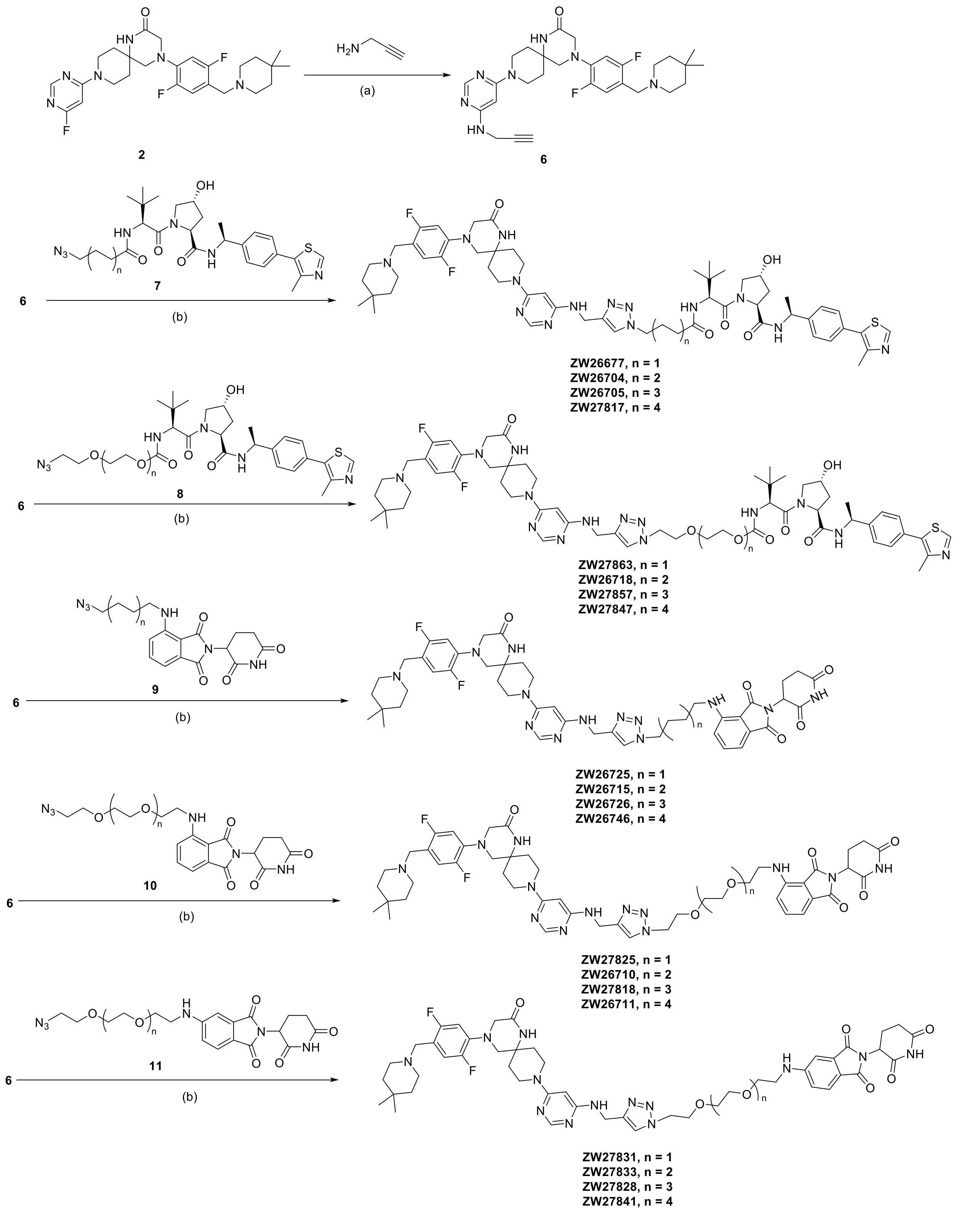

Scheme 2. Synthesis of METTL3 degraders containing a triazole handle[a]

[a]Reagents and conditions: (a) propargylamine, 130ºC, microwave irradiation, 1 h; (b) $CuSO_4 \cdot 5H_2O$, sodium ascorbate, THF, H_2O, rt, 4 hrs

4-(4-((4,4-dimethylpiperidin-1-yl)methyl)-2,5-difluorophenyl)-9-(6-(prop-2-yn-1-ylamino)pyrimidin-4-yl)-1,4,9-triazaspiro[5.5]undecan-2-one (6)

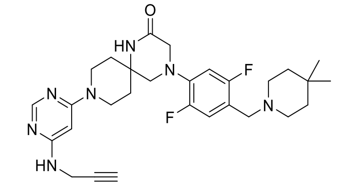

6

A suspension of 4-(4-((4,4-dimethylpiperidin-1-yl)methyl)-2,5-difluorophenyl)-9-(6-fluoropyrimidin-4-yl)-1,4,9-triazaspiro[5.5]undecan-2-one (100 mg, 0.2 mmol) in propargylamine (0.5 mL) was stirred at 130 °C under microwave irradiation and nitrogen atmosphere for 1 hour. The reaction was cooled down to room temperature and sonicated for 2 minutes. The precipitated solid was collected by filtration, washed with acetone (1 mL) and dried in vacuum to give the desired product (80 mg, 75% yield) as an off-white powder. ^{1}H-NMR (600 MHz, $CDCl_3$) δ 8.22 (d, J = 0.9 Hz, 1H), 7.12 (dd, J = 12.5, 6.3 Hz, 1H), 6.59 (dd, J = 10.8, 7.1 Hz, 1H), 6.26 (s, 1H), 5.57 (d, J = 1.1 Hz, 1H), 4.84 (t, J = 5.8 Hz, 1H), 4.09 (dd, J = 5.8, 2.5 Hz, 2H), 3.77 – 3.69 (m, 4H), 3.66 – 3.59 (m, 2H), 3.48 (s, 2H), 3.29 (s, 2H), 2.41 (s, 4H), 2.26 (t, J = 2.5 Hz, 1H), 2.02 – 1.95 (m, 2H), 1.86 – 1.78 (m, 2H), 1.39 (t, J = 5.7 Hz, 4H), 0.91 (s, 6H) ppm. LRMS (ESI) m/z $[M+H]^+$: calculated, 538.3; found, 538.3.

(2*S*,4*R*)-1-((*S*)-2-(4-(4-((((6-(4-(4-((4,4-dimethylpiperidin-1-yl)methyl)-2,5-difluorophenyl)-2-oxo-1,4,9-triazaspiro[5.5]undecan-9-yl)pyrimidin-4-yl)amino)methyl)-1*H*-1,2,3-triazol-1-yl)butanamido)-3,3-dimethylbutanoyl)-4-hydroxy-*N*-((*S*)-1-(4-(4-methylthiazol-5-yl)phenyl)ethyl)pyrrolidine-2-carboxamide (ZW26677)

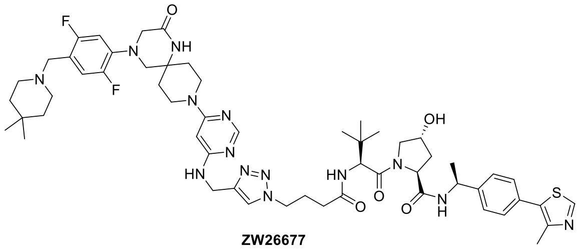

ZW26677

To a solution of intermediate **6** (20 mg, 37 μmol) and (2*S*,4*R*)-1-((*S*)-2-(4-azidobutanamido)-3,3-dimethylbutanoyl)-4-hydroxy-*N*-((*S*)-1-(4-(4-methylthiazol-5-yl)phenyl)ethyl)pyrrolidine-2-carboxamide (20 mg, 37 μmol) in THF (0.3 mL) was added $CuSO_4 \cdot 5H_2O$ (2.0 mg, 7.4 μmol), sodium ascorbate (1.5 mg, 7.4 μmol) and H_2O (0.1 mL). The resulting mixture was stirred at room temperature for 4 hours. The reaction mixture was partitioned between DCM and saturated aqueous NH_4Cl solution. The organic layer was washed with water and brine, dried over anhydrous Na_2SO_4, filtered, and concentrated. The oil residue was purified by prep-TLC

(DCM/MeOH = 10 : 1) to give the desired product (5 mg, 13% yield) as a pale-yellow foam. ^{1}H-NMR (600 MHz, Chloroform-*d*) δ 8.67 (s, 1H), 8.02 (s, 1H), 7.97 (s, 1H), 7.47 (s, 1H), 7.44 – 7.38 (m, 4H), 7.12 (dd, *J* = 12.8, 6.5 Hz, 1H), 6.58 (dd, *J* = 10.7, 7.1 Hz, 1H), 5.46 (s, 1H), 5.18 – 5.11 (m, 1H), 4.66 – 4.53 (m, 2H), 4.48 (s, 1H), 4.43 – 4.31 (m, 2H), 4.30 – 4.24 (m, 1H), 4.18 – 4.01 (m, 2H), 3.69 (s, 2H), 3.66 – 3.57 (m, 2H), 3.51 – 3.44 (m, 3H), 3.42 – 3.29 (m, 2H), 3.17 – 3.08 (m, 1H), 2.52 (s, 3H), 2.41 (br s, 4H), 2.27 – 2.16 (m, 3H), 2.12 – 2.02 (m, 2H), 2.01 – 1.94 (m, 2H), 1.83 – 1.69 (m, 8H), 1.52 (d, *J* = 7.0 Hz, 3H), 1.39 (t, *J* = 5.6 Hz, 4H), 1.03 (s, 9H), 0.91 (s, 6H) ppm. LRMS (ESI) *m/z* $[M+H]^+$: calculated, 1093.6; found, 1093.6.

(2*S*,4*R*)-1-((*S*)-2-(6-(4-(((6-(4-(4-((4,4-dimethylpiperidin-1-yl)methyl)-2,5-difluorophenyl)-2-oxo-1,4,9-triazaspiro[5.5]undecan-9-yl)pyrimidin-4-yl)amino)methyl)-1*H*-1,2,3-triazol-1-yl)hexanamido)-3,3-dimethylbutanoyl)-4-hydroxy-*N*-((*S*)-1-(4-(4-methylthiazol-5-yl)phenyl)ethyl)pyrrolidine-2-carboxamide (ZW26704)

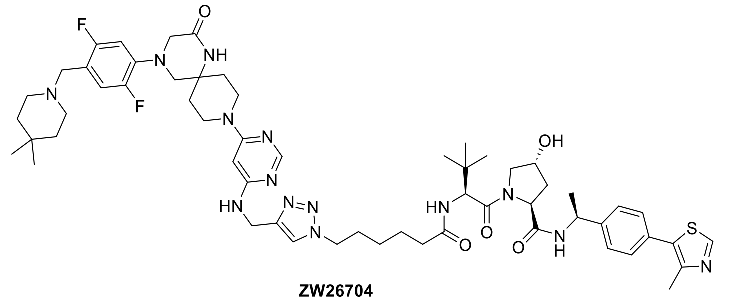

ZW26704

ZW26704 was synthesized from intermediate **6** and (2*S*,4*R*)-1-((*S*)-2-(6-azidohexanamido)-3,3-dimethylbutanoyl)-4-hydroxy-*N*-((*S*)-1-(4-(4-methylthiazol-5-yl)phenyl)ethyl)pyrrolidine-2-carboxamide using the same procedure described for ZW26677. ^{1}H-NMR (600 MHz, Chloroform-*d*) δ 8.66 (s, 1H), 8.05 (s, 1H), 7.80 (s, 2H), 7.45 (s, 1H), 7.40 (s, 4H), 7.11 (dd, *J* = 12.8, 6.6 Hz, 1H), 6.58 (dd, *J* = 10.7, 7.1 Hz, 1H), 5.78 (s, 1H), 5.58 (s, 1H), 5.16 – 5.09 (m, 1H), 4.72 – 4.62 (m, 2H), 4.51 – 4.43 (m, 2H), 4.37 (t, *J* = 8.1 Hz, 1H), 4.30 – 4.16 (m, 2H), 4.11 (d, *J* = 11.3 Hz, 1H), 4.05 (s, 1H), 3.73 – 3.65 (m, 2H), 3.61 (dd, *J* = 11.3, 3.4 Hz, 1H), 3.58 – 3.50 (m, 2H), 3.49 – 3.47 (m, 2H), 3.44 (d, *J* = 12.2 Hz, 2H), 3.12 (d, *J* = 12.5 Hz, 1H), 2.52 (s, 3H), 2.44 – 2.36 (m, 4H), 2.26 – 2.08 (m, 4H), 2.00 – 1.90 (m, 5H), 1.87 – 1.70 (m, 5H), 1.60 – 1.54 (m, 1H), 1.52 (d, *J* = 6.9 Hz, 3H), 1.50 – 1.44 (m, 1H), 1.39 (t, *J* = 5.6 Hz, 4H), 1.03 (s, 9H), 0.91 (s, 6H) ppm. LRMS (ESI) *m/z* $[M+H]^+$: calculated, 1121.6; found, 1121.8.

(2*S*,4*R*)-1-((*S*)-2-(8-(4-(((6-(4-(4-((4,4-dimethylpiperidin-1-yl)methyl)-2,5-difluorophenyl)-2-oxo-1,4,9-triazaspiro[5.5]undecan-9-yl)pyrimidin-4-yl)amino)methyl)-1*H*-1,2,3-triazol-1-

yl)octanamido)-3,3-dimethylbutanoyl)-4-hydroxy-*N*-((*S*)-1-(4-(4-methylthiazol-5-yl)phenyl)ethyl)pyrrolidine-2-carboxamide (ZW26705)

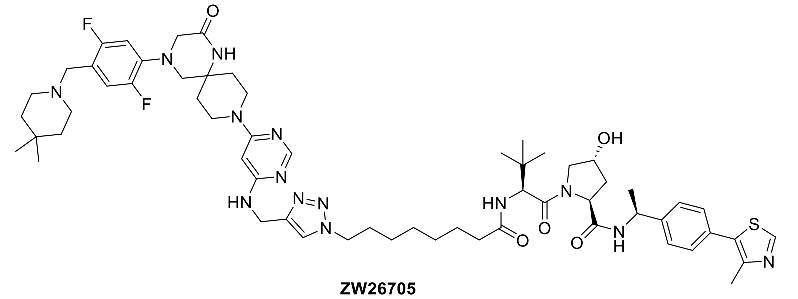

ZW26705

ZW26705 was synthesized from intermediate **6** and (2*S*,4*R*)-1-((*S*)-2-(8-azidooctanamido)-3,3-dimethylbutanoyl)-4-hydroxy-*N*-((*S*)-1-(4-(4-methylthiazol-5-yl)phenyl)ethyl)pyrrolidine-2-carboxamide using the same procedure described for ZW26677. ^{1}H-NMR (600 MHz, Chloroform-*d*) δ 8.67 (s, 1H), 8.09 (s, 1H), 7.62 (d, *J* = 7.8 Hz, 1H), 7.51 (s, 1H), 7.42 – 7.37 (m, 4H), 7.34 (s, 1H), 7.10 (dd, *J* = 12.8, 6.6 Hz, 1H), 6.89 (s, 1H), 6.58 (dd, *J* = 10.8, 7.1 Hz, 1H), 5.89 (s, 1H), 5.60 (s, 1H), 5.14 – 5.07 (m, 1H), 4.69 – 4.60 (m, 2H), 4.56 – 4.49 (m, 2H), 4.47 (s, 1H), 4.35 – 4.26 (m, 2H), 4.12 (d, *J* = 11.3 Hz, 1H), 3.93 – 3.85 (m, 1H), 3.70 (d, *J* = 2.0 Hz, 2H), 3.63 – 3.54 (m, 3H), 3.50 – 3.43 (m, 3H), 3.34 (d, *J* = 12.5 Hz, 1H), 3.17 (d, *J* = 12.4 Hz, 1H), 2.52 (s, 3H), 2.40 (d, *J* = 3.8 Hz, 4H), 2.33 – 2.26 (m, 1H), 2.18 – 2.08 (m, 2H), 2.00 – 1.91 (m, 2H), 1.87 (t, *J* = 6.1 Hz, 2H), 1.85 – 1.78 (m, 4H), 1.50 (d, *J* = 7.0 Hz, 3H), 1.48 – 1.45 (m, 1H), 1.39 (t, *J* = 5.6 Hz, 4H), 1.22 – 1.13 (m, 6H), 1.04 (s, 9H), 0.91 (s, 6H) ppm. LRMS (ESI) *m/z* $[M+H]^+$: calculated, 1149.6; found, 1149.5.

(2*S*,4*R*)-1-((*S*)-2-(10-(4-(((6-(4-(4-((4,4-dimethylpiperidin-1-yl)methyl)-2,5-difluorophenyl)-2-oxo-1,4,9-triazaspiro[5.5]undecan-9-yl)pyrimidin-4-yl)amino)methyl)-1*H*-1,2,3-triazol-1-yl)decanamido)-3,3-dimethylbutanoyl)-4-hydroxy-*N*-((*S*)-1-(4-(4-methylthiazol-5-yl)phenyl)ethyl)pyrrolidine-2-carboxamide (ZW27817)

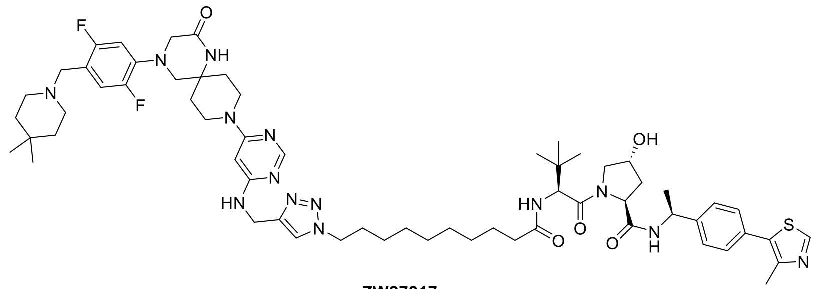

ZW27817

ZW27817 was synthesized from intermediate **6** and (2*S*,4*R*)-1-((*S*)-2-(10-azidodecanamido)-3,3-dimethylbutanoyl)-4-hydroxy-*N*-((*S*)-1-(4-(4-methylthiazol-5-yl)phenyl)ethyl)pyrrolidine-2-carboxamide using the same procedure described for ZW26677. ^{1}H-NMR (600 MHz, Chloroform-*d*) δ 8.67 (s, 1H), 8.12 (s, 1H), 7.54 (d, *J* = 7.8 Hz, 1H), 7.51 (s, 1H), 7.38 (q, *J* = 8.4 Hz, 4H), 7.18 – 7.08 (m, 2H), 6.80 (d, *J* = 8.9 Hz, 1H), 6.58 (dd, *J* = 10.8, 7.1 Hz, 1H), 5.82 (s, 1H), 5.59 (s, 1H), 5.13 – 5.06 (m, 1H), 4.66 – 4.59 (m, 3H), 4.58 – 4.52 (m, 1H), 4.51 – 4.47 (m, 1H), 4.35 – 4.26 (m, 2H), 4.15 – 4.11 (m, 1H), 3.85 – 3.79 (m, 1H), 3.70 (s, 2H), 3.68 – 3.62 (m, 1H), 3.60 (dd, *J* = 11.3, 3.6 Hz, 1H), 3.52 – 3.46 (m, 3H), 3.31 (d, *J* = 12.5 Hz, 1H), 3.21 (d, *J* = 12.5 Hz, 1H), 2.52 (s, 3H), 2.42 (s, 4H), 2.19 – 2.11 (m, 3H), 2.05 – 2.00 (m, 2H), 1.97 – 1.88 (m, 3H), 1.88 – 1.80 (m, 4H), 1.55 – 1.51 (m, 1H), 1.48 (d, *J* = 7.0 Hz, 3H), 1.40 (t, *J* = 5.6 Hz, 4H), 1.23 – 1.13 (m, 10H), 1.04 (s, 9H), 0.91 (s, 6H) ppm. LRMS (ESI) *m/z* $[M+H]^{+}$: calculated, 1177.7; found, 1177.6.

(2*S*,4*R*)-1-((*S*)-2-(2-(2-(2-(4-(((6-(4-(4-((4,4-dimethylpiperidin-1-yl)methyl)-2,5-difluorophenyl)-2-oxo-1,4,9-triazaspiro[5.5]undecan-9-yl)pyrimidin-4-yl)amino)methyl)-1*H*-1,2,3-triazol-1-yl)ethoxy)ethoxy)acetamido)-3,3-dimethylbutanoyl)-4-hydroxy-*N*-((*S*)-1-(4-(4-methylthiazol-5-yl)phenyl)ethyl)pyrrolidine-2-carboxamide (ZW27863)

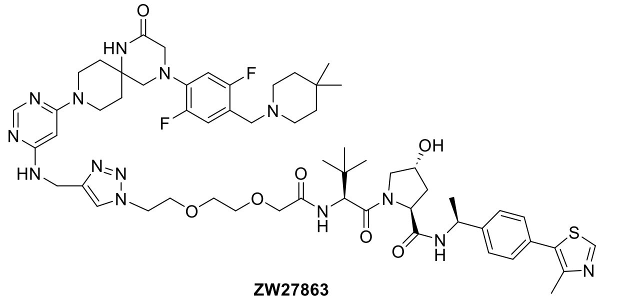

ZW27863

ZW27863 was synthesized from intermediate **6** and (2*S*,4*R*)-1-((*S*)-2-(2-(2-(2-azidoethoxy)ethoxy)acetamido)-3,3-dimethylbutanoyl)-4-hydroxy-*N*-((*S*)-1-(4-(4-methylthiazol-5-yl)phenyl)ethyl)pyrrolidine-2-carboxamide using the same procedure described for ZW26677. ^{1}H-NMR (600 MHz, Chloroform-*d*) δ 8.67 (s, 1H), 8.09 (s, 1H), 7.80 (s, 1H), 7.65 (d, *J* = 7.7 Hz, 1H), 7.38 (d, *J* = 8.3 Hz, 2H), 7.34 (d, *J* = 8.3 Hz, 2H), 7.21 (s, 1H), 6.81 (s, 1H), 6.59 (dd, *J* = 10.9, 6.9 Hz, 1H), 6.14 (s, 1H), 5.61 (s, 1H), 5.14 – 5.03 (m, 1H), 4.69 (t, *J* = 8.1 Hz, 1H), 4.64 (d, *J* = 9.4 Hz, 1H), 4.59 (d, *J* = 6.0 Hz, 2H), 4.55 – 4.51 (m, 2H), 4.09 (d, *J* = 11.3 Hz, 1H), 3.92 – 3.86 (m, 1H), 3.85 – 3.79 (m, 3H), 3.76 – 3.69 (m, 4H), 3.68 – 3.62 (m, 3H), 3.62 – 3.55 (m, 3H), 3.55 – 3.47 (m, 3H), 3.33 – 3.26 (m, 2H), 2.58 (br s, 3H), 2.52 (s, 3H), 2.41 – 2.35 (m, 1H),

2.15 – 2.08 (m, 2H), 1.96 – 1.88 (m, 4H), 1.83 – 1.75 (m, 3H), 1.48 (s, 4H), 1.44 (d, *J* = 6.9 Hz, 3H), 1.03 (s, 9H), 0.94 (s, 6H) ppm. LRMS (ESI) *m/z* $[M+H]^+$: calculated, 1153.6; found, 1153.8.

(2*S*,4*R*)-1-((*S*)-2-(tert-butyl)-14-(4-(((6-(4-(4-((4,4-dimethylpiperidin-1-yl)methyl)-2,5-difluorophenyl)-2-oxo-1,4,9-triazaspiro[5.5]undecan-9-yl)pyrimidin-4-yl)amino)methyl)-1*H*-1,2,3-triazol-1-yl)-4-oxo-6,9,12-trioxa-3-azatetradecanoyl)-4-hydroxy-*N*-((*S*)-1-(4-(4-methylthiazol-5-yl)phenyl)ethyl)pyrrolidine-2-carboxamide (ZW26718)

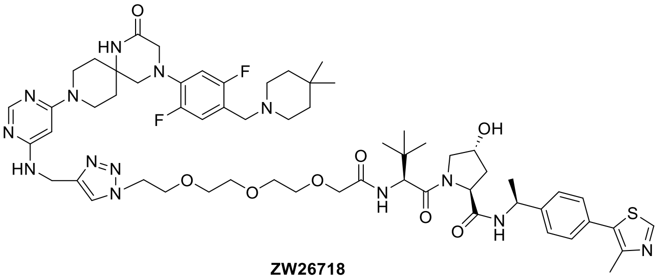

ZW26718

ZW26718 was synthesized from intermediate **6** and (2*S*,4*R*)-1-((*S*)-14-azido-2-(tert-butyl)-4-oxo-6,9,12-trioxa-3-azatetradecanoyl)-4-hydroxy-*N*-((*S*)-1-(4-(4-methylthiazol-5-yl)phenyl)ethyl)pyrrolidine-2-carboxamide using the same procedure described for ZW26677. ^{1}H-NMR (600 MHz, Chloroform-*d*) δ 8.67 (s, 1H), 8.09 (s, 1H), 7.81 (s, 1H), 7.62 (d, *J* = 7.8 Hz, 1H), 7.39 (d, *J* = 8.3 Hz, 2H), 7.36 (d, *J* = 8.4 Hz, 2H), 7.32 (d, *J* = 9.2 Hz, 1H), 7.12 (dd, *J* = 12.8, 6.5 Hz, 1H), 7.00 (s, 1H), 6.58 (dd, *J* = 10.8, 7.1 Hz, 1H), 5.88 (s, 1H), 5.63 (s, 1H), 5.30 (s, 1H), 5.14 – 5.03 (m, 1H), 4.66 (t, *J* = 8.1 Hz, 1H), 4.62 (d, *J* = 9.2 Hz, 1H), 4.54 (d, *J* = 5.9 Hz, 2H), 4.53 – 4.49 (m, 3H), 4.11 (d, *J* = 11.0 Hz, 1H), 3.93 – 3.86 (m, 2H), 3.86 – 3.81 (m, 3H), 3.81 – 3.75 (m, 1H), 3.69 (s, 2H), 3.64 – 3.60 (m, 2H), 3.60 – 3.55 (m, 7H), 3.51 (s, 2H), 3.50 – 3.44 (m, 2H), 3.31 (d, *J* = 12.5 Hz, 1H), 3.25 (d, *J* = 12.6 Hz, 1H), 2.52 (s, 3H), 2.44 (br s, 4H), 2.12 – 2.03 (m, 2H), 1.97 – 1.89 (m, 2H), 1.85 – 1.74 (m, 2H), 1.47 (d, *J* = 6.9 Hz, 3H), 1.41 (t, *J* = 5.7 Hz, 4H), 1.04 (s, 9H), 0.91 (s, 6H) ppm. LRMS (ESI) *m/z* $[M+H]^+$: calculated, 1197.6; found, 1197.6.

(2*S*,4*R*)-1-((*S*)-2-(tert-butyl)-17-(4-(((6-(4-(4-((4,4-dimethylpiperidin-1-yl)methyl)-2,5-difluorophenyl)-2-oxo-1,4,9-triazaspiro[5.5]undecan-9-yl)pyrimidin-4-yl)amino)methyl)-1*H*-1,2,3-triazol-1-yl)-4-oxo-6,9,12,15-tetraoxa-3-azaheptadecanoyl)-4-hydroxy-*N*-((*S*)-1-(4-(4-methylthiazol-5-yl)phenyl)ethyl)pyrrolidine-2-carboxamide (ZW27857)

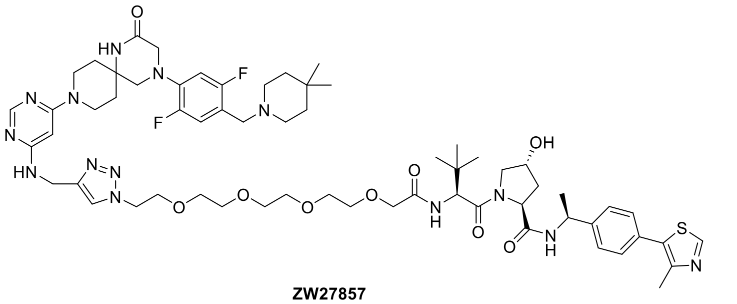

ZW27857

ZW27857 was synthesized from intermediate **6** and (2*S*,4*R*)-1-((*S*)-17-azido-2-(tert-butyl)-4-oxo-6,9,12,15-tetraoxa-3-azaheptadecanoyl)-4-hydroxy-*N*-((*S*)-1-(4-(4-methylthiazol-5-yl)phenyl)ethyl)pyrrolidine-2-carboxamide using the same procedure described for ZW26677. ^{1}H-NMR (600 MHz, Chloroform-*d*) δ 8.67 (s, 1H), 8.09 (s, 1H), 7.81 (s, 2H), 7.39 (d, J = 8.3 Hz, 2H), 7.37 (d, J = 8.3 Hz, 2H), 7.30 (d, J = 9.0 Hz, 1H), 7.20 (s, 1H), 6.95 (s, 1H), 6.58 (dd, J = 10.9, 7.0 Hz, 1H), 5.92 (s, 1H), 5.62 (s, 1H), 5.13 – 5.06 (m, 1H), 4.70 (t, J = 8.1 Hz, 1H), 4.61 – 4.54 (m, 3H), 4.54 – 4.48 (m, 3H), 4.07 (d, J = 11.2 Hz, 1H), 3.95 – 3.88 (m, 2H), 3.86 – 3.83 (m, 2H), 3.83 – 3.75 (m, 2H), 3.70 (s, 2H), 3.65 (d, J = 3.3 Hz, 1H), 3.63 (t, J = 2.8 Hz, 4H), 3.62 – 3.54 (m, 10H), 3.52 – 3.43 (m, 2H), 3.34 – 3.25 (m, 2H), 2.52 (s, 3H), 2.42 – 2.35 (m, 1H), 2.14 – 2.04 (m, 2H), 1.99 – 1.88 (m, 4H), 1.84 – 1.75 (m, 4H), 1.48 (d, J = 6.9 Hz, 3H), 1.46 (s, 4H), 1.03 (s, 9H), 0.93 (s, 6H) ppm. LRMS (ESI) *m/z* [M+H]$^+$: calculated, 1241.6; found, 1241.6.

(2*S*,4*R*)-1-((*S*)-2-(tert-butyl)-20-(4-(((6-(4-(4-((4,4-dimethylpiperidin-1-yl)methyl)-2,5-difluorophenyl)-2-oxo-1,4,9-triazaspiro[5.5]undecan-9-yl)pyrimidin-4-yl)amino)methyl)-1*H*-1,2,3-triazol-1-yl)-4-oxo-6,9,12,15,18-pentaoxa-3-azaicosanoyl)-4-hydroxy-*N*-((*S*)-1-(4-(4-methylthiazol-5-yl)phenyl)ethyl)pyrrolidine-2-carboxamide (ZW27847)

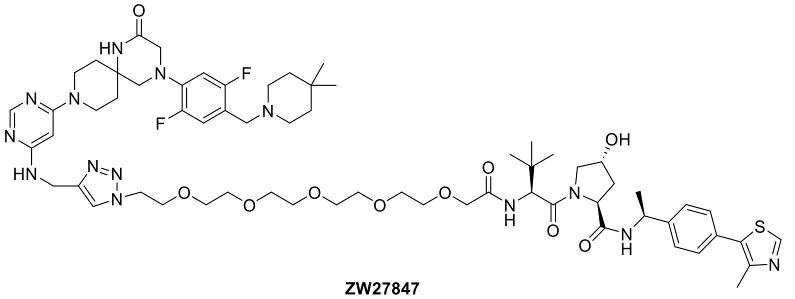

ZW27847

ZW27847 was synthesized from intermediate **6** and (2*S*,4*R*)-1-((*S*)-20-azido-2-(tert-butyl)-4-oxo-6,9,12,15,18-pentaoxa-3-azaicosanoyl)-4-hydroxy-*N*-((*S*)-1-(4-(4-methylthiazol-5-yl)phenyl)ethyl)pyrrolidine-2-carboxamide using the same procedure described for ZW26677.

^{1}H-NMR (600 MHz, Chloroform-*d*) δ 8.67 (s, 1H), 8.11 (s, 1H), 7.81 (s, 1H), 7.76 (d, *J* = 7.8 Hz, 1H), 7.39 (d, *J* = 8.5 Hz, 2H), 7.37 (d, *J* = 8.4 Hz, 2H), 7.32 (d, *J* = 8.9 Hz, 1H), 7.15 (dd, *J* = 12.8, 6.5 Hz, 1H), 7.02 (s, 1H), 6.59 (dd, *J* = 10.8, 7.1 Hz, 1H), 5.86 (s, 1H), 5.64 (s, 1H), 5.13 – 5.06 (m, 1H), 4.70 (t, *J* = 8.0 Hz, 1H), 4.62 (d, *J* = 9.1 Hz, 1H), 4.57 (d, *J* = 5.4 Hz, 2H), 4.53 – 4.48 (m, 3H), 4.09 (d, *J* = 11.2 Hz, 1H), 3.93 – 3.86 (m, 2H), 3.85 – 3.83 (m, 2H), 3.80 – 3.74 (m, 1H), 3.69 (s, 2H), 3.65 – 3.60 (m, 10H), 3.59 – 3.53 (m, 8H), 3.51 – 3.43 (m, 2H), 3.33 – 3.23 (m, 2H), 2.52 (s, 3H), 2.48 (s, 4H), 2.43 – 2.37 (m, 1H), 2.11 – 2.05 (m, 2H), 1.97 – 1.89 (m, 4H), 1.84 – 1.77 (m, 2H), 1.48 (d, *J* = 7.0 Hz, 3H), 1.44 – 1.40 (m, 4H), 1.03 (s, 9H), 0.92 (s, 6H) ppm. LRMS (ESI) *m/z* $[M+H]^+$: calculated, 1285.7; found, 1285.7.

4-((4-(4-(((6-(4-(4-((4,4-dimethylpiperidin-1-yl)methyl)-2,5-difluorophenyl)-2-oxo-1,4,9-triazaspiro[5.5]undecan-9-yl)pyrimidin-4-yl)amino)methyl)-1*H*-1,2,3-triazol-1-yl)butyl)amino)-2-(2,6-dioxopiperidin-3-yl)isoindoline-1,3-dione (ZW26725)

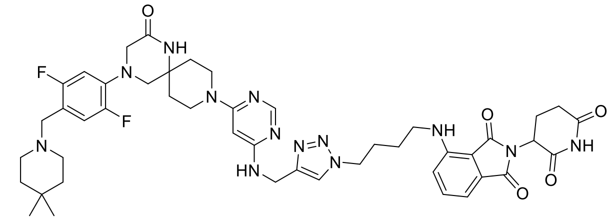

ZW26725

ZW26725 was synthesized from intermediate **6** and 4-((4-azidobutyl)amino)-2-(2,6-dioxopiperidin-3-yl)isoindoline-1,3-dione using the same procedure described for ZW26677. ^{1}H-NMR (600 MHz, Chloroform-*d*) δ 8.21 (d, *J* = 0.9 Hz, 1H), 7.52 – 7.46 (m, 2H), 7.17 – 7.05 (m, 2H), 6.83 (d, *J* = 8.5 Hz, 1H), 6.78 (s, 1H), 6.57 (dd, *J* = 10.8, 7.1 Hz, 1H), 6.25 (t, *J* = 5.9 Hz, 1H), 5.49 (s, 1H), 4.96 – 4.86 (m, 1H), 4.62 – 4.47 (m, 2H), 4.42 – 4.27 (m, 2H), 3.72 (s, 2H), 3.70 – 3.63 (m, 2H), 3.62 – 3.53 (m, 2H), 3.51 (s, 2H), 3.39 – 3.31 (m, 1H), 3.30 – 3.23 (m, 3H), 2.90 – 2.84 (m, 1H), 2.79 – 2.69 (m, 2H), 2.43 (s, 4H), 2.17 – 2.10 (m, 1H), 2.04 – 1.97 (m, 2H), 1.95 – 1.87 (m, 2H), 1.82 – 1.74 (m, 4H), 1.68 – 1.65 (m, 2H), 1.40 (t, *J* = 5.7 Hz, 4H), 0.91 (s, 6H) ppm. LRMS (ESI) *m/z* $[M+H]^+$: calculated, 908.4; found, 908.3.

4-((6-(4-(((6-(4-(4-((4,4-dimethylpiperidin-1-yl)methyl)-2,5-difluorophenyl)-2-oxo-1,4,9-triazaspiro[5.5]undecan-9-yl)pyrimidin-4-yl)amino)methyl)-1*H*-1,2,3-triazol-1-yl)hexyl)amino)-2-(2,6-dioxopiperidin-3-yl)isoindoline-1,3-dione (ZW26715)

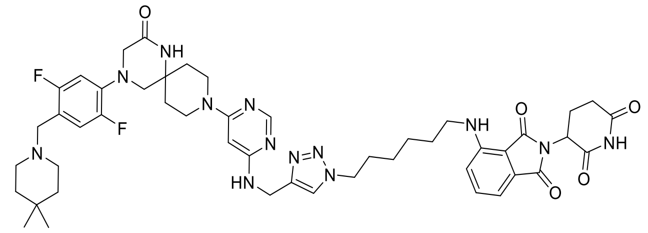

ZW26715 was synthesized from intermediate **6** and 4-((6-azidohexyl)amino)-2-(2,6-dioxopiperidin-3-yl)isoindoline-1,3-dione using the same procedure described for ZW26677. ^{1}H-NMR (600 MHz, Chloroform-*d*) δ 8.19 (s, 1H), 7.51 – 7.46 (m, 2H), 7.14 – 7.09 (m, 1H), 7.08 (d, *J* = 7.1 Hz, 1H), 6.85 (d, *J* = 8.6 Hz, 1H), 6.81 (s, 1H), 6.57 (dd, *J* = 10.8, 7.1 Hz, 1H), 6.23 (t, *J* = 5.8 Hz, 1H), 5.52 (s, 1H), 4.92 (dd, *J* = 12.4, 5.4 Hz, 1H), 4.56 (t, *J* = 6.7 Hz, 2H), 4.34 – 4.26 (m, 2H), 3.72 (s, 2H), 3.71 – 3.65 (m, 2H), 3.55 (td, *J* = 9.5, 8.6, 3.9 Hz, 2H), 3.50 (s, 2H), 3.28 – 3.22 (m, 4H), 2.89 – 2.84 (m, 1H), 2.82 – 2.69 (m, 2H), 2.42 (s, 4H), 2.15 – 2.10 (m, 1H), 2.01 (s, 2H), 1.94 – 1.87 (m, 4H), 1.81 – 1.75 (m, 2H), 1.65 – 1.59 (m, 2H), 1.48 – 1.37 (m, 6H), 1.36 – 1.30 (m, 2H), 0.91 (s, 6H) ppm. LRMS (ESI) *m/z* $[M+H]^+$: calculated, 936.5; found, 936.5.

4-((8-(4-(((6-(4-(4-((4,4-dimethylpiperidin-1-yl)methyl)-2,5-difluorophenyl)-2-oxo-1,4,9-triazaspiro[5.5]undecan-9-yl)pyrimidin-4-yl)amino)methyl)-1*H*-1,2,3-triazol-1-yl)octyl)amino)-2-(2,6-dioxopiperidin-3-yl)isoindoline-1,3-dione (ZW26726)

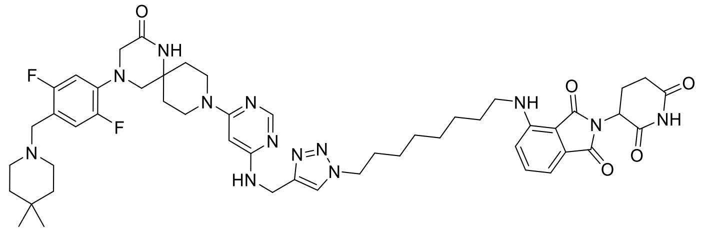

ZW26726 was synthesized from intermediate **6** and 4-((8-azidooctyl)amino)-2-(2,6-dioxopiperidin-3-yl)isoindoline-1,3-dione using the same procedure described for ZW26677. ^{1}H-NMR (600 MHz, Chloroform-*d*) δ 8.20 (s, 1H), 7.51 – 7.44 (m, 2H), 7.11 (dd, *J* = 12.8, 6.5 Hz, 1H), 7.08 (d, *J* = 7.0 Hz, 1H), 6.87 (d, *J* = 8.6 Hz, 1H), 6.67 (s, 1H), 6.58 (dd, *J* = 10.8, 7.1 Hz, 1H), 6.23 (t, *J* = 5.6 Hz, 1H), 5.82 (s, 1H), 5.53 (s, 1H), 4.91 (dd, *J* = 12.4, 5.3 Hz, 1H), 4.57 (d, *J* = 5.9 Hz, 2H), 4.36 – 4.23 (m, 2H), 3.72 (s, 2H), 3.71 – 3.65 (m, 2H), 3.58 – 3.52 (m, 2H), 3.49 (s, 2H), 3.29 – 3.22 (m, 4H), 2.91 – 2.69 (m, 3H), 2.42 (s, 4H), 2.15 – 2.09 (m, 1H), 1.96 – 1.90 (m, 2H), 1.88 – 1.82 (m, 3H), 1.82 – 1.76 (m, 4H), 1.66 – 1.60 (m, 2H), 1.44 – 1.36 (m,

6H), 1.35 – 1.29 (m, 4H), 0.91 (s, 6H) ppm. LRMS (ESI) *m/z* [M+H]$^+$: calculated, 964.5; found, 964.4.

4-((10-(4-(((6-(4-(4-((4,4-dimethylpiperidin-1-yl)methyl)-2,5-difluorophenyl)-2-oxo-1,4,9-triazaspiro[5.5]undecan-9-yl)pyrimidin-4-yl)amino)methyl)-1*H*-1,2,3-triazol-1-yl)decyl)amino)-2-(2,6-dioxopiperidin-3-yl)isoindoline-1,3-dione (ZW26746)

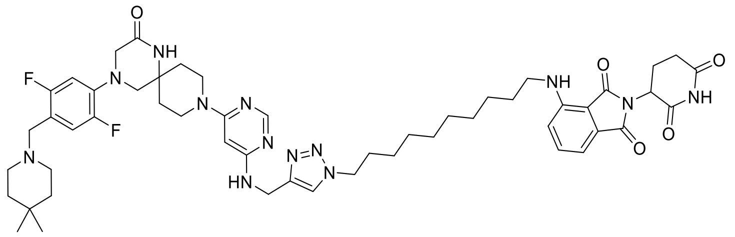

ZW26746

ZW26746 was synthesized from intermediate **6** and 4-((10-azidodecyl)amino)-2-(2,6-dioxopiperidin-3-yl)isoindoline-1,3-dione using the same procedure described for ZW26677. ^{1}H-NMR (600 MHz, Chloroform-*d*) δ 8.20 (s, 1H), 7.51 – 7.44 (m, 2H), 7.12 (dd, J = 12.8, 6.5 Hz, 1H), 7.07 (d, J = 7.0 Hz, 1H), 6.87 (d, J = 8.6 Hz, 1H), 6.74 (s, 1H), 6.58 (dd, J = 10.8, 7.1 Hz, 1H), 6.22 (t, J = 5.5 Hz, 1H), 5.81 (s, 1H), 5.54 (d, J = 1.0 Hz, 1H), 4.91 (dd, J = 12.3, 5.4 Hz, 1H), 4.57 (d, J = 5.9 Hz, 2H), 4.34 – 4.25 (m, 2H), 3.72 (s, 2H), 3.70 – 3.64 (m, 2H), 3.59 – 3.53 (m, 2H), 3.50 (s, 2H), 3.29 – 3.22 (m, 4H), 2.90 – 2.69 (m, 3H), 2.42 (s, 4H), 2.15 – 2.08 (m, 1H), 1.96 – 1.89 (m, 3H), 1.88 – 1.82 (m, 3H), 1.82 – 1.76 (m, 3H), 1.68 – 1.61 (m, 2H), 1.44 – 1.35 (m, 6H), 1.33 – 1.26 (m, 8H), 0.91 (s, 6H) ppm. LRMS (ESI) *m/z* [M+H]$^+$: calculated, 992.5; found, 992.6.

4-((2-(2-(2-(4-(((6-(4-(4-((4,4-dimethylpiperidin-1-yl)methyl)-2,5-difluorophenyl)-2-oxo-1,4,9-triazaspiro[5.5]undecan-9-yl)pyrimidin-4-yl)amino)methyl)-1*H*-1,2,3-triazol-1-yl)ethoxy)ethoxy)ethyl)amino)-2-(2,6-dioxopiperidin-3-yl)isoindoline-1,3-dione (ZW27825)

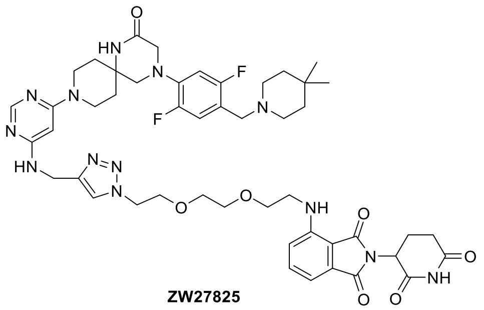

ZW27825

ZW27825 was synthesized from intermediate **6** and 4-((2-(2-(2-azidoethoxy)ethoxy)ethyl)amino)-2-(2,6-dioxopiperidin-3-yl)isoindoline-1,3-dione using the

same procedure described for ZW26677. ^{1}H-NMR (600 MHz, Chloroform-*d*) δ 8.16 (s, 1H), 7.70 (s, 1H), 7.47 (dd, *J* = 8.6, 7.1 Hz, 1H), 7.14 (s, 1H), 7.09 (d, *J* = 7.1 Hz, 1H), 6.90 (d, *J* = 8.5 Hz, 1H), 6.69 (s, 1H), 6.58 (dd, *J* = 10.8, 7.1 Hz, 1H), 6.48 (t, *J* = 5.6 Hz, 1H), 5.91 (s, 1H), 5.51 (s, 1H), 4.90 (dd, *J* = 12.5, 5.3 Hz, 1H), 4.54 – 4.50 (m, 2H), 4.49 – 4.44 (m, 2H), 3.88 – 3.81 (m, 2H), 3.72 (s, 2H), 3.67 (t, *J* = 5.1 Hz, 2H), 3.65 – 3.56 (m, 8H), 3.52 (s, 2H), 3.45 – 3.41 (m, 2H), 3.26 (s, 2H), 2.89 – 2.68 (m, 3H), 2.44 (s, 4H), 2.11 (s, 1H), 1.93 – 1.86 (m, 2H), 1.81 – 1.76 (m, 2H), 1.41 (s, 4H), 0.91 (s, 6H) ppm. LRMS (ESI) *m/z* [M+H]$^+$: calculated, 968.5; found, 968.7.

4-((2-(2-(2-(2-(4-(((6-(4-(4-((4,4-dimethylpiperidin-1-yl)methyl)-2,5-difluorophenyl)-2-oxo-1,4,9-triazaspiro[5.5]undecan-9-yl)pyrimidin-4-yl)amino)methyl)-1*H*-1,2,3-triazol-1-yl)ethoxy)ethoxy)ethoxy)ethyl)amino)-2-(2,6-dioxopiperidin-3-yl)isoindoline-1,3-dione (ZW26710)

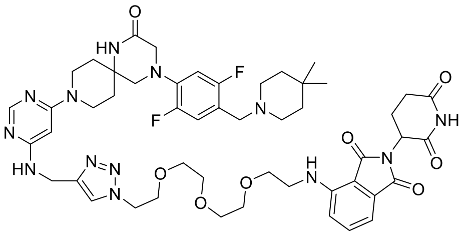

ZW26710

ZW26710 was synthesized from intermediate **6** and 4-((2-(2-(2-(2-azidoethoxy)ethoxy)ethoxy)ethyl)amino)-2-(2,6-dioxopiperidin-3-yl)isoindoline-1,3-dione using the same procedure described for ZW26677. ^{1}H-NMR (600 MHz, Chloroform-*d*) δ 8.19 (s, 1H), 7.71 (s, 1H), 7.47 (dd, *J* = 8.5, 7.1 Hz, 1H), 7.16 – 7.05 (m, 2H), 6.89 (d, *J* = 8.6 Hz, 1H), 6.64 (s, 1H), 6.57 (dd, *J* = 10.8, 7.1 Hz, 1H), 6.49 (t, *J* = 5.6 Hz, 1H), 5.84 (s, 1H), 5.52 (s, 1H), 4.91 (dd, *J* = 12.4, 5.4 Hz, 1H), 4.54 (d, *J* = 5.8 Hz, 2H), 4.51 – 4.43 (m, 2H), 3.82 (t, *J* = 5.1 Hz, 2H), 3.74 – 3.69 (m, 4H), 3.67 – 3.61 (m, 6H), 3.60 – 3.57 (m, 3H), 3.56 – 3.53 (m, 2H), 3.48 (d, *J* = 1.3 Hz, 2H), 3.44 (q, *J* = 5.4 Hz, 2H), 3.25 (s, 2H), 2.92 – 2.67 (m, 3H), 2.40 (s, 4H), 2.14 – 2.07 (m, 1H), 1.94 – 1.87 (m, 2H), 1.81 – 1.73 (m, 3H), 1.39 (t, *J* = 5.6 Hz, 4H), 0.91 (s, 6H) ppm. LRMS (ESI) *m/z* [M+H]$^+$: calculated, 1012.5; found, 1012.6.

4-((14-(4-(((6-(4-(4-((4,4-dimethylpiperidin-1-yl)methyl)-2,5-difluorophenyl)-2-oxo-1,4,9-triazaspiro[5.5]undecan-9-yl)pyrimidin-4-yl)amino)methyl)-1*H*-1,2,3-triazol-1-yl)-3,6,9,12-tetraoxatetradecyl)amino)-2-(2,6-dioxopiperidin-3-yl)isoindoline-1,3-dione (ZW27818)

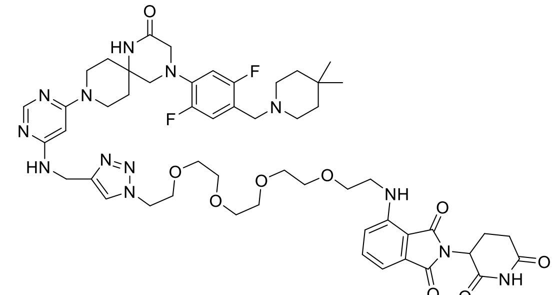

ZW27818

ZW27818 was synthesized from intermediate **6** and 4-((14-azido-3,6,9,12-tetraoxatetradecyl)amino)-2-(2,6-dioxopiperidin-3-yl)isoindoline-1,3-dione using the same procedure described for ZW26677. ^{1}H-NMR (600 MHz, Chloroform-*d*) δ 8.19 (s, 1H), 7.75 (s, 1H), 7.47 (dd, J = 8.5, 7.1 Hz, 1H), 7.13 (s, 1H), 7.09 (d, J = 7.0 Hz, 1H), 6.88 (d, J = 8.6 Hz, 1H), 6.67 (s, 1H), 6.58 (dd, J = 10.9, 7.1 Hz, 1H), 6.50 (t, J = 5.5 Hz, 1H), 6.04 (s, 1H), 5.55 (s, 1H), 4.91 (dd, J = 12.3, 5.4 Hz, 1H), 4.56 (d, J = 5.7 Hz, 2H), 4.52 – 4.45 (m, 2H), 3.82 (t, J = 5.0 Hz, 2H), 3.75 – 3.69 (m, 4H), 3.68 – 3.61 (m, 8H), 3.61 – 3.48 (m, 10H), 3.43 (q, J = 5.3 Hz, 2H), 3.25 (s, 2H), 2.89 – 2.69 (m, 3H), 2.44 (s, 4H), 2.15 – 2.08 (m, 1H), 1.93 – 1.86 (m, 2H), 1.80 – 1.72 (m, 3H), 1.42 (s, 4H), 0.92 (s, 6H) ppm. LRMS (ESI) *m/z* $[M+H]^+$: calculated, 1056.5; found, 1056.6.

4-((17-(4-(((6-(4-(4-((4,4-dimethylpiperidin-1-yl)methyl)-2,5-difluorophenyl)-2-oxo-1,4,9-triazaspiro[5.5]undecan-9-yl)pyrimidin-4-yl)amino)methyl)-1*H*-1,2,3-triazol-1-yl)-3,6,9,12,15-pentaoxaheptadecyl)amino)-2-(2,6-dioxopiperidin-3-yl)isoindoline-1,3-dione (ZW26711)

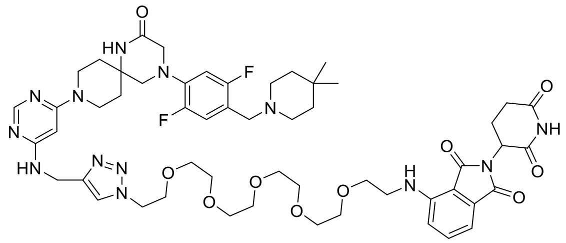

ZW26711

ZW26711 was synthesized from intermediate **6** and 4-((17-azido-3,6,9,12,15-pentaoxaheptadecyl)amino)-2-(2,6-dioxopiperidin-3-yl)isoindoline-1,3-dione using the same procedure described for ZW26677. ^{1}H-NMR (600 MHz, Chloroform-*d*) δ 8.18 (s, 1H), 7.78 (s, 1H), 7.48 (dd, J = 8.5, 7.1 Hz, 1H), 7.12 (s, 1H), 7.09 (d, J = 7.1 Hz, 1H), 6.89 (d, J = 8.5 Hz, 1H), 6.66 (s, 1H), 6.58 (dd, J = 10.8, 7.1 Hz, 1H), 6.50 (t, J = 5.5 Hz, 1H), 6.09 (s, 1H), 5.57 (s, 1H), 4.92 (dd, J = 12.3, 5.4 Hz, 1H), 4.56 (d, J = 5.8 Hz, 2H), 4.52 – 4.47 (m, 2H), 3.86 – 3.81

(m, 2H), 3.73 – 3.68 (m, 4H), 3.68 – 3.60 (m, 11H), 3.60 – 3.56 (m, 6H), 3.56 – 3.49 (m, 6H), 3.45 – 3.41 (m, 2H), 3.25 (s, 2H), 2.89 – 2.69 (m, 3H), 2.44 (s, 4H), 2.14 – 2.09 (m, 1H), 1.89 (t, *J* = 8.4 Hz, 2H), 1.80 – 1.76 (m, 2H), 1.41 (s, 4H), 0.91 (s, 6H) ppm. LRMS (ESI) *m/z* $[M+H]^+$: calculated, 1100.5; found, 1100.6.

5-((2-(2-(2-(4-(((6-(4-(4-((4,4-dimethylpiperidin-1-yl)methyl)-2,5-difluorophenyl)-2-oxo-1,4,9-triazaspiro[5.5]undecan-9-yl)pyrimidin-4-yl)amino)methyl)-1*H*-1,2,3-triazol-1-yl)ethoxy)ethoxy)ethyl)amino)-2-(2,6-dioxopiperidin-3-yl)isoindoline-1,3-dione (ZW27831)

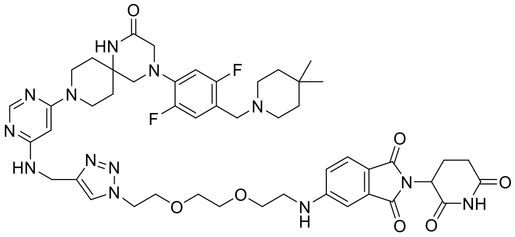

ZW27831

ZW27831 was synthesized from intermediate **6** and 5-((2-(2-(2-azidoethoxy)ethoxy)ethyl)amino)-2-(2,6-dioxopiperidin-3-yl)isoindoline-1,3-dione using the same procedure described for ZW26677. ^{1}H-NMR (600 MHz, Chloroform-*d*) δ 8.15 (s, 1H), 7.74 (s, 1H), 7.50 (d, *J* = 8.2 Hz, 1H), 7.42 (s, 1H), 7.11 (dd, *J* = 12.8, 6.5 Hz, 1H), 6.90 (d, *J* = 2.1 Hz, 1H), 6.70 (dd, *J* = 8.3, 2.1 Hz, 1H), 6.56 (dd, *J* = 10.8, 7.1 Hz, 1H), 5.64 (s, 1H), 5.43 (s, 1H), 5.27 – 5.20 (m, 1H), 4.93 (dd, *J* = 12.4, 5.4 Hz, 1H), 4.56 – 4.45 (m, 4H), 3.88 – 3.82 (m, 2H), 3.76 – 3.70 (m, 2H), 3.63 – 3.59 (m, 2H), 3.59 – 3.54 (m, 5H), 3.49 (s, 3H), 3.33 (d, *J* = 5.3 Hz, 2H), 3.26 – 3.16 (m, 2H), 2.88 – 2.69 (m, 3H), 2.42 (s, 4H), 2.14 – 2.08 (m, 1H), 1.94 (s, 2H), 1.84 (t, *J* = 5.4 Hz, 2H), 1.82 – 1.75 (m, 2H), 1.40 (t, *J* = 5.6 Hz, 4H), 0.90 (s, 6H) ppm. LRMS (ESI) *m/z* $[M+H]^+$: calculated, 968.5; found, 968.4.

5-((2-(2-(2-(2-(4-(((4-(4-(4-((4,4-dimethylpiperidin-1-yl)methyl)-2,5-difluorophenyl)-2-oxo-1,4,9-triazaspiro[5.5]undecan-9-yl)pyrimidin-2-yl)amino)methyl)-1*H*-1,2,3-triazol-1-yl)ethoxy)ethoxy)ethoxy)ethyl)amino)-2-(2,6-dioxopiperidin-3-yl)isoindoline-1,3-dione (ZW27833)

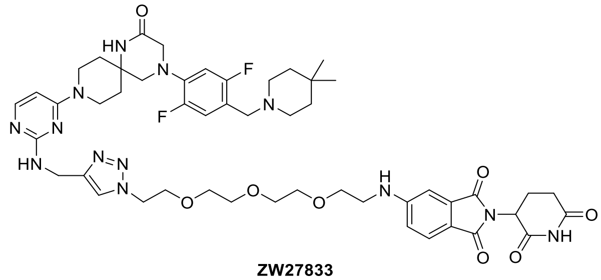

ZW27833

ZW27833 was synthesized from intermediate **6** and 5-((2-(2-(2-(2-azidoethoxy)ethoxy)ethoxy)ethyl)amino)-2-(2,6-dioxopiperidin-3-yl)isoindoline-1,3-dione using the same procedure described for ZW26677. ^{1}H-NMR (600 MHz, Chloroform-*d*) δ 8.17 (s, 1H), 7.74 (s, 1H), 7.54 (d, J = 8.3 Hz, 1H), 7.27 (s, 1H), 7.11 (dd, J = 12.9, 6.5 Hz, 1H), 6.88 (d, J = 2.1 Hz, 1H), 6.70 (dd, J = 8.4, 2.2 Hz, 1H), 6.57 (dd, J = 10.8, 7.1 Hz, 1H), 5.85 (s, 1H), 5.78 (t, J = 5.3 Hz, 1H), 5.45 (s, 1H), 4.94 (dd, J = 12.4, 5.4 Hz, 1H), 4.57 – 4.44 (m, 4H), 3.91 – 3.84 (m, 2H), 3.77 – 3.70 (m, 4H), 3.69 – 3.63 (m, 4H), 3.62 – 3.53 (m, 8H), 3.50 (s, 2H), 3.39 – 3.34 (m, 2H), 3.28 – 3.18 (m, 2H), 2.88 – 2.69 (m, 3H), 2.42 (s, 4H), 2.15 – 2.07 (m, 1H), 1.85 – 1.76 (m, 5H), 1.40 (t, J = 5.6 Hz, 4H), 0.91 (s, 6H) ppm. LRMS (ESI) *m/z* $[M+H]^+$: calculated, 1012.5; found, 1012.5.

5-((14-(4-(((6-(4-(4-((4,4-dimethylpiperidin-1-yl)methyl)-2,5-difluorophenyl)-2-oxo-1,4,9-triazaspiro[5.5]undecan-9-yl)pyrimidin-4-yl)amino)methyl)-1*H*-1,2,3-triazol-1-yl)-3,6,9,12-tetraoxatetradecyl)amino)-2-(2,6-dioxopiperidin-3-yl)isoindoline-1,3-dione (ZW27828)

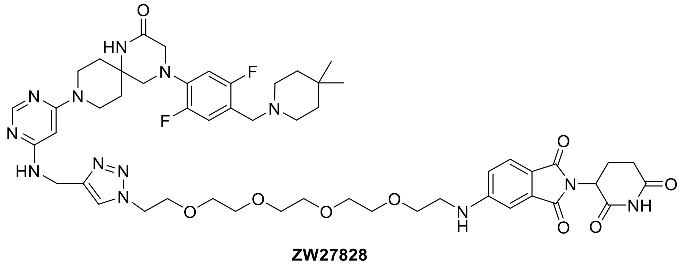

ZW27828

ZW27828 was synthesized from intermediate **6** and 5-((14-azido-3,6,9,12-tetraoxatetradecyl)amino)-2-(2,6-dioxopiperidin-3-yl)isoindoline-1,3-dione using the same procedure described for ZW26677. ^{1}H-NMR (600 MHz, Chloroform-*d*) δ 8.17 (s, 1H), 7.77 (s, 1H), 7.54 (d, J = 8.3 Hz, 1H), 7.15 (s, 1H), 7.11 (s, 1H), 6.86 (d, J = 2.2 Hz, 1H), 6.70 (dd, J = 8.3, 2.2 Hz, 1H), 6.57 (dd, J = 10.9, 7.1 Hz, 1H), 6.04 (s, 1H), 5.95 (s, 1H), 5.49 (s, 1H), 4.94 (dd, J = 12.5, 5.3 Hz, 1H), 4.50 (d, J = 5.7 Hz, 2H), 4.48 – 4.43 (m, 2H), 3.84 (t, J = 5.3 Hz, 2H), 3.75 (t, J = 5.1 Hz, 2H), 3.73 (d, J = 9.3 Hz, 2H), 3.69 – 3.63 (m, 7H), 3.62 – 3.56 (m, 8H), 3.50

(s, 2H), 3.40 – 3.35 (m, 2H), 3.28 – 3.18 (m, 2H), 2.88 – 2.70 (m, 3H), 2.42 (s, 4H), 2.14 – 2.07 (m, 1H), 1.88 – 1.82 (m, 6H), 1.40 (t, *J* = 5.5 Hz, 4H), 0.91 (s, 6H) ppm. LRMS (ESI) *m/z* $[M+H]^+$: calculated, 1056.5; found, 1056.7.

5-((17-(4-(((6-(4-(4-((4,4-dimethylpiperidin-1-yl)methyl)-2,5-difluorophenyl)-2-oxo-1,4,9-triazaspiro[5.5]undecan-9-yl)pyrimidin-4-yl)amino)methyl)-1*H*-1,2,3-triazol-1-yl)-3,6,9,12,15-pentaoxaheptadecyl)amino)-2-(2,6-dioxopiperidin-3-yl)isoindoline-1,3-dione (ZW27841)

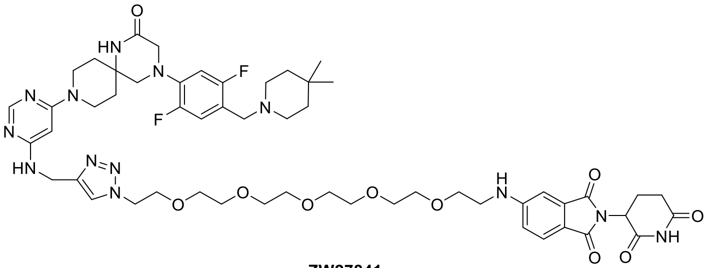

ZW27841

ZW27841 was synthesized from intermediate **6** and 5-((17-azido-3,6,9,12,15-pentaoxaheptadecyl)amino)-2-(2,6-dioxopiperidin-3-yl)isoindoline-1,3-dione using the same procedure described for ZW26677. ^{1}H-NMR (600 MHz, Chloroform-*d*) δ 8.17 (s, 1H), 7.77 (s, 1H), 7.53 (d, *J* = 8.3 Hz, 1H), 7.11 (s, 2H), 6.92 (d, *J* = 2.1 Hz, 1H), 6.74 (dd, *J* = 8.3, 2.2 Hz, 1H), 6.57 (dd, *J* = 10.8, 7.1 Hz, 1H), 6.03 (t, *J* = 5.2 Hz, 1H), 5.96 (s, 1H), 5.47 (s, 1H), 4.92 (dd, *J* = 12.3, 5.4 Hz, 1H), 4.52 (d, *J* = 5.6 Hz, 2H), 4.48 – 4.42 (m, 2H), 3.81 (t, *J* = 5.2 Hz, 2H), 3.75 – 3.70 (m, 4H), 3.65 – 3.62 (m, 10H), 3.61 – 3.58 (m, 4H), 3.57 (s, 4H), 3.49 (s, 2H), 3.39 – 3.35 (m, 2H), 3.27 – 3.19 (m, 2H), 2.89 – 2.69 (m, 3H), 2.42 (s, 4H), 2.14 – 2.08 (m, 1H), 1.96 (s, 3H), 1.89 – 1.82 (m, 3H), 1.80 – 1.75 (m, 1H), 1.40 (t, *J* = 5.6 Hz, 4H), 0.90 (s, 6H) ppm. LRMS (ESI) *m/z* $[M+H]^+$: calculated, 1100.5; found, 1100.7.

NMR spectra of degraders

(2*S*,4*R*)-1-((*S*)-2-(8-((6-(4-(4-((4,4-dimethylpiperidin-1-yl)methyl)-2,5-difluorophenyl)-2-oxo-1,4,9-triazaspiro[5.5]undecan-9-yl)pyrimidin-4-yl)amino)octanamido)-3,3-dimethylbutanoyl)-4-hydroxy-*N*-((*S*)-1-(4-(4-methylthiazol-5-yl)phenyl)ethyl)pyrrolidine-2-carboxamide (ZW27941)

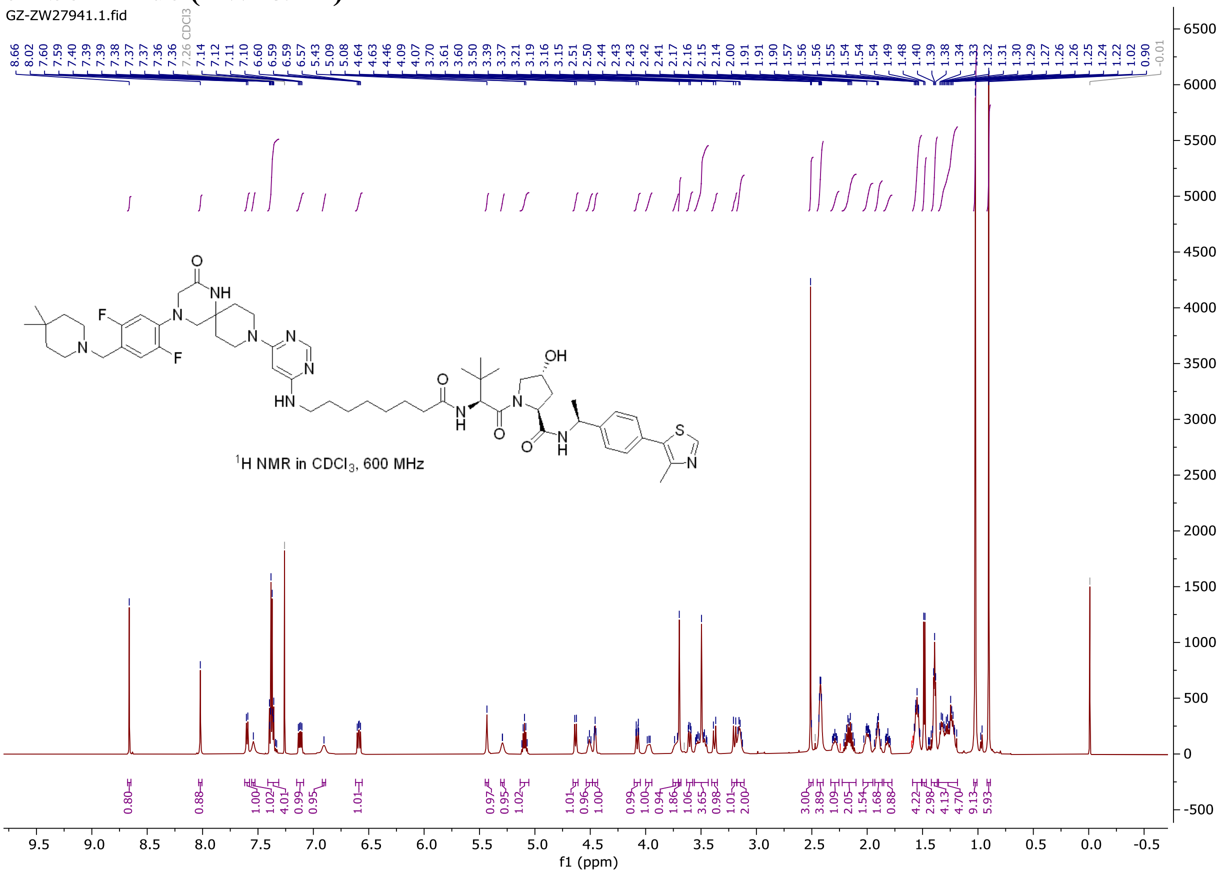

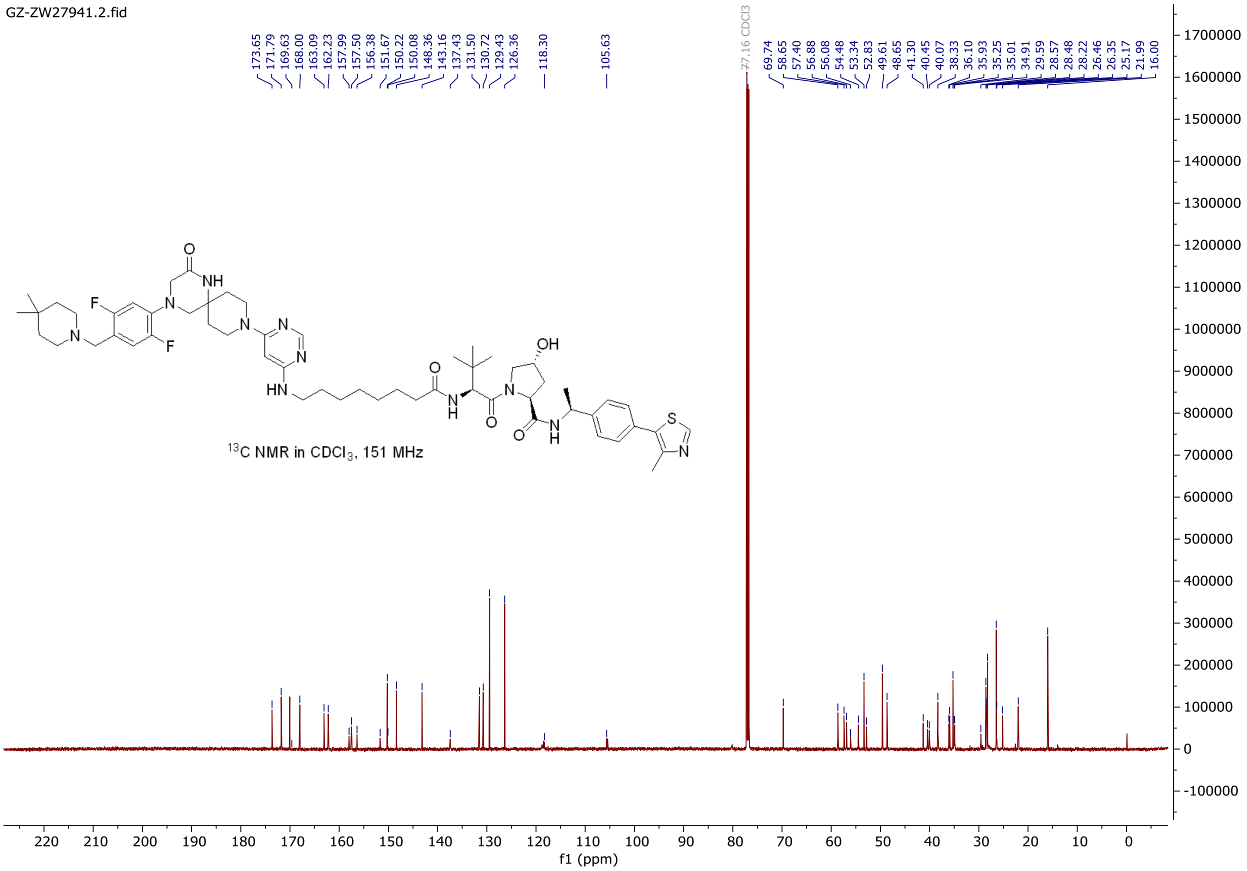

(2*S*,4*S*)-1-((*S*)-2-(8-((6-(4-(4-((4,4-dimethylpiperidin-1-yl)methyl)-2,5-difluorophenyl)-2-oxo-1,4,9-triazaspiro[5.5]undecan-9-yl)pyrimidin-4-yl)amino)octanamido)-3,3-dimethylbutanoyl)-4-hydroxy-N-(4-(4-methylthiazol-5-yl)benzyl)pyrrolidine-2-carboxamide (ZW27941NC)

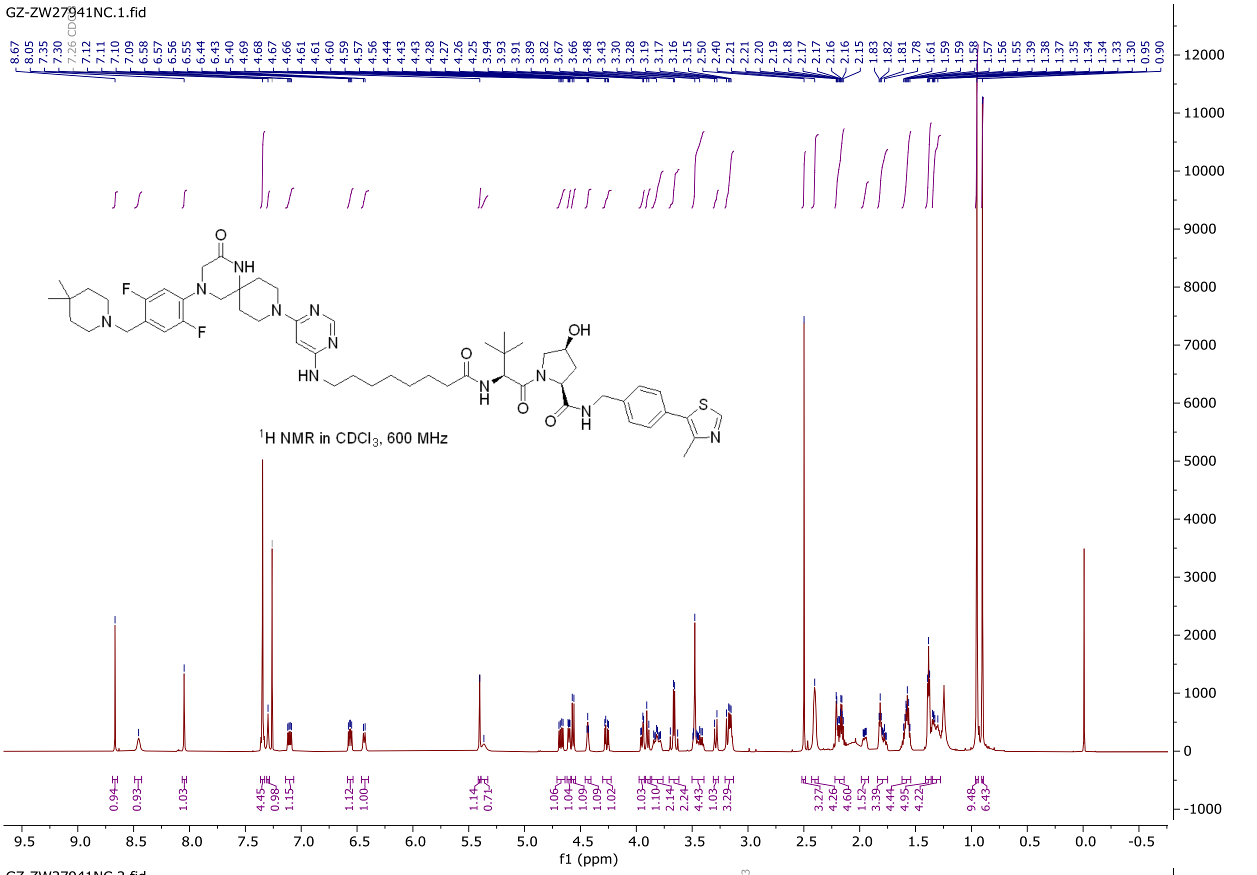
GZ-ZW27941NC.1.fid
8.67 8.05 7.35 7.30 7.26 CDCl3 7.12 7.11 7.10 7.09 6.58 6.57 6.56 6.55 6.44 6.43 5.40 4.69 4.68 4.67 4.66 4.61 4.61 4.60 4.59 4.57 4.56 4.44 4.43 4.43 4.28 4.27 4.26 4.25 3.94 3.93 3.91 3.89 3.82 3.67 3.66 3.48 3.43 3.30 3.28 3.19 3.17 3.16 3.15 2.50 2.40 2.21 2.20 2.19 2.18 2.17 2.17 2.16 2.16 2.15 1.83 1.82 1.81 1.78 1.61 1.59 1.59 1.58 1.57 1.56 1.55 1.39 1.38 1.37 1.35 1.34 1.34 1.33 1.30 0.95 0.90
0.94 0.93 1.03 4.45 0.98 1.15 1.12 1.00 1.14 0.71 1.06 1.04 1.09 1.09 1.02 1.03 1.10 2.14 2.24 4.43 1.03 3.29 3.27 4.26 4.60 1.52 3.39 4.44 4.95 4.22 9.48 6.43
^{1}H NMR in CDCl$_3$, 600 MHz
f1 (ppm)

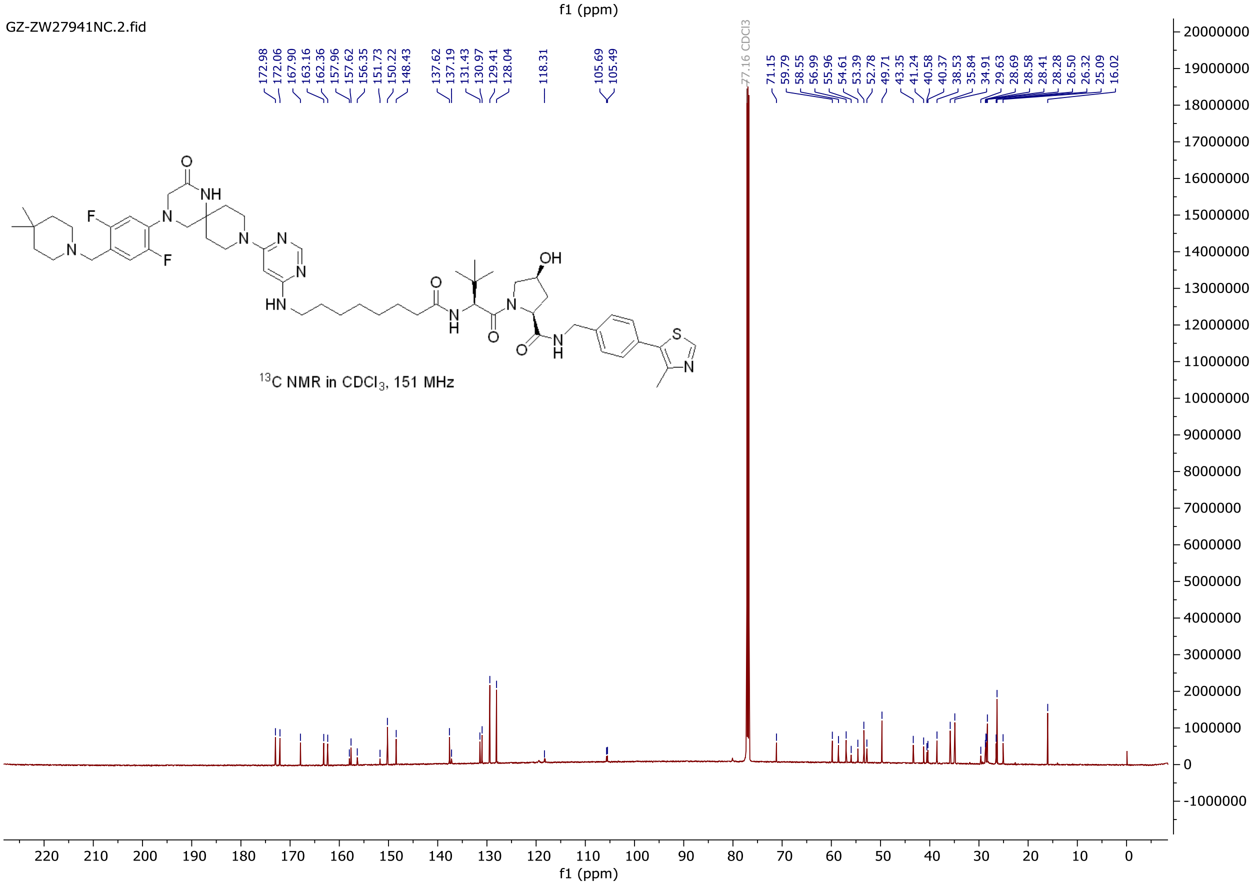
GZ-ZW27941NC.2.fid
172.98 172.06 167.90 163.16 162.36 157.96 157.62 156.35 151.73 150.22 148.43 137.62 137.19 131.43 130.97 129.41 128.04 118.31 105.69 105.49 77.16 CDCl3 71.15 59.79 58.55 56.99 55.96 54.61 53.39 52.78 49.71 43.35 41.24 40.58 40.37 38.53 35.84 34.91 29.63 28.69 28.58 28.41 28.28 26.50 26.32 25.09 16.02
^{13}C NMR in CDCl$_3$, 151 MHz
f1 (ppm)

(2*S*,4*R*)-1-((*S*)-2-(5-((6-(4-(4-((4,4-dimethylpiperidin-1-yl)methyl)-2,5-difluorophenyl)-2-oxo-1,4,9-triazaspiro[5.5]undecan-9-yl)pyrimidin-4-yl)amino)pentanamido)-3,3-dimethylbutanoyl)-4-hydroxy-*N*-((*S*)-1-(4-(4-methylthiazol-5-yl)phenyl)ethyl)pyrrolidine-2-carboxamide (ZW27938)

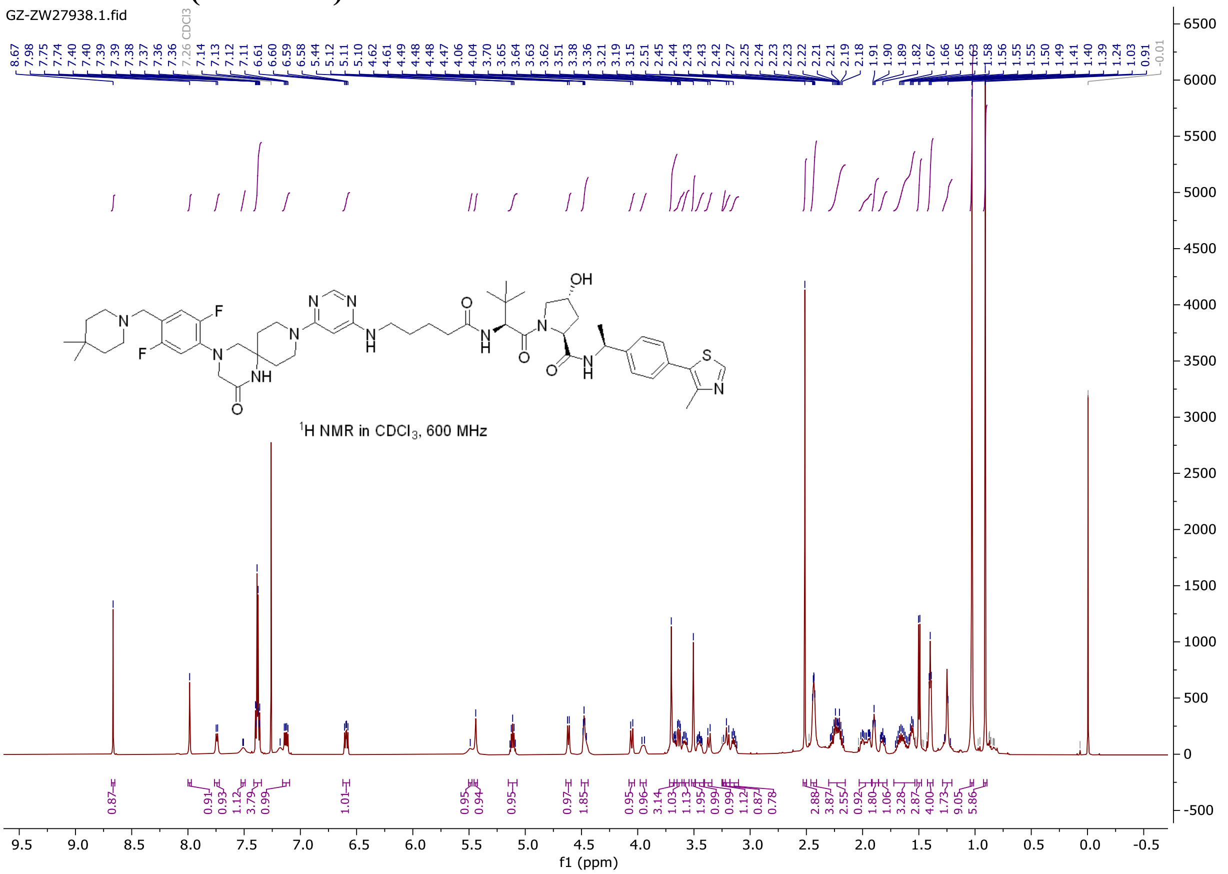

(2*S*,4*R*)-1-((*S*)-2-(6-((6-(4-(4-((4,4-dimethylpiperidin-1-yl)methyl)-2,5-difluorophenyl)-2-oxo-1,4,9-triazaspiro[5.5]undecan-9-yl)pyrimidin-4-yl)amino)hexanamido)-3,3-dimethylbutanoyl)-4-hydroxy-*N*-((*S*)-1-(4-(4-methylthiazol-5-yl)phenyl)ethyl)pyrrolidine-2-carboxamide (ZW27935)

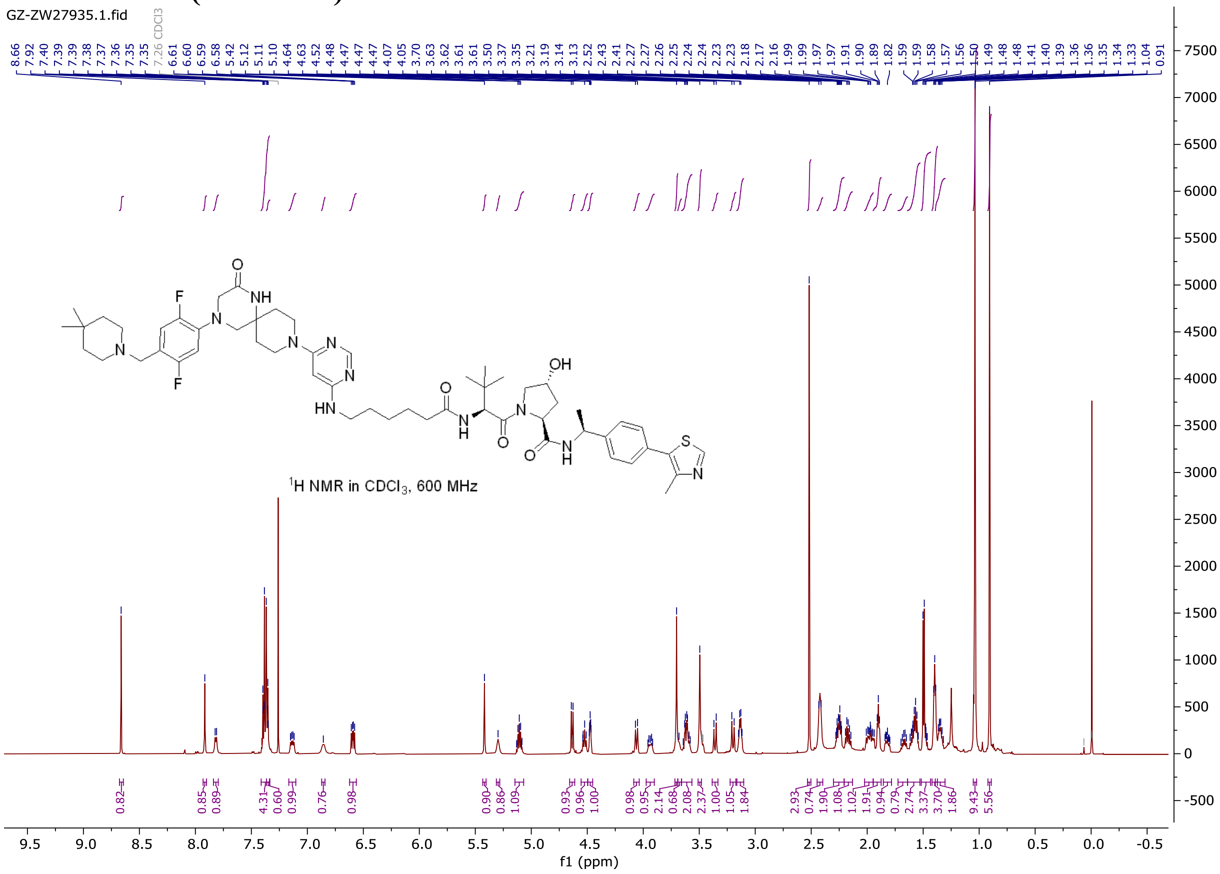

(2*S*,4*R*)-1-((S)-2-(7-((6-(4-(4-((4,4-dimethylpiperidin-1-yl)methyl)-2,5-difluorophenyl)-2-oxo-1,4,9-triazaspiro[5.5]undecan-9-yl)pyrimidin-4-yl)amino)heptanamido)-3,3-dimethylbutanoyl)-4-hydroxy-*N*-((*S*)-1-(4-(4-methylthiazol-5-yl)phenyl)ethyl)pyrrolidine-2-carboxamide (ZW27940)

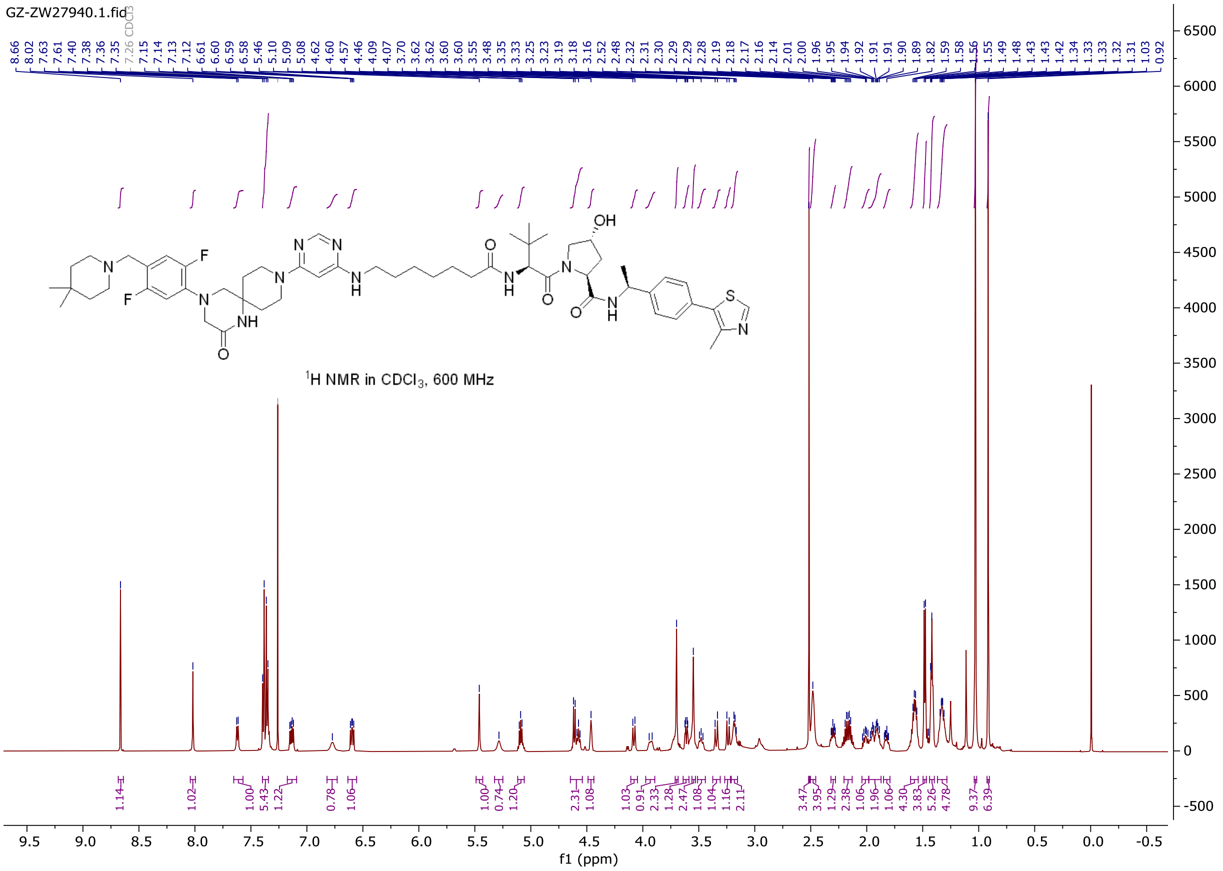

(2*S*,4*R*)-1-((*S*)-2-(9-((6-(4-(4-((4,4-dimethylpiperidin-1-yl)methyl)-2,5-difluorophenyl)-2-oxo-1,4,9-triazaspiro[5.5]undecan-9-yl)pyrimidin-4-yl)amino)nonanamido)-3,3-dimethylbutanoyl)-4-hydroxy-*N*-((*S*)-1-(4-(4-methylthiazol-5-yl)phenyl)ethyl)pyrrolidine-2-carboxamide (ZW30405)

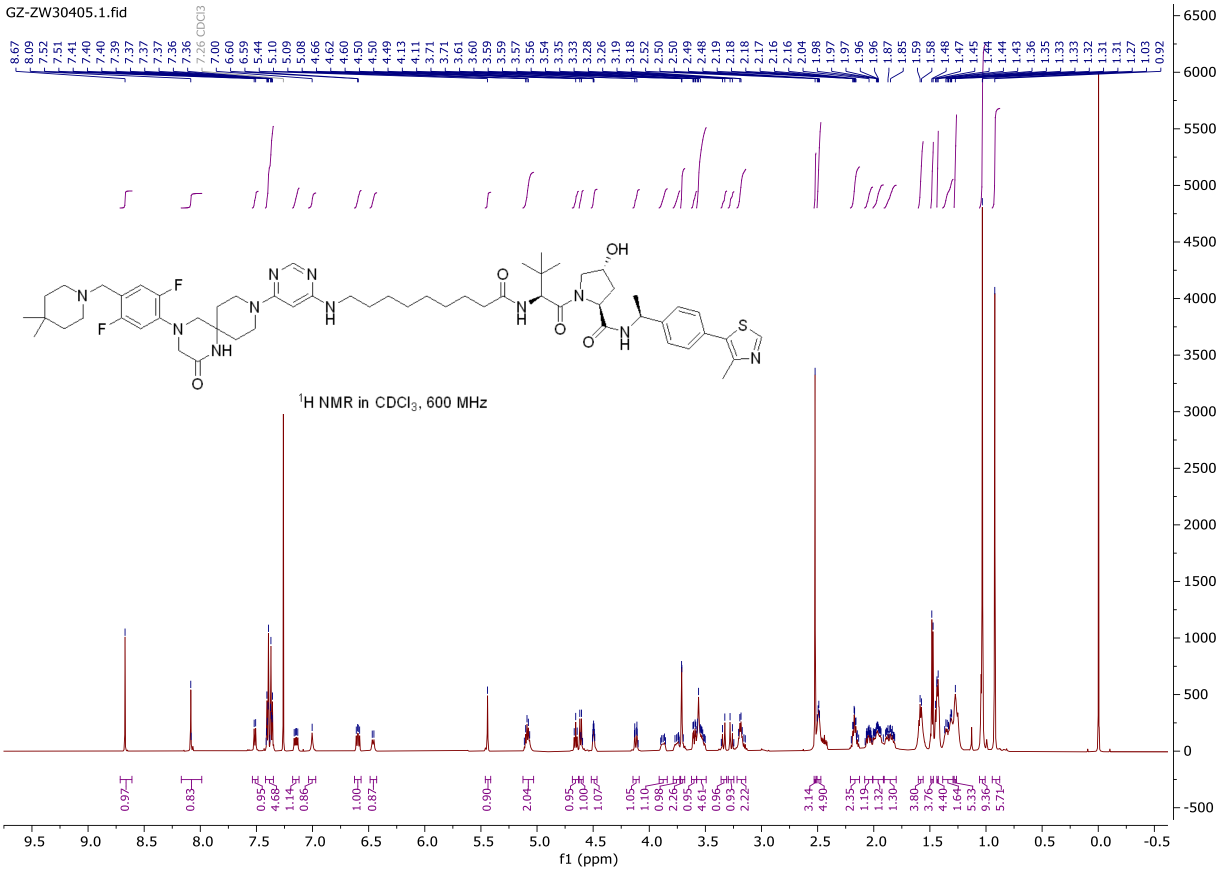

(2*S*,4*R*)-1-((*S*)-2-(10-((6-(4-(4-((4,4-dimethylpiperidin-1-yl)methyl)-2,5-difluorophenyl)-2-oxo-1,4,9-triazaspiro[5.5]undecan-9-yl)pyrimidin-4-yl)amino)decanamido)-3,3-dimethylbutanoyl)-4-hydroxy-*N*-((*S*)-1-(4-(4-methylthiazol-5-yl)phenyl)ethyl)pyrrolidine-2-carboxamide (ZW30406)

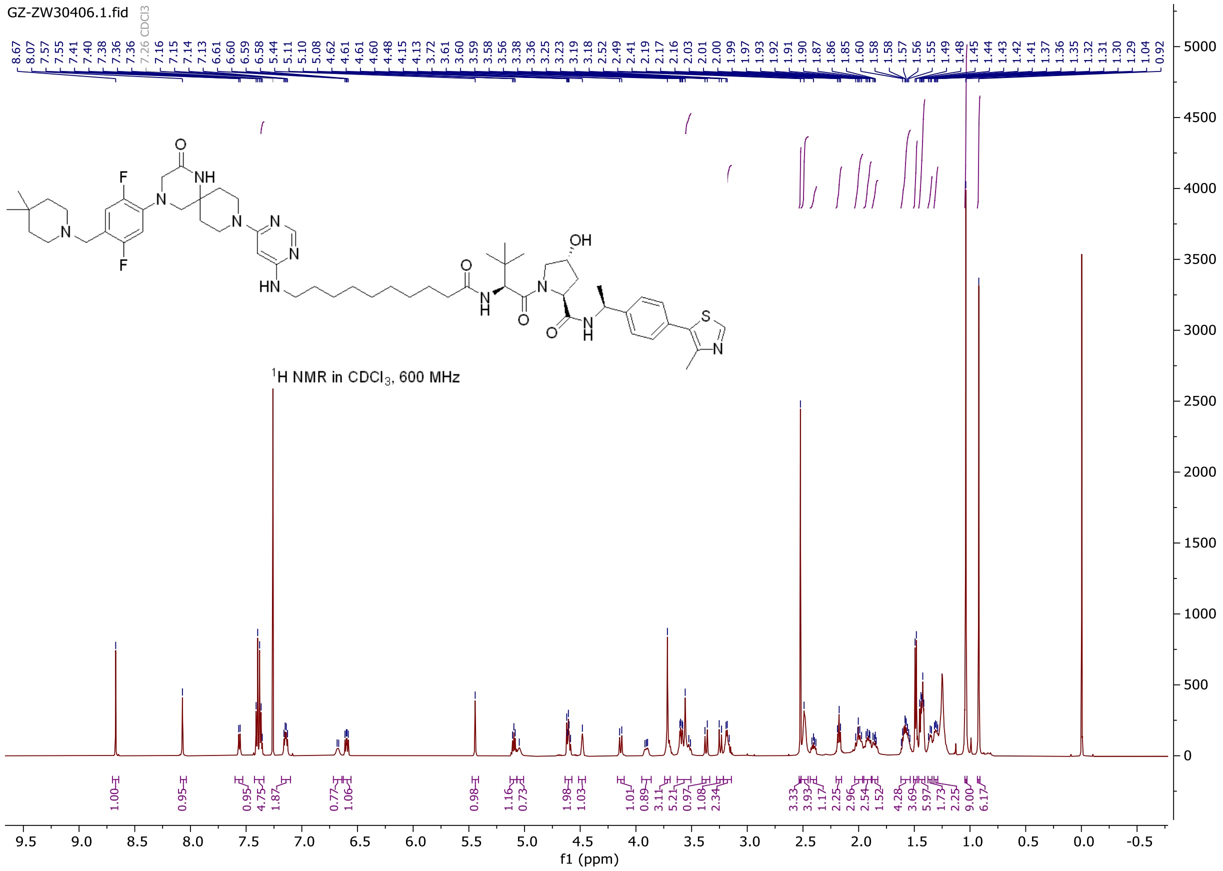

(2*S*,4*R*)-1-((*S*)-2-(11-((6-(4-(4-((4,4-dimethylpiperidin-1-yl)methyl)-2,5-difluorophenyl)-2-oxo-1,4,9-triazaspiro[5.5]undecan-9-yl)pyrimidin-4-yl)amino)undecanamido)-3,3-dimethylbutanoyl)-4-hydroxy-*N*-((*S*)-1-(4-(4-methylthiazol-5-yl)phenyl)ethyl)pyrrolidine-2-carboxamide (ZW30407)

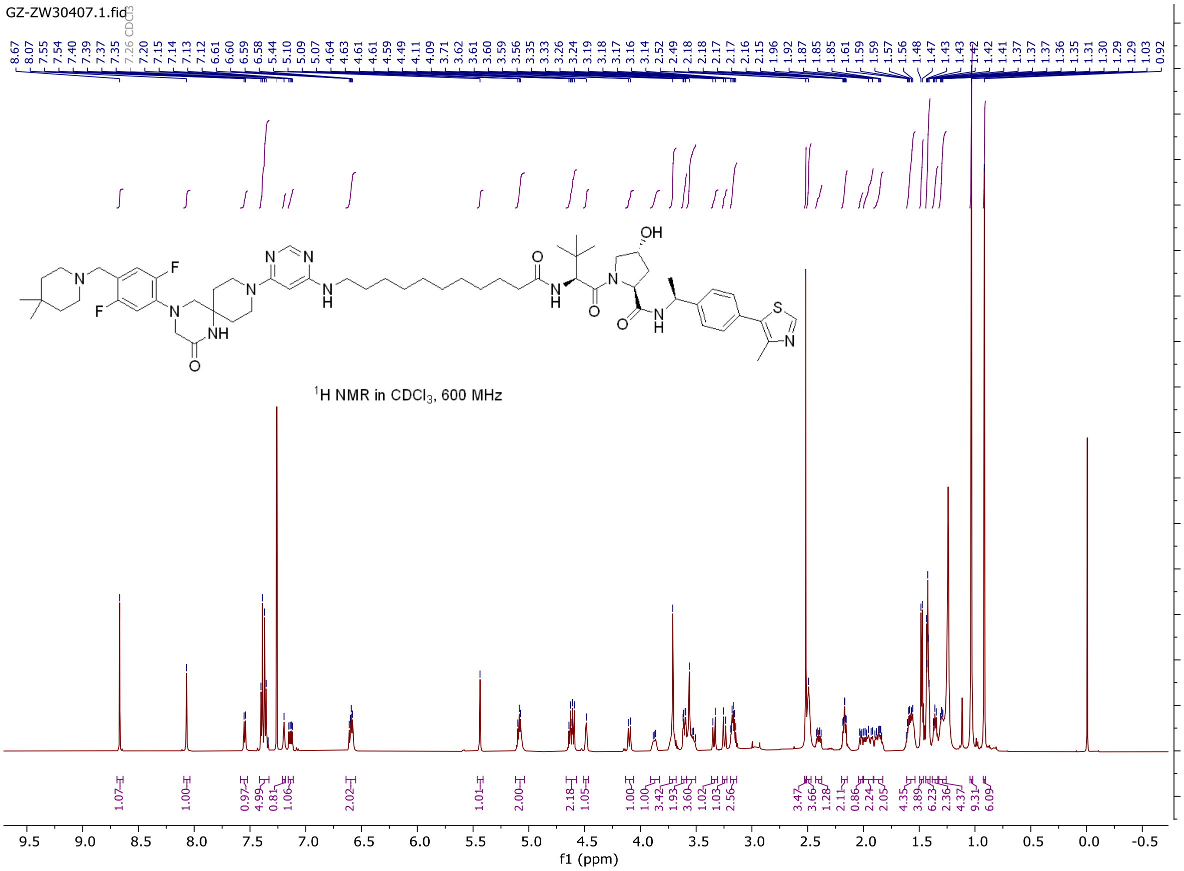

(2*S*,4*R*)-1-((*S*)-2-(12-((6-(4-(4-((4,4-dimethylpiperidin-1-yl)methyl)-2,5-difluorophenyl)-2-oxo-1,4,9-triazaspiro[5.5]undecan-9-yl)pyrimidin-4-yl)amino)dodecanamido)-3,3-dimethylbutanoyl)-4-hydroxy-*N*-((*S*)-1-(4-(4-methylthiazol-5-yl)phenyl)ethyl)pyrrolidine-2-carboxamide (ZW30408)

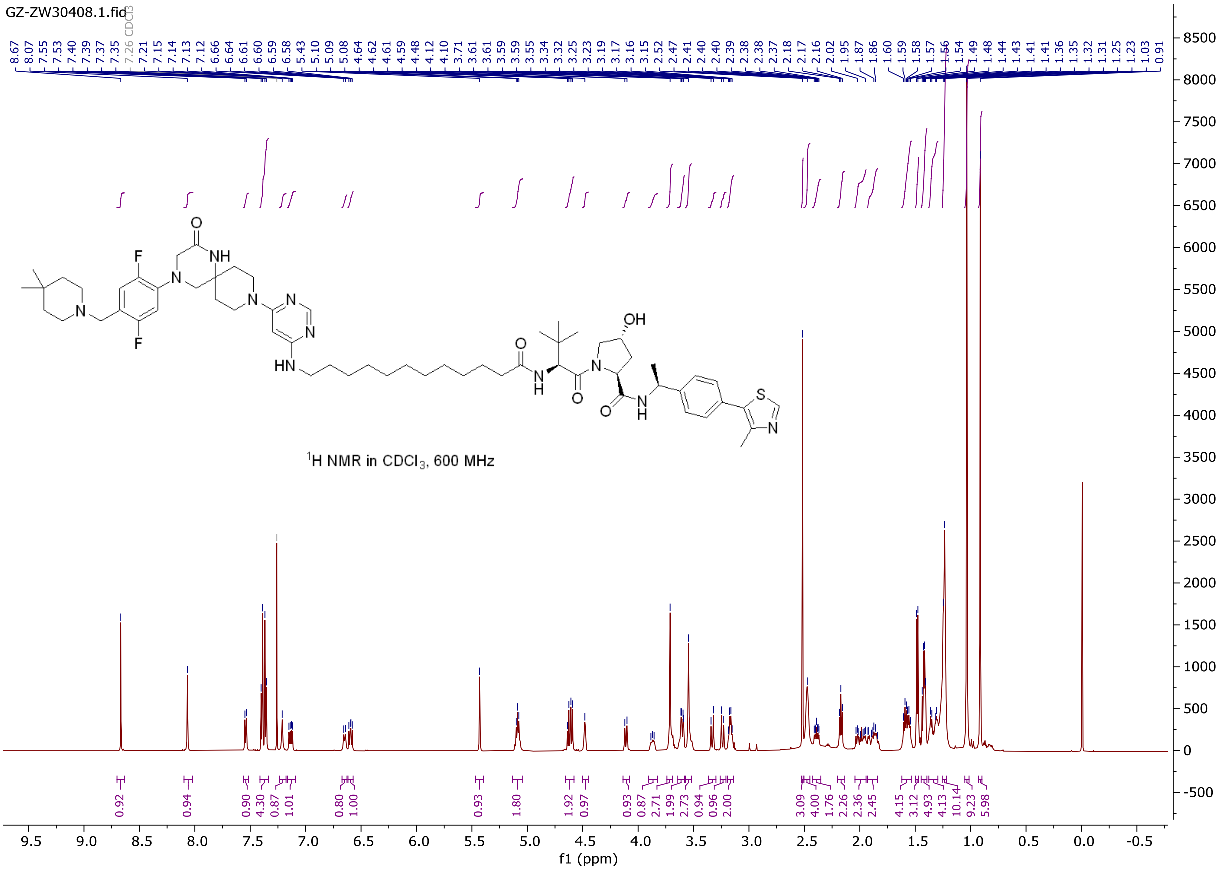

(2*S*,4*R*)-1-((*S*)-2-(4-(4-(((6-(4-(4-((4,4-dimethylpiperidin-1-yl)methyl)-2,5-difluorophenyl)-2-oxo-1,4,9-triazaspiro[5.5]undecan-9-yl)pyrimidin-4-yl)amino)methyl)-1*H*-1,2,3-triazol-1-yl)butanamido)-3,3-dimethylbutanoyl)-4-hydroxy-*N*-((*S*)-1-(4-(4-methylthiazol-5-yl)phenyl)ethyl)pyrrolidine-2-carboxamide (ZW26677)

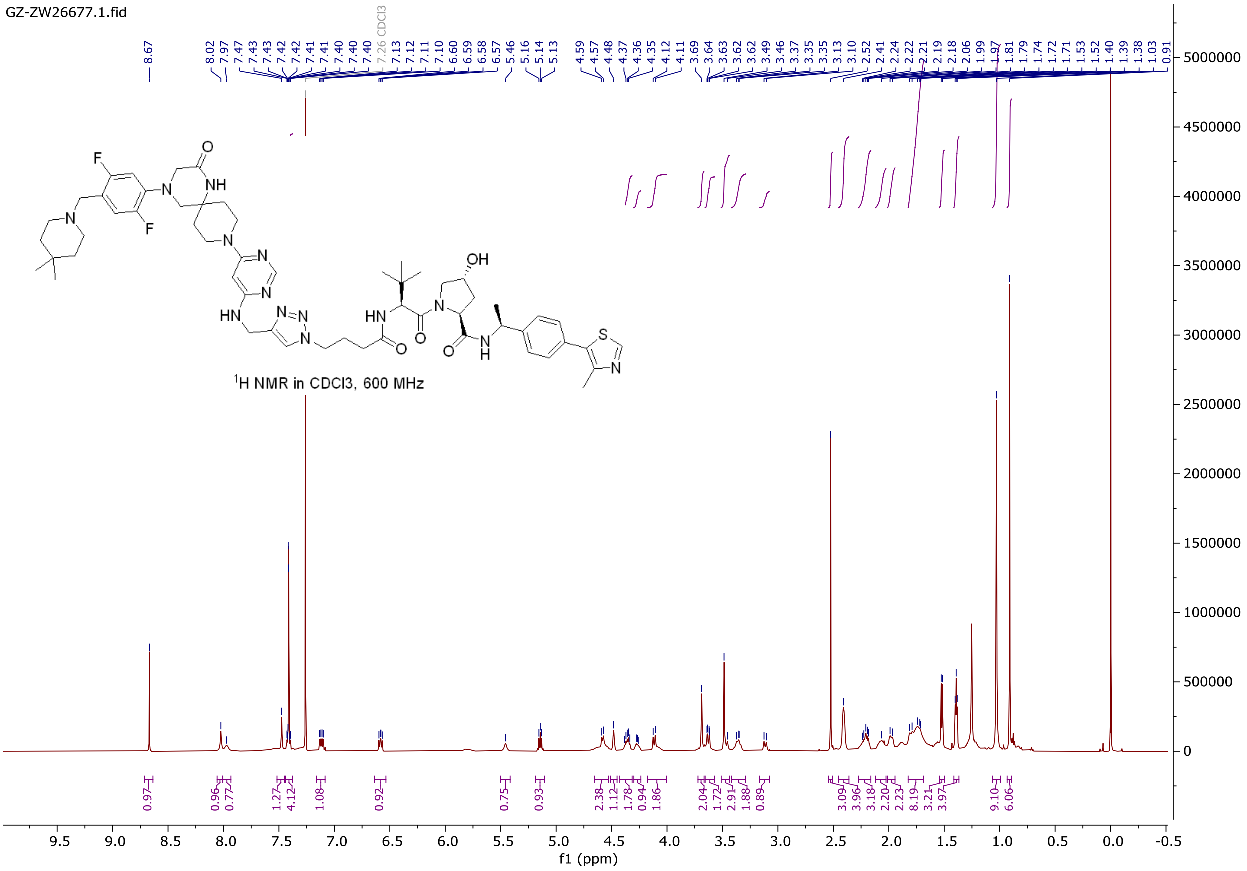

(2*S*,4*R*)-1-((*S*)-2-(6-(4-(((6-(4-(4-((4,4-dimethylpiperidin-1-yl)methyl)-2,5-difluorophenyl)-2-oxo-1,4,9-triazaspiro[5.5]undecan-9-yl)pyrimidin-4-yl)amino)methyl)-1*H*-1,2,3-triazol-1-yl)hexanamido)-3,3-dimethylbutanoyl)-4-hydroxy-*N*-((*S*)-1-(4-(4-methylthiazol-5-yl)phenyl)ethyl)pyrrolidine-2-carboxamide (ZW26704)

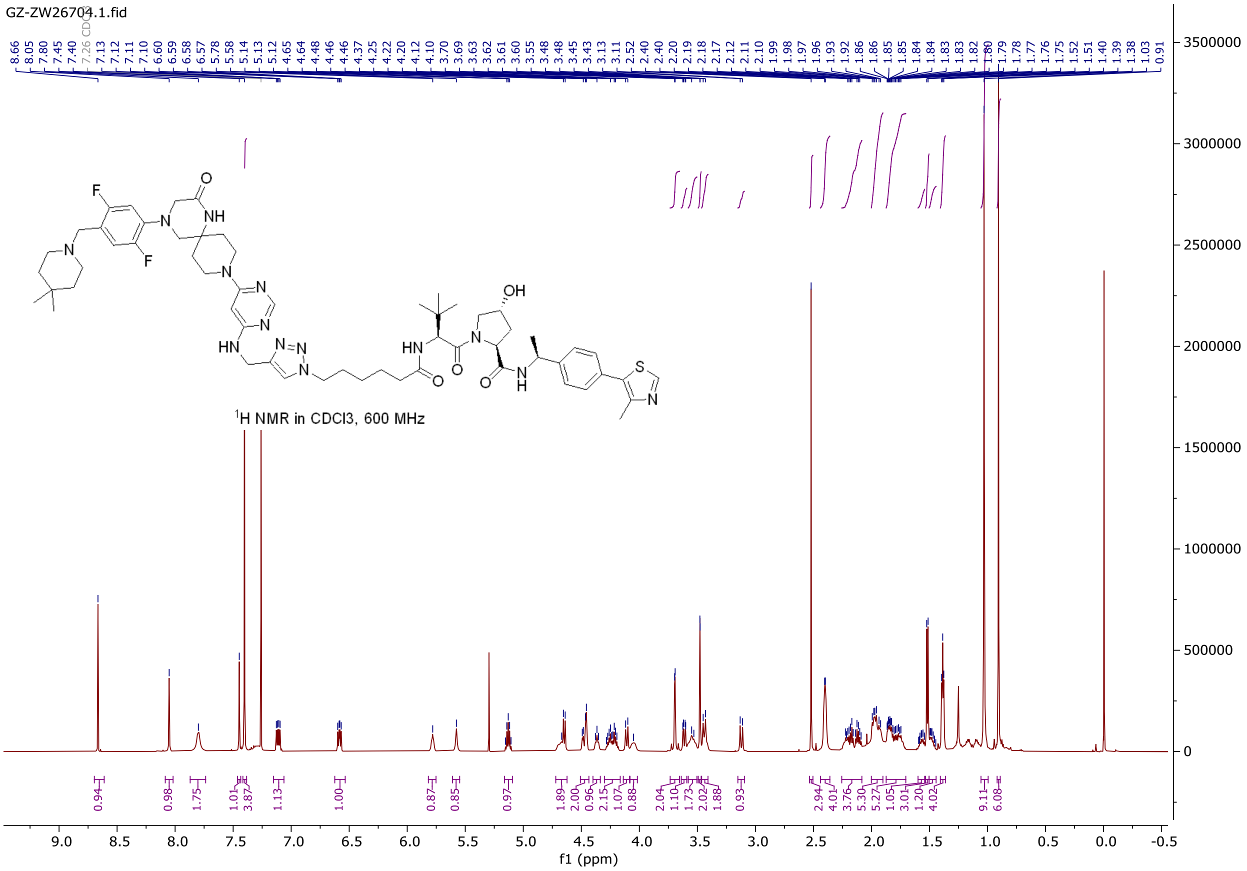

(2*S*,4*R*)-1-((*S*)-2-(8-(4-(((6-(4-(4-((4,4-dimethylpiperidin-1-yl)methyl)-2,5-difluorophenyl)-2-oxo-1,4,9-triazaspiro[5.5]undecan-9-yl)pyrimidin-4-yl)amino)methyl)-1*H*-1,2,3-triazol-1-yl)octanamido)-3,3-dimethylbutanoyl)-4-hydroxy-*N*-((*S*)-1-(4-(4-methylthiazol-5-yl)phenyl)ethyl)pyrrolidine-2-carboxamide (ZW26705)

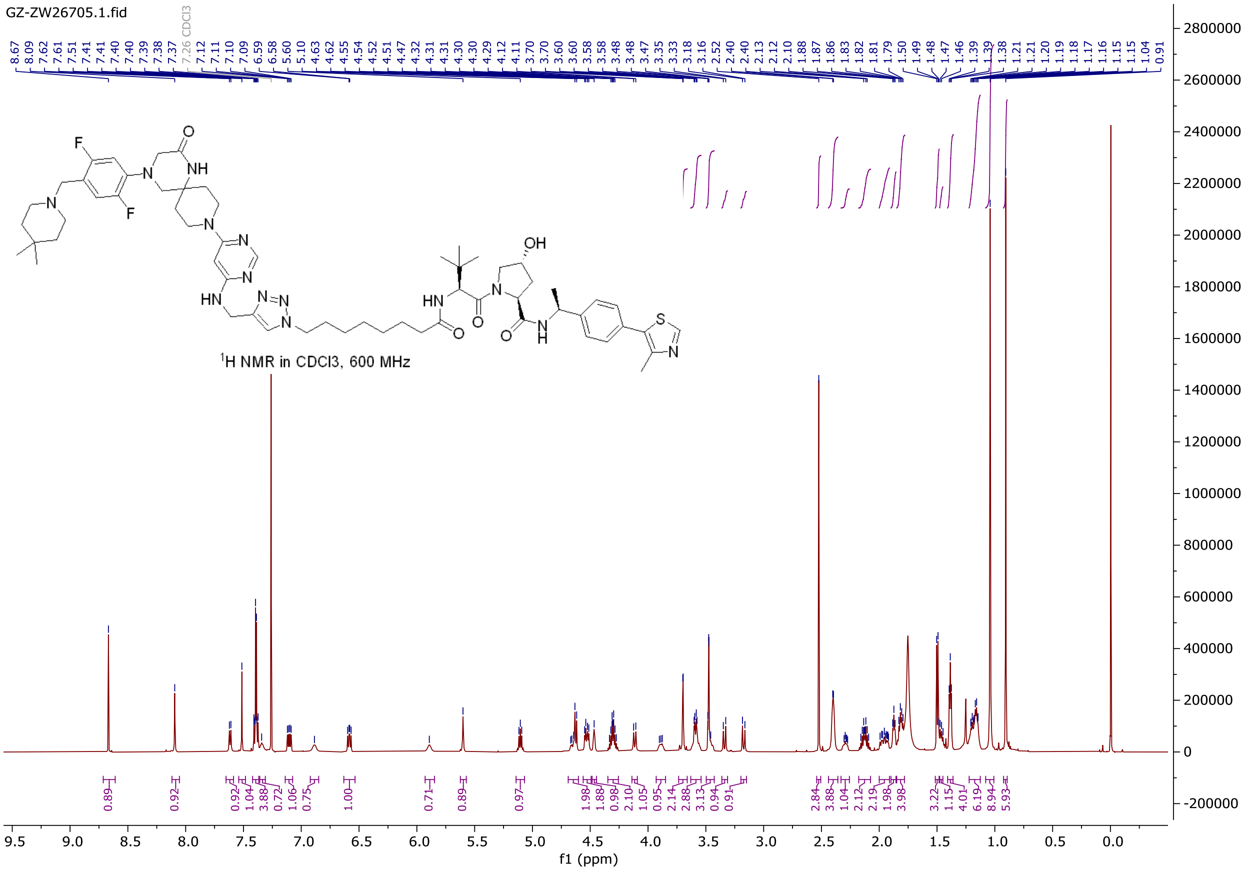

(2*S*,4*R*)-1-((*S*)-2-(10-(4-(((6-(4-(4-((4,4-dimethylpiperidin-1-yl)methyl)-2,5-difluorophenyl)-2-oxo-1,4,9-triazaspiro[5.5]undecan-9-yl)pyrimidin-4-yl)amino)methyl)-1*H*-1,2,3-triazol-1-yl)decanamido)-3,3-dimethylbutanoyl)-4-hydroxy-*N*-((*S*)-1-(4-(4-methylthiazol-5-yl)phenyl)ethyl)pyrrolidine-2-carboxamide (ZW27817)

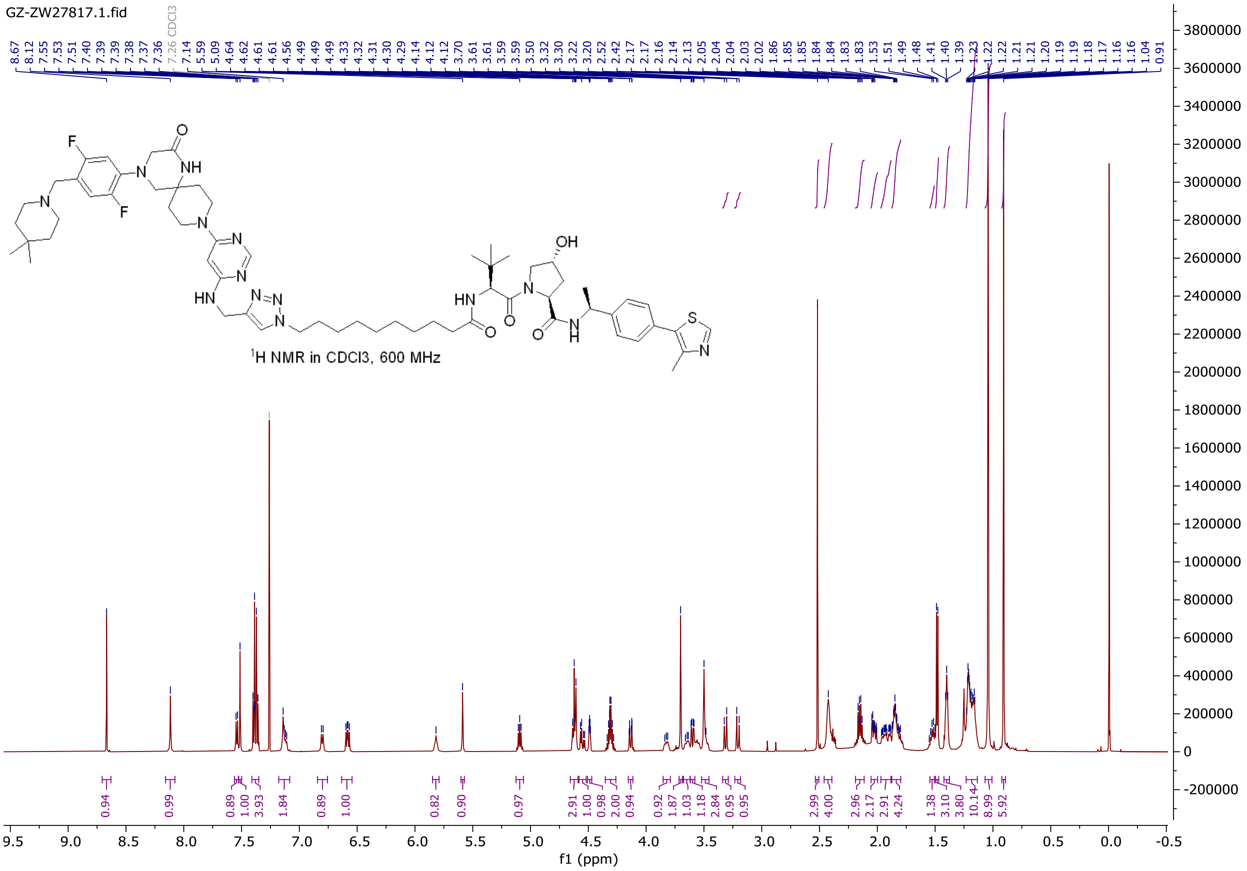

(2*S*,4*R*)-1-((*S*)-2-(2-(2-(2-(4-(((6-(4-(4-((4,4-dimethylpiperidin-1-yl)methyl)-2,5-difluorophenyl)-2-oxo-1,4,9-triazaspiro[5.5]undecan-9-yl)pyrimidin-4-yl)amino)methyl)-1*H*-1,2,3-triazol-1-yl)ethoxy)ethoxy)acetamido)-3,3-dimethylbutanoyl)-4-hydroxy-*N*-((*S*)-1-(4-(4-methylthiazol-5-yl)phenyl)ethyl)pyrrolidine-2-carboxamide (ZW27863)

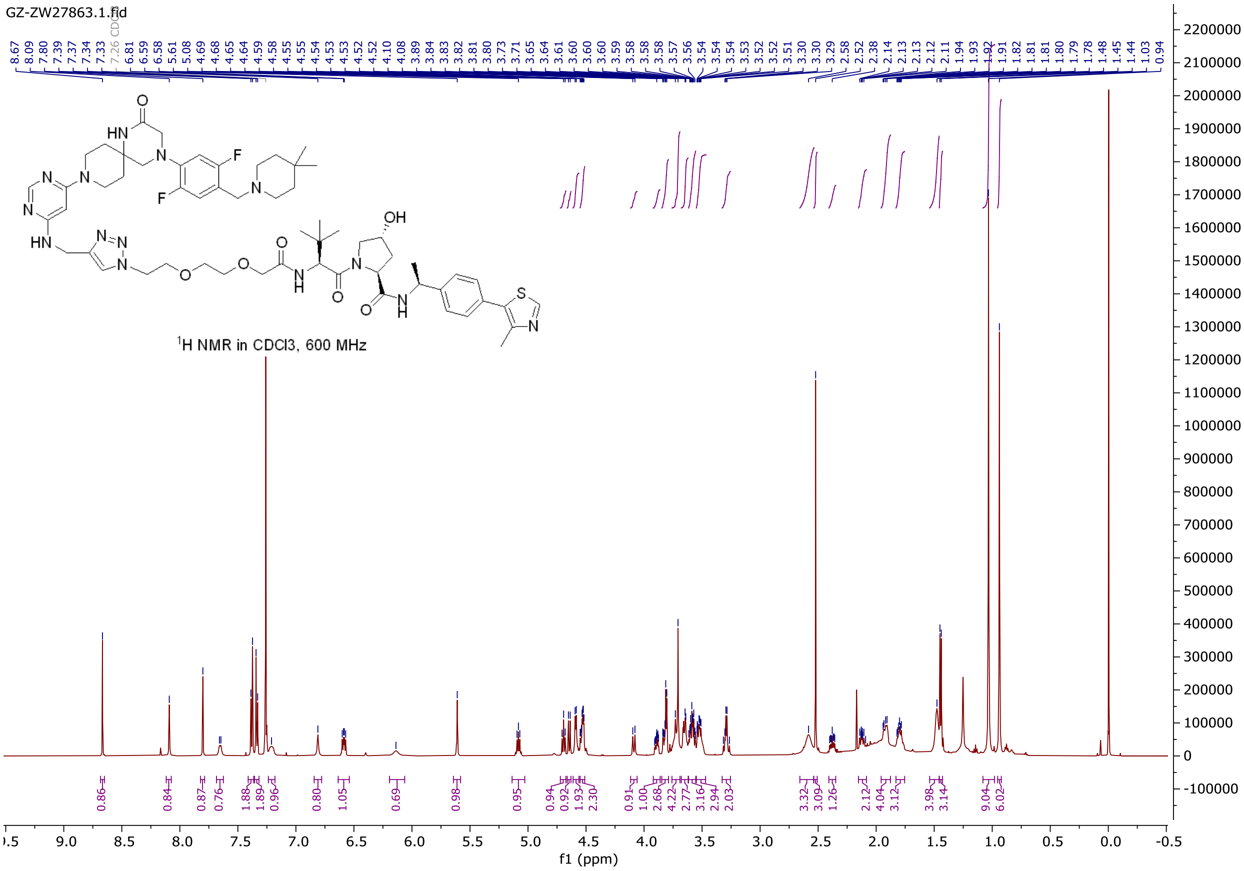

(2*S*,4*R*)-1-((*S*)-2-(tert-butyl)-14-(4-(((6-(4-(4-((4,4-dimethylpiperidin-1-yl)methyl)-2,5-difluorophenyl)-2-oxo-1,4,9-triazaspiro[5.5]undecan-9-yl)pyrimidin-4-yl)amino)methyl)-1*H*-1,2,3-triazol-1-yl)-4-oxo-6,9,12-trioxa-3-azatetradecanoyl)-4-hydroxy-*N*-((*S*)-1-(4-(4-methylthiazol-5-yl)phenyl)ethyl)pyrrolidine-2-carboxamide (ZW26718)

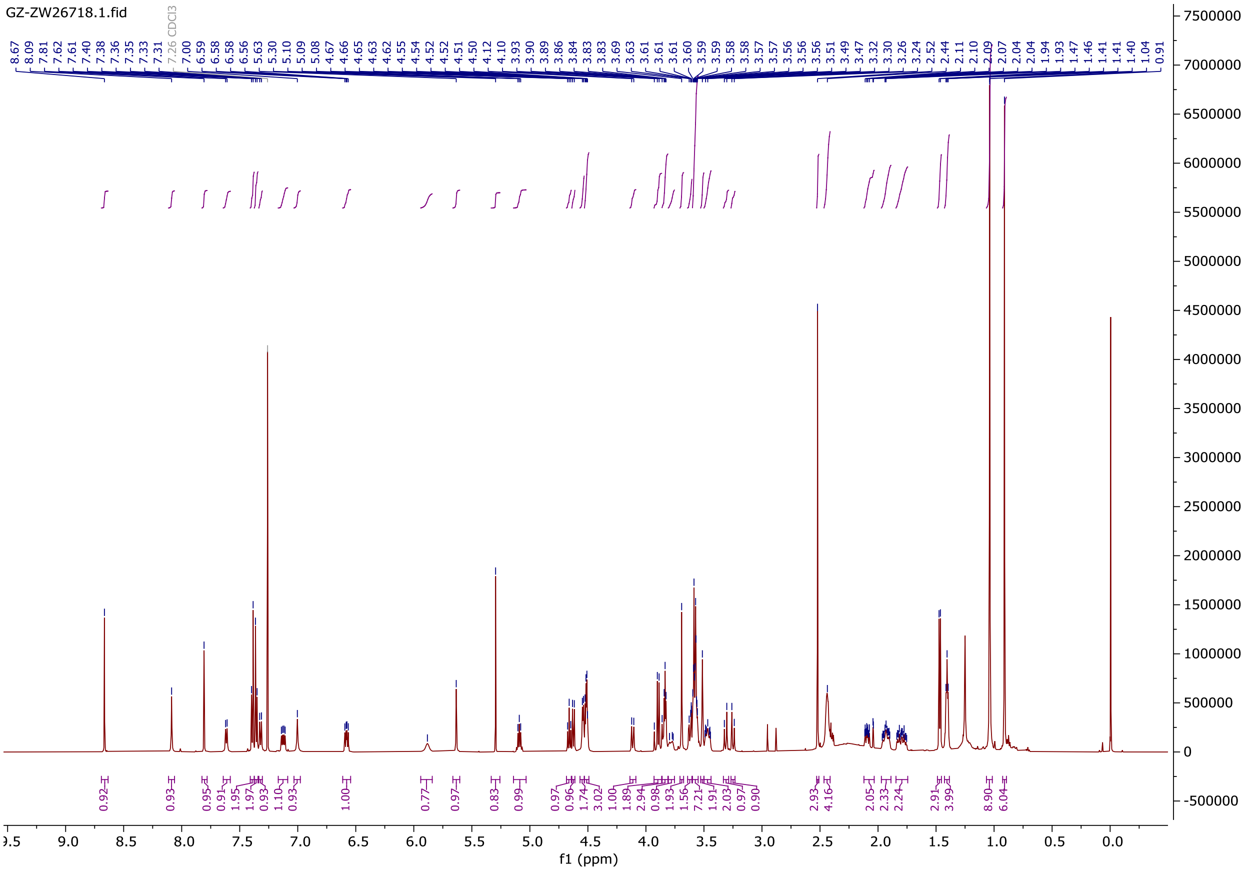

(2*S*,4*R*)-1-((*S*)-2-(tert-butyl)-17-(4-(((6-(4-(4-((4,4-dimethylpiperidin-1-yl)methyl)-2,5-difluorophenyl)-2-oxo-1,4,9-triazaspiro[5.5]undecan-9-yl)pyrimidin-4-yl)amino)methyl)-1*H*-1,2,3-triazol-1-yl)-4-oxo-6,9,12,15-tetraoxa-3-azaheptadecanoyl)-4-hydroxy-*N*-((*S*)-1-(4-(4-methylthiazol-5-yl)phenyl)ethyl)pyrrolidine-2-carboxamide (ZW27857)

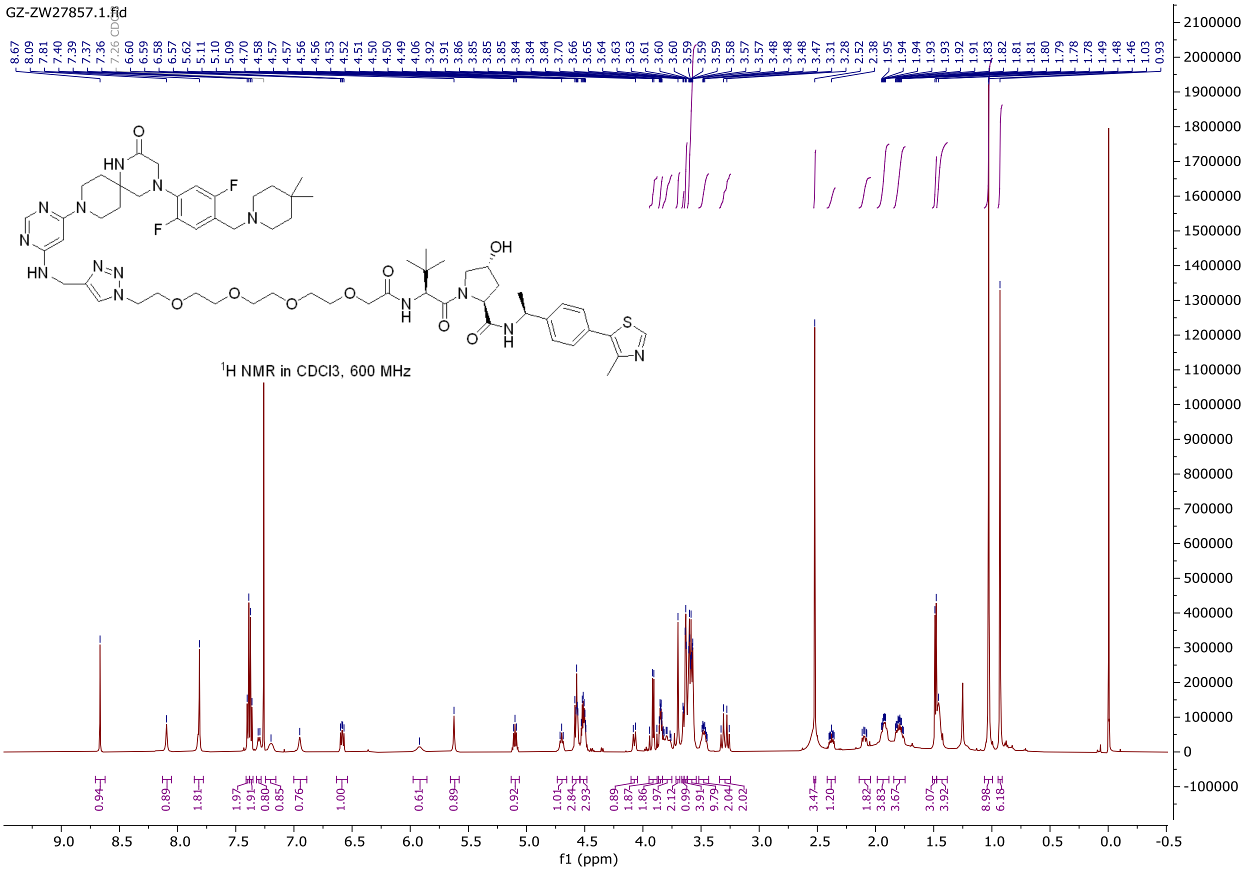

(2*S*,4*R*)-1-((*S*)-2-(tert-butyl)-20-(4-(((6-(4-(4-((4,4-dimethylpiperidin-1-yl)methyl)-2,5-difluorophenyl)-2-oxo-1,4,9-triazaspiro[5.5]undecan-9-yl)pyrimidin-4-yl)amino)methyl)-1*H*-1,2,3-triazol-1-yl)-4-oxo-6,9,12,15,18-pentaoxa-3-azaicosanoyl)-4-hydroxy-*N*-((*S*)-1-(4-(4-methylthiazol-5-yl)phenyl)ethyl)pyrrolidine-2-carboxamide (ZW27847)

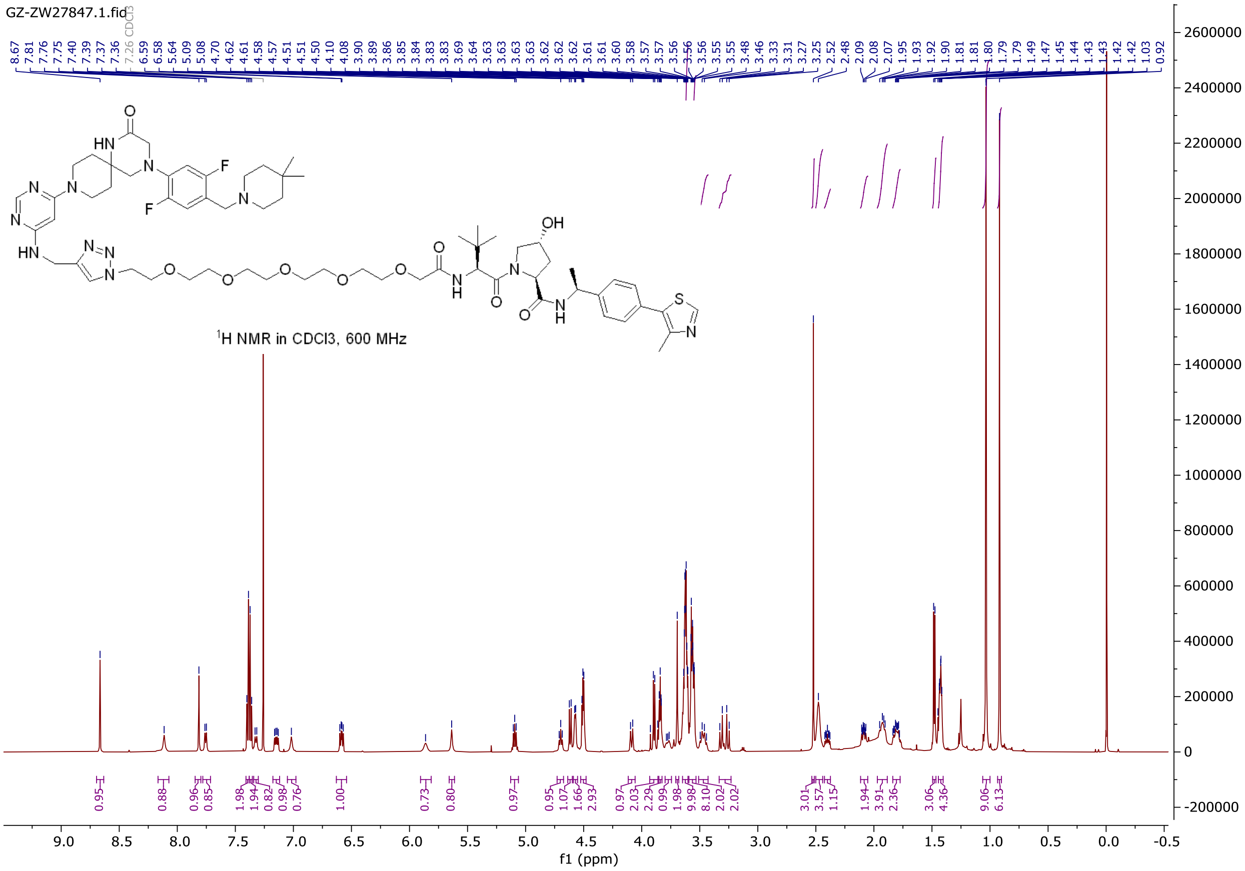

4-((4-(4-(((6-(4-(4-((4,4-dimethylpiperidin-1-yl)methyl)-2,5-difluorophenyl)-2-oxo-1,4,9-triazaspiro[5.5]undecan-9-yl)pyrimidin-4-yl)amino)methyl)-1*H*-1,2,3-triazol-1-yl)butyl)amino)-2-(2,6-dioxopiperidin-3-yl)isoindoline-1,3-dione (ZW26725)

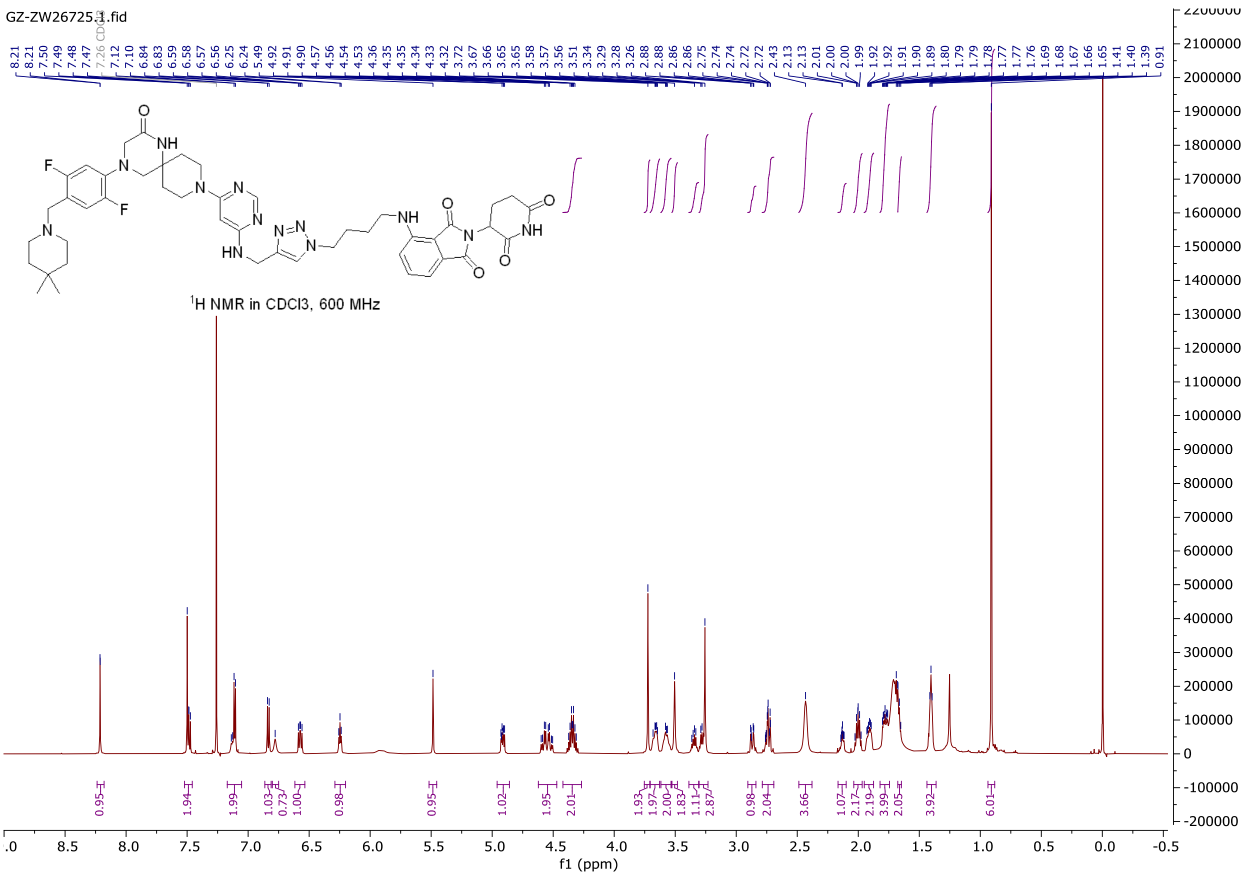

4-((6-(4-(((6-(4-(4-((4,4-dimethylpiperidin-1-yl)methyl)-2,5-difluorophenyl)-2-oxo-1,4,9-triazaspiro[5.5]undecan-9-yl)pyrimidin-4-yl)amino)methyl)-1*H*-1,2,3-triazol-1-yl)hexyl)amino)-2-(2,6-dioxopiperidin-3-yl)isoindoline-1,3-dione (ZW26715)

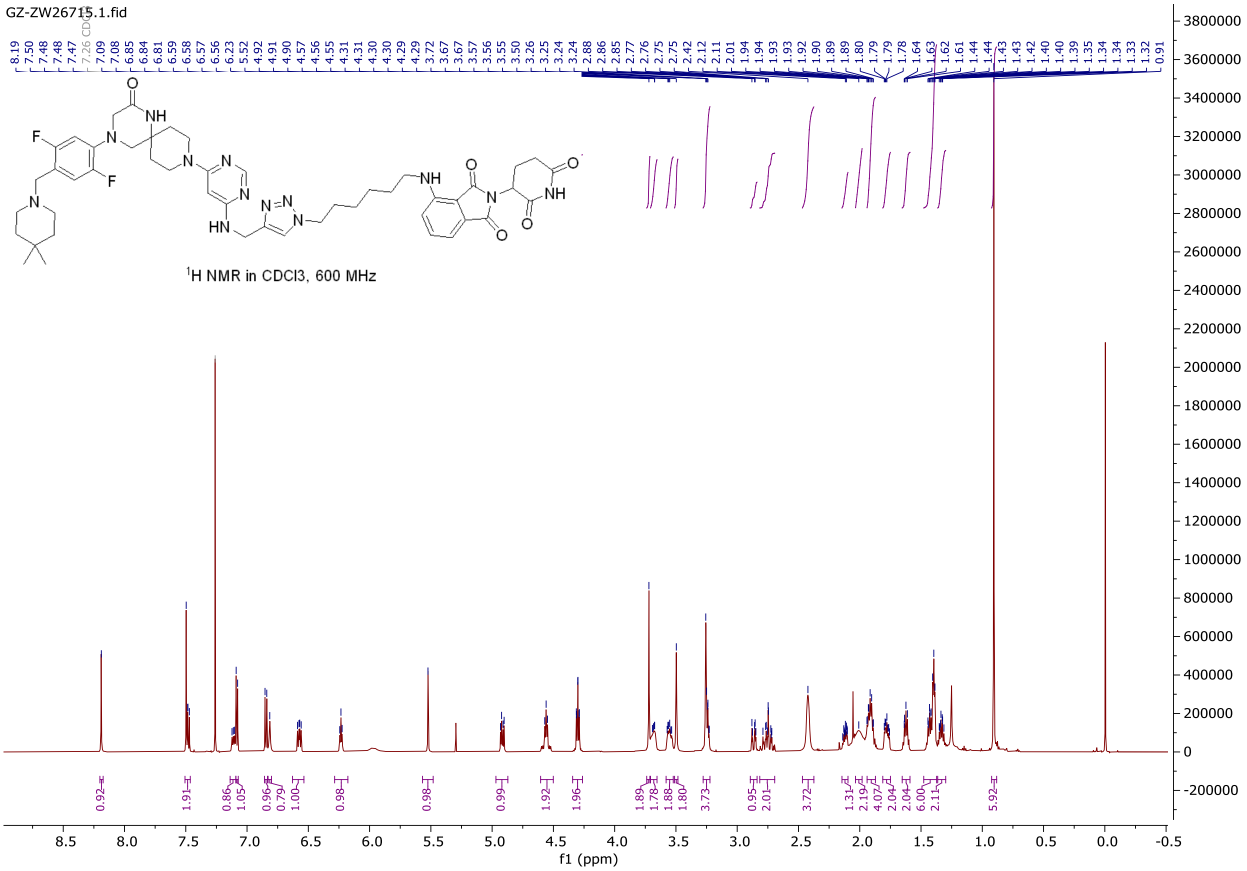

4-((8-(4-(((6-(4-(4-((4,4-dimethylpiperidin-1-yl)methyl)-2,5-difluorophenyl)-2-oxo-1,4,9-triazaspiro[5.5]undecan-9-yl)pyrimidin-4-yl)amino)methyl)-1*H*-1,2,3-triazol-1-yl)octyl)amino)-2-(2,6-dioxopiperidin-3-yl)isoindoline-1,3-dione (ZW26726)

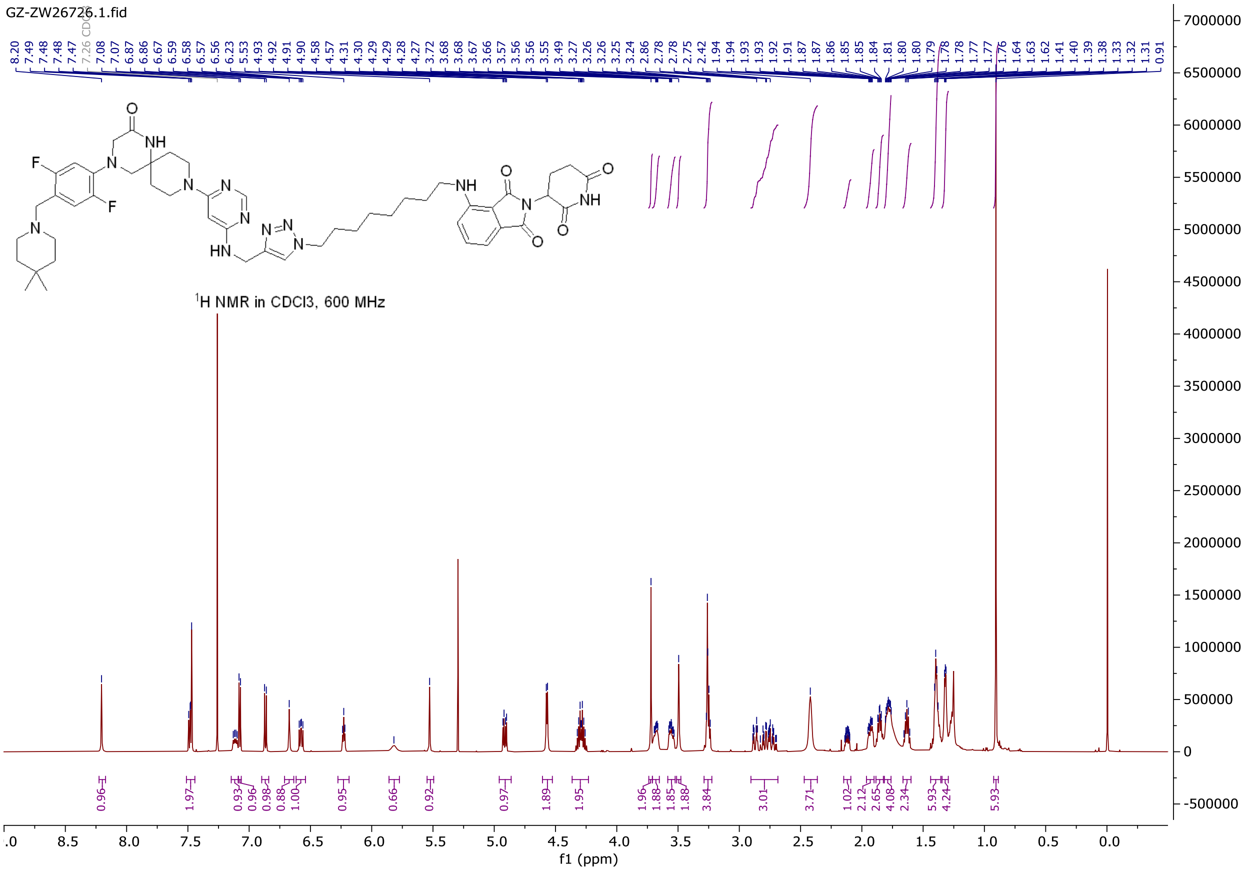

4-((10-(4-(((6-(4-(4-((4,4-dimethylpiperidin-1-yl)methyl)-2,5-difluorophenyl)-2-oxo-1,4,9-triazaspiro[5.5]undecan-9-yl)pyrimidin-4-yl)amino)methyl)-1*H*-1,2,3-triazol-1-yl)decyl)amino)-2-(2,6-dioxopiperidin-3-yl)isoindoline-1,3-dione (ZW26746)

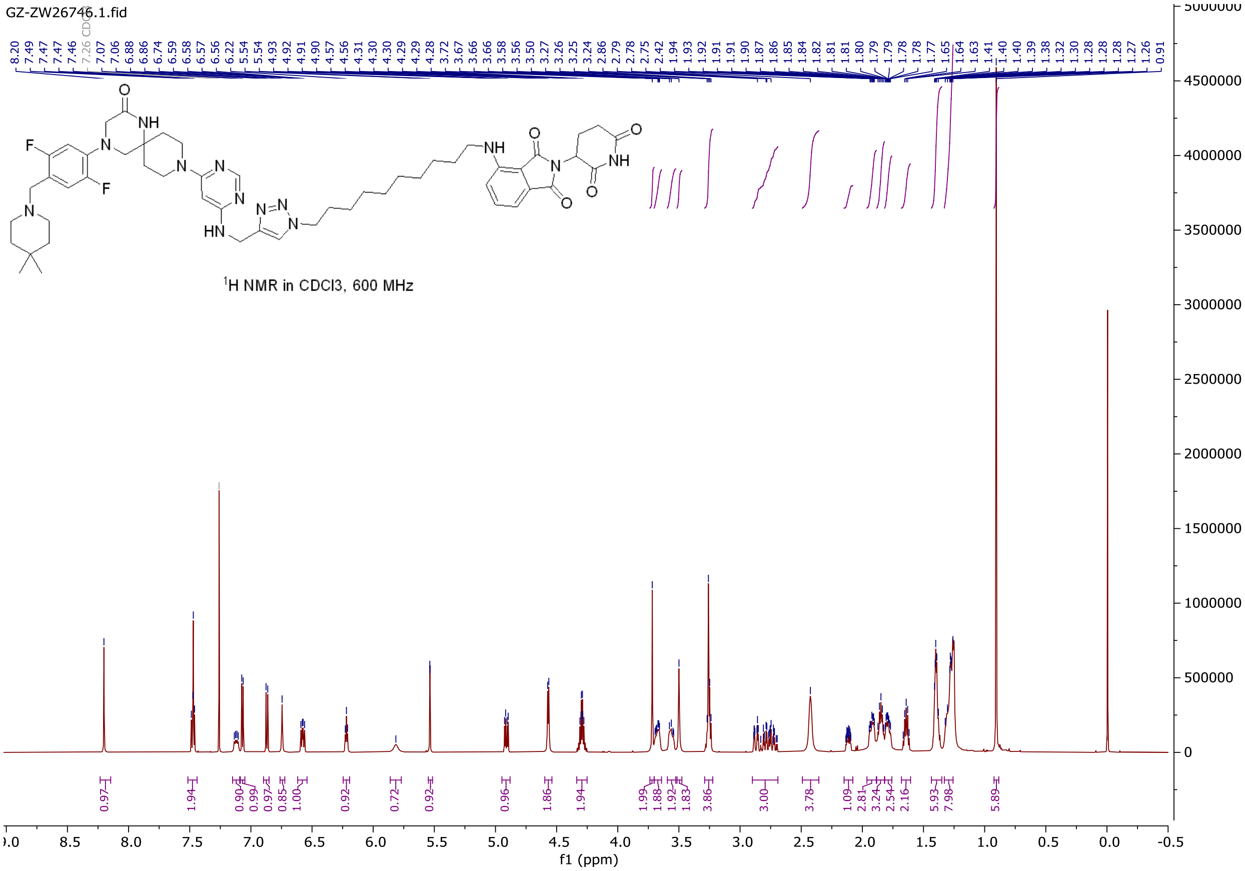

4-((2-(2-(2-(4-(((6-(4-(4-((4,4-dimethylpiperidin-1-yl)methyl)-2,5-difluorophenyl)-2-oxo-1,4,9-triazaspiro[5.5]undecan-9-yl)pyrimidin-4-yl)amino)methyl)-1*H*-1,2,3-triazol-1-yl)ethoxy)ethoxy)ethyl)amino)-2-(2,6-dioxopiperidin-3-yl)isoindoline-1,3-dione (ZW27825)

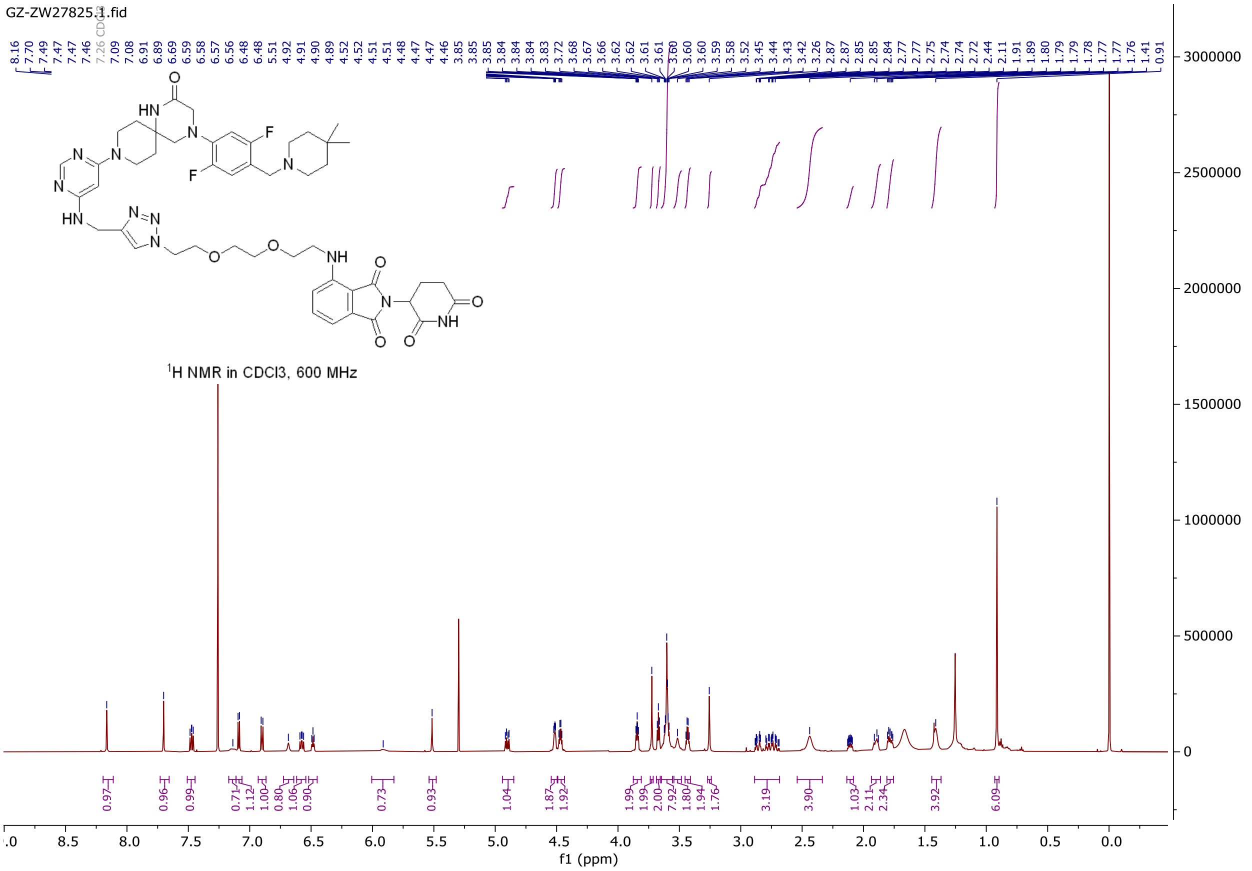

4-((2-(2-(2-(2-(4-(((6-(4-(4-((4,4-dimethylpiperidin-1-yl)methyl)-2,5-difluorophenyl)-2-oxo-1,4,9-triazaspiro[5.5]undecan-9-yl)pyrimidin-4-yl)amino)methyl)-1*H*-1,2,3-triazol-1-yl)ethoxy)ethoxy)ethoxy)ethyl)amino)-2-(2,6-dioxopiperidin-3-yl)isoindoline-1,3-dione (ZW26710)

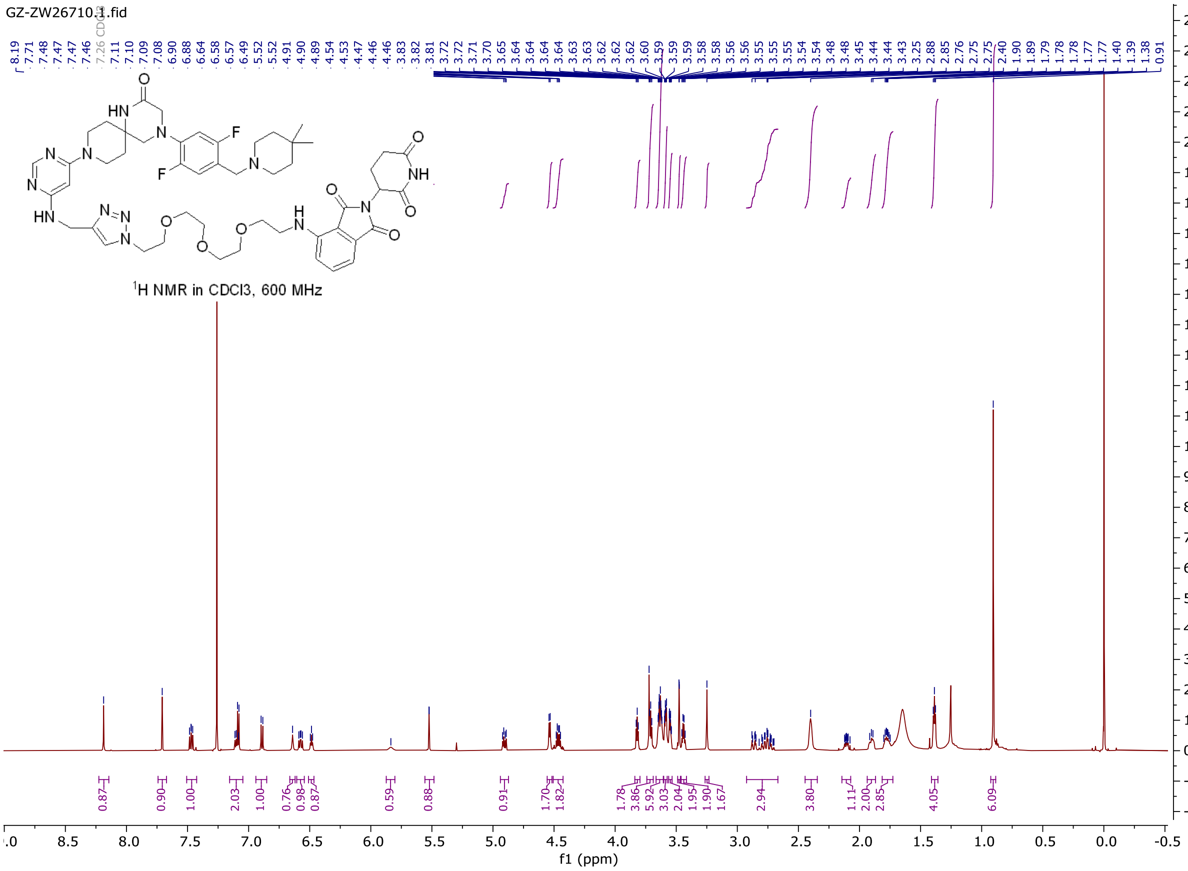

4-((14-(4-(((6-(4-(4-((4,4-dimethylpiperidin-1-yl)methyl)-2,5-difluorophenyl)-2-oxo-1,4,9-triazaspiro[5.5]undecan-9-yl)pyrimidin-4-yl)amino)methyl)-1*H*-1,2,3-triazol-1-yl)-3,6,9,12-tetraoxatetradecyl)amino)-2-(2,6-dioxopiperidin-3-yl)isoindoline-1,3-dione (ZW27818)

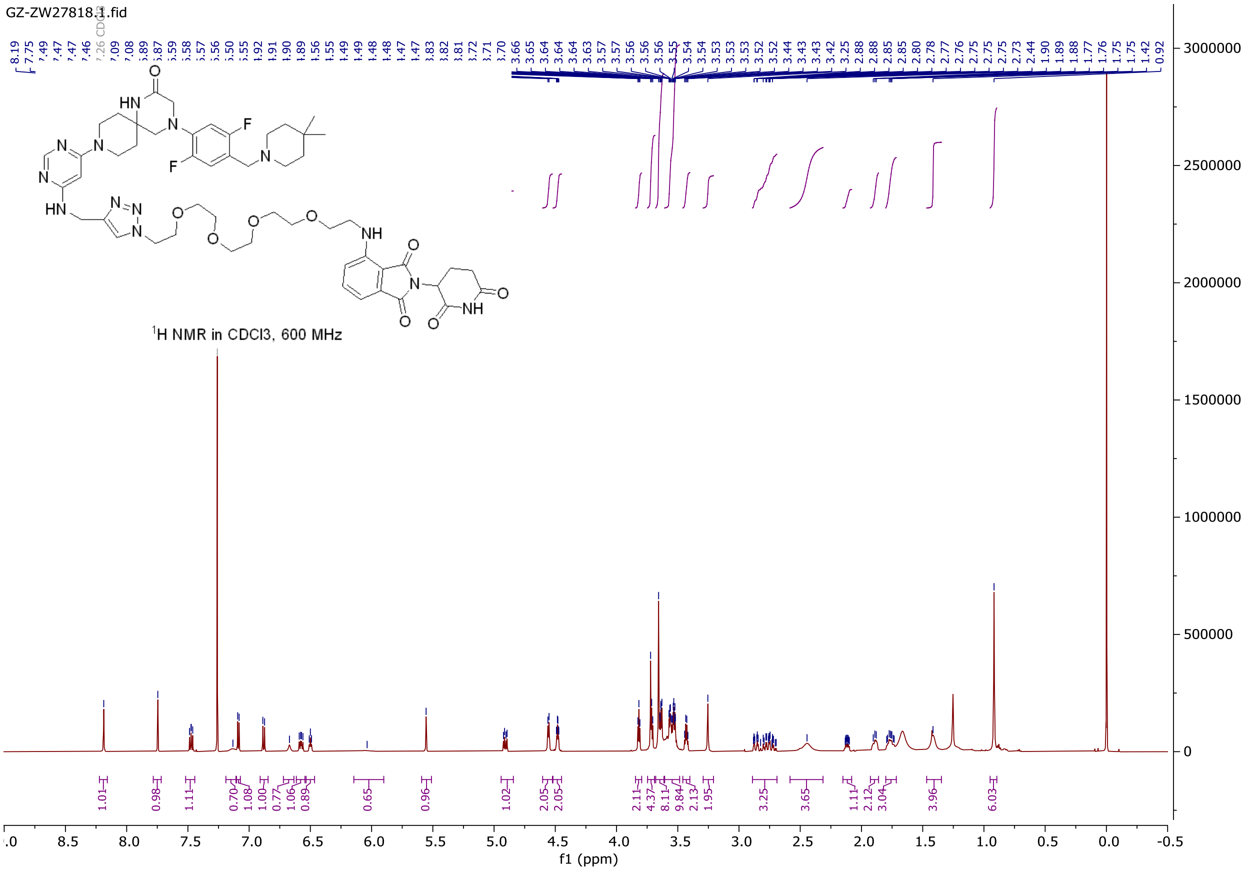

4-((17-(4-(((6-(4-(4-((4,4-dimethylpiperidin-1-yl)methyl)-2,5-difluorophenyl)-2-oxo-1,4,9-triazaspiro[5.5]undecan-9-yl)pyrimidin-4-yl)amino)methyl)-1*H*-1,2,3-triazol-1-yl)-3,6,9,12,15-pentaoxaheptadecyl)amino)-2-(2,6-dioxopiperidin-3-yl)isoindoline-1,3-dione (ZW26711)

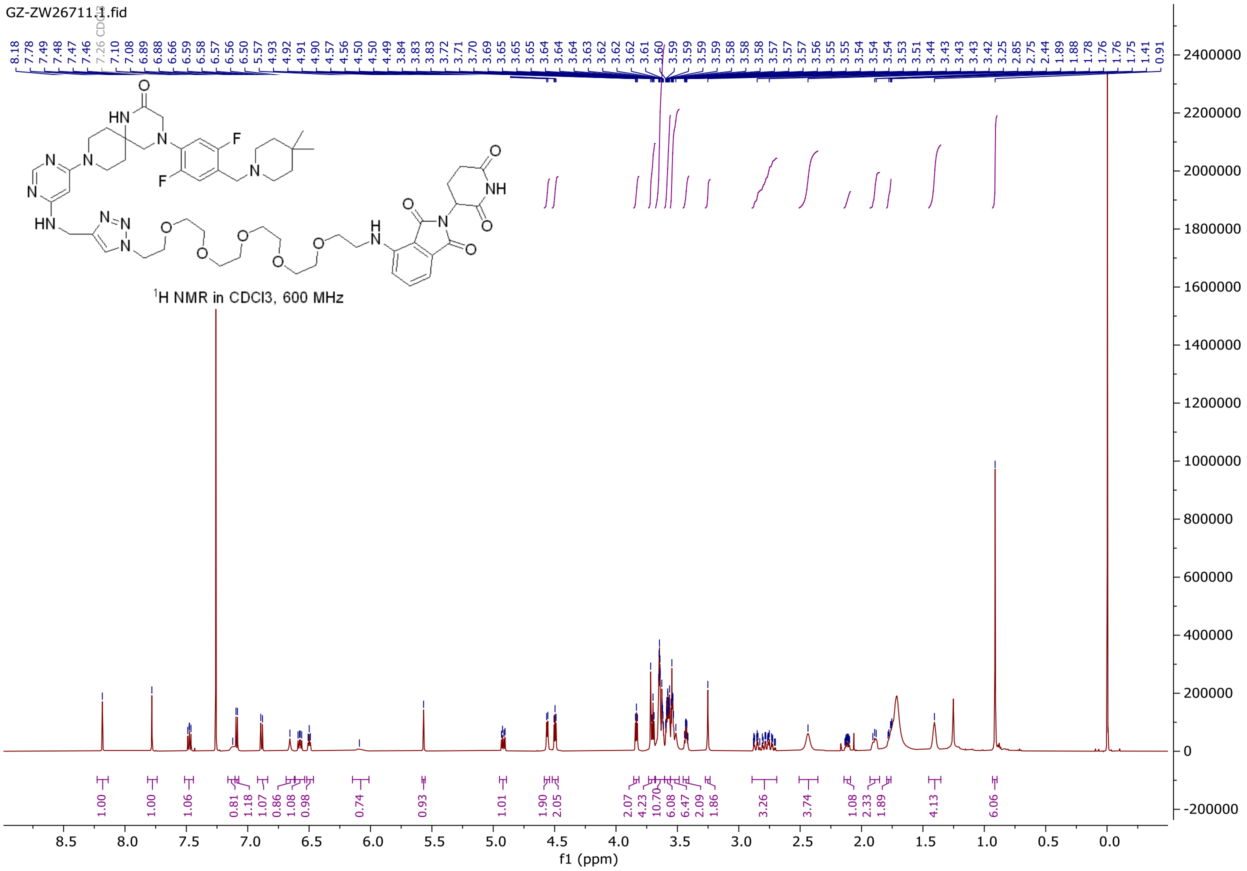

5-((2-(2-(2-(4-(((6-(4-(4-((4,4-dimethylpiperidin-1-yl)methyl)-2,5-difluorophenyl)-2-oxo-1,4,9-triazaspiro[5.5]undecan-9-yl)pyrimidin-4-yl)amino)methyl)-1*H*-1,2,3-triazol-1-yl)ethoxy)ethoxy)ethyl)amino)-2-(2,6-dioxopiperidin-3-yl)isoindoline-1,3-dione (ZW27831)

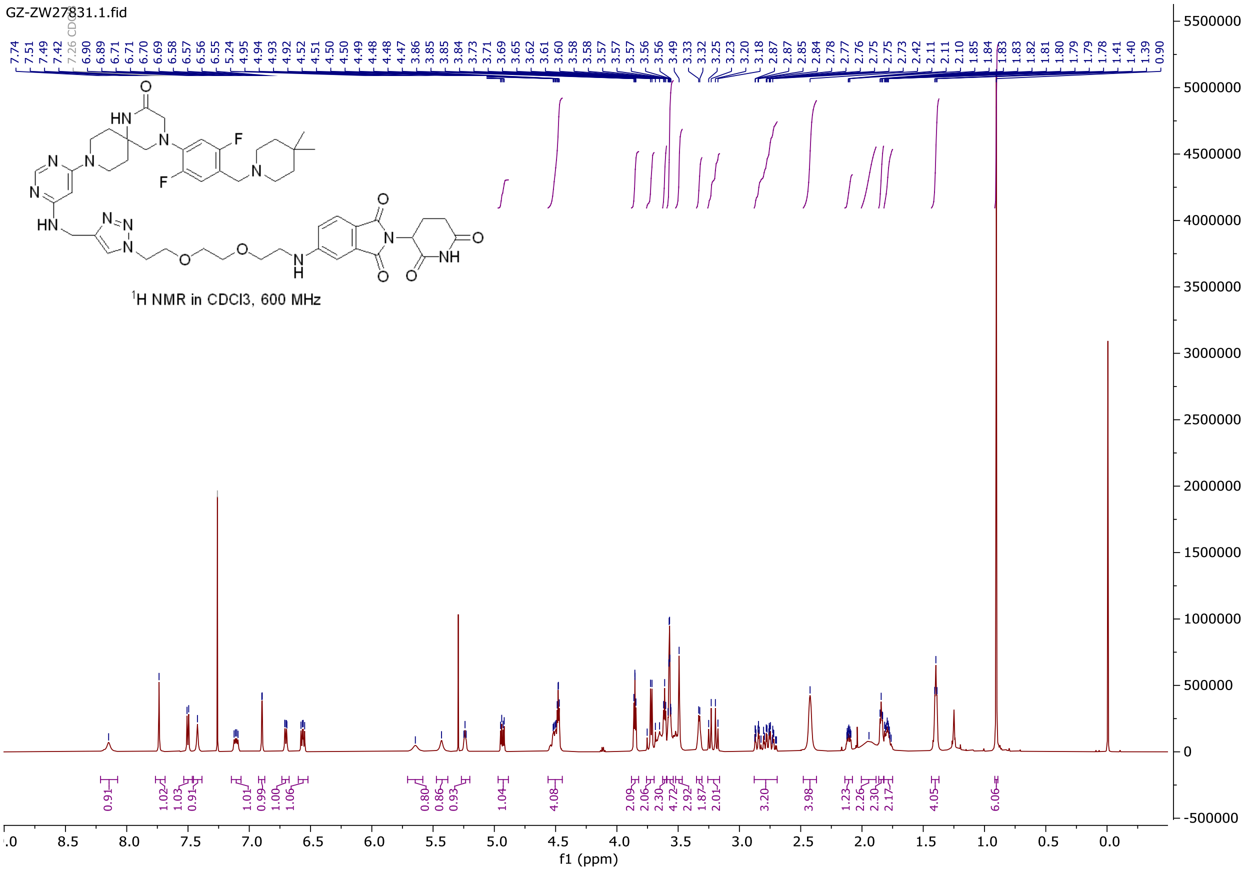

5-((2-(2-(2-(2-(4-(((4-(4-(4-((4,4-dimethylpiperidin-1-yl)methyl)-2,5-difluorophenyl)-2-oxo-1,4,9-triazaspiro[5.5]undecan-9-yl)pyrimidin-2-yl)amino)methyl)-1*H*-1,2,3-triazol-1-yl)ethoxy)ethoxy)ethoxy)ethyl)amino)-2-(2,6-dioxopiperidin-3-yl)isoindoline-1,3-dione (ZW27833)

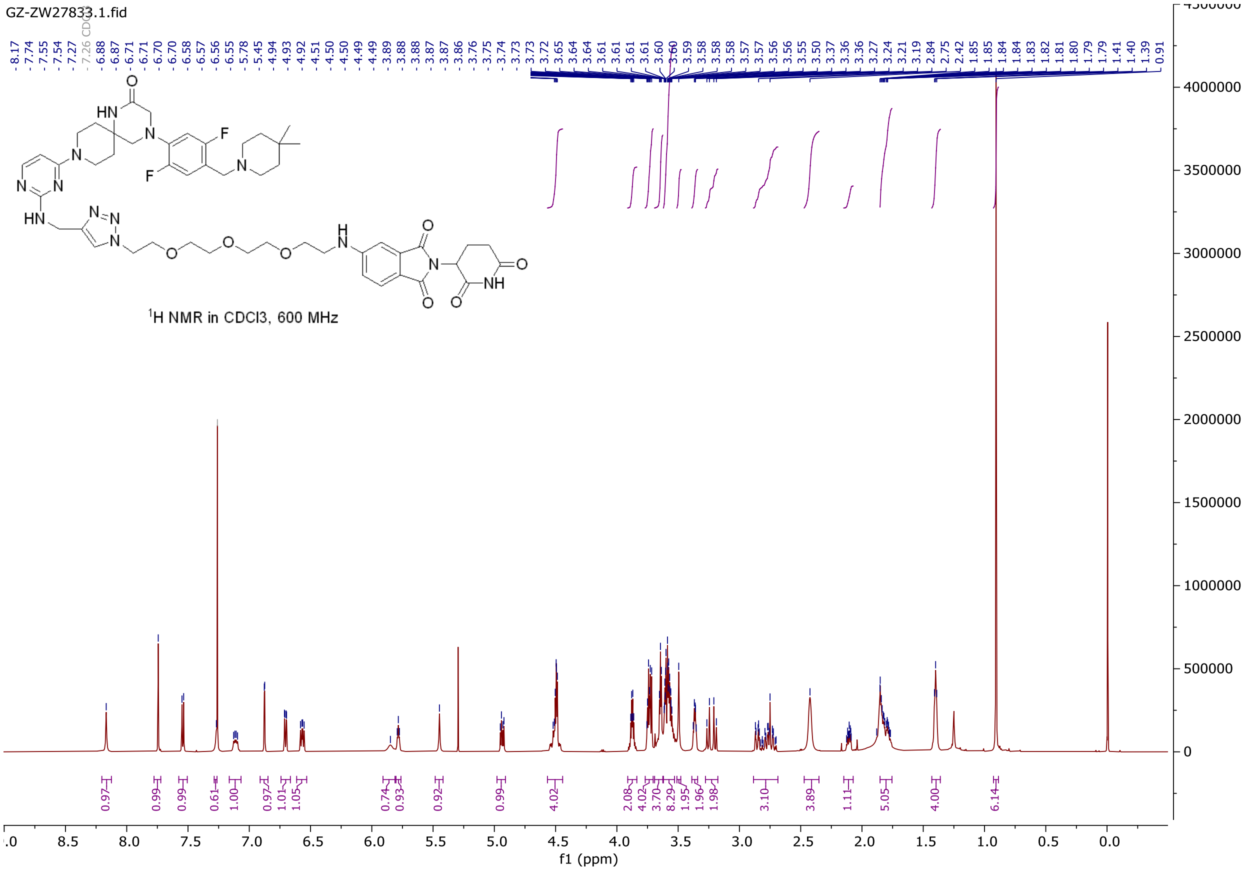

5-((14-(4-(((6-(4-(4-((4,4-dimethylpiperidin-1-yl)methyl)-2,5-difluorophenyl)-2-oxo-1,4,9-triazaspiro[5.5]undecan-9-yl)pyrimidin-4-yl)amino)methyl)-1*H*-1,2,3-triazol-1-yl)-3,6,9,12-tetraoxatetradecyl)amino)-2-(2,6-dioxopiperidin-3-yl)isoindoline-1,3-dione (ZW27828)

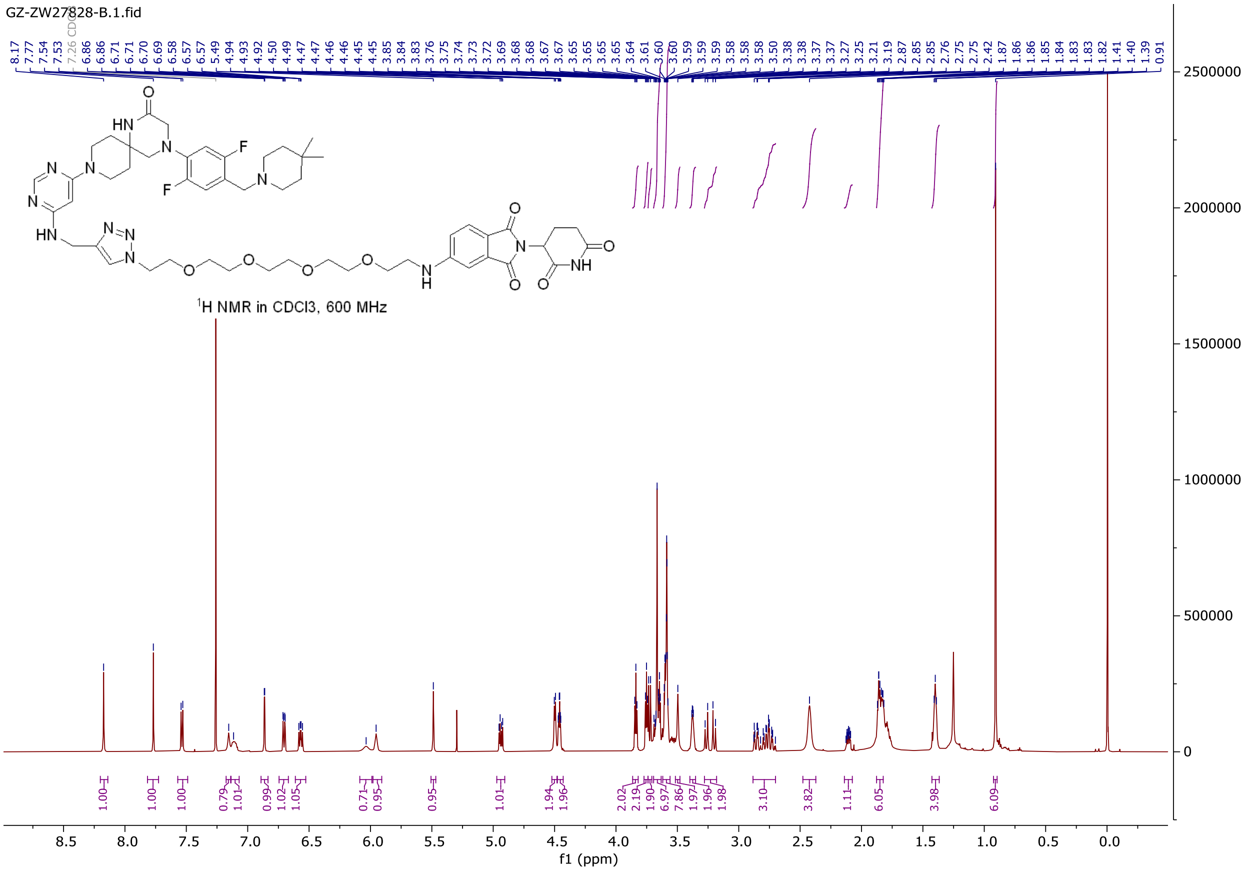

5-((17-(4-(((6-(4-(4-((4,4-dimethylpiperidin-1-yl)methyl)-2,5-difluorophenyl)-2-oxo-1,4,9-triazaspiro[5.5]undecan-9-yl)pyrimidin-4-yl)amino)methyl)-1*H*-1,2,3-triazol-1-yl)-3,6,9,12,15-pentaoxaheptadecyl)amino)-2-(2,6-dioxopiperidin-3-yl)isoindoline-1,3-dione (ZW27841)

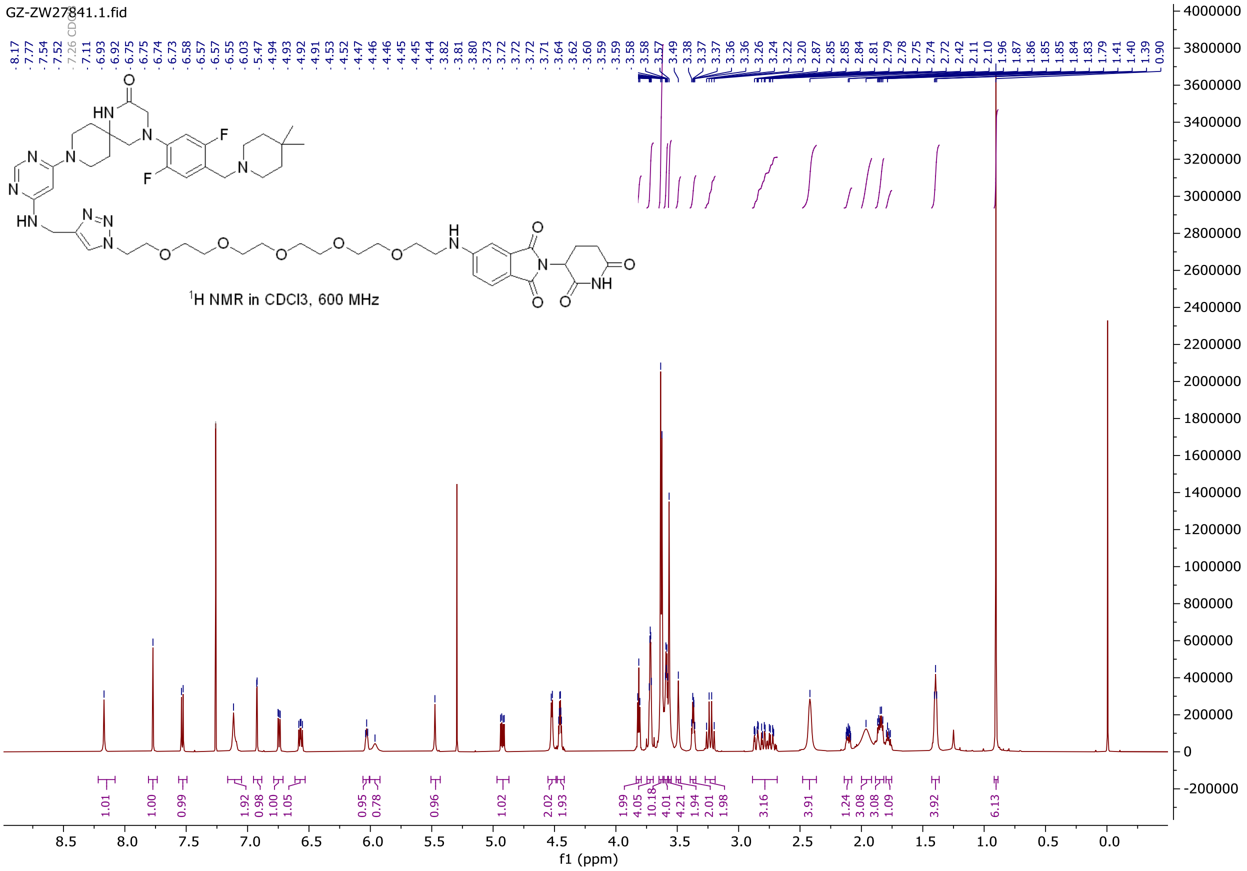

SUPPLEMENTARY FIGURE S1

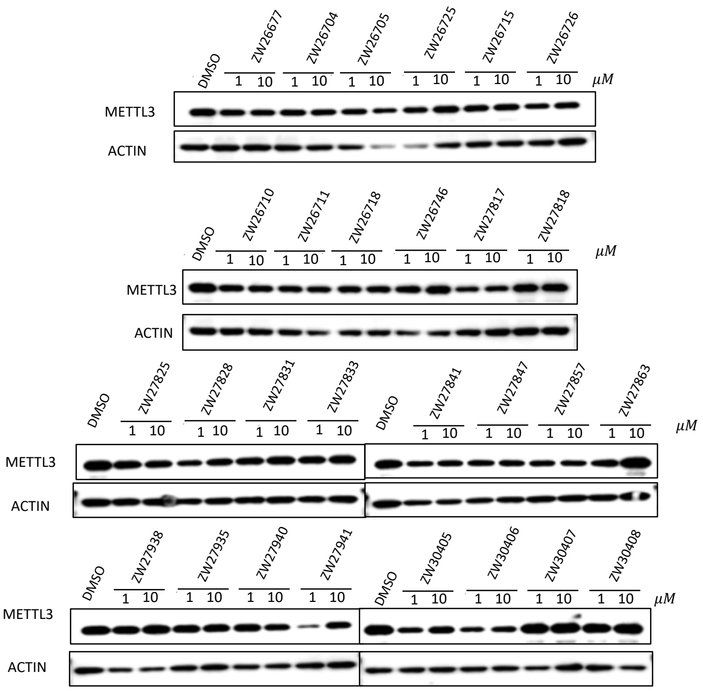

Supplementary Fig. S1. METTL3 degradation induced by the METTL3 PROTACs with different linkers and linker lengths. MOLM13 cells were treated with different concentrations of indicated PROTACs for 24 h. Representative immunoblots are shown and ACTIN was used as an equal loading control in all immunoblot analyses.

SUPPLEMENTARY TABLE S1

	%degradation of METTL3	
**Compound Name**	**1 μM**	**10 μM**
ZW26677	29	34
ZW26704	26	20
ZW26705	0	0
ZW26725	0	0
ZW26715	8	7
ZW26726	28	33
ZW26710	24	21
ZW26711	18	0
ZW26718	1	5
ZW26746	0	0
ZW27817	40	47
ZW27818	19	15
ZW27825	16	36
ZW27828	34	20
ZW27831	18	3
ZW27833	2	11
ZW27841	5	0
ZW27847	0	12
ZW27857	25	31
ZW27863	3	0
ZW27938	0	0
ZW27935	0	16
ZW27940	0	10
**ZW27941**	**76**	**39**
ZW30405	48	38
ZW30406	59	50
ZW30407	0	13
ZW30408	24	0

Supplementary Table S1. METTL3 degradation induced by the METTL3 PROTACs with different linkers and linker lengths.

MOLM13 cells were treated with different concentrations of indicated PROTACs for 24 h. The protein content was normalized against ACTIN levels, and the percentage of METTL3 protein degradation was calculated using ImageJ Software through densitometry quantification.

SUPPLEMENTARY TABLE S2

Gene	Forward Primer	Reverse Primer
**BCL2**	GGTGGGGTCATGTGTGTGG	CGGTTCAGGTACTCAGTCATCC
**c-MYC**	GGCTCCTGGCAAAAGGTCA	CTGCGTAGTTGTGCTGATGT
**GAPDH**	GAGTCAACGGATTTGGTCGT	GACAAGCTTCCCGTTCTCAG
**CDKN1A(p21)**	CGATGGAACTTCGACTTTGTCA	GCACAAGGGTACAAGACAGTG
**MDM2**	GGCAGGGGAGAGTGATACAGA	GAAGCCAATTCTCACGAAGGG
**CDC25B**	CAGGTCTCTGCATGGATTCC	TGCTCGTTTCGAATGATCCG

Supplementary Table S2. Primers used for RT-qPCR

SUPPLEMENTARY TABLE S3

hVHLshRNA#1-5’	CCGGCCCTATTAGATACACTTCTTACTCGAGTAAGAAGT GTATCTAATAGGGTTTTTG
hVHLshRNA#1-3’	AATTCAAAAACCCTATTAGATACACTTCTTACTCGAGTA AGAAGTGTATCTAATAGGG
hVHLshRNA#2-5’	CCGGGATCTGGAAGACCACCCAAATCTCGAGATTTGGGT GGTCTTCCAGATCTTTTTG
hVHLshRNA#2-3’	AATTCAAAAAGATCTGGAAGACCACCCAAATCTCGAGA TTTGGGTGGTCTTCCAGATC
hMETTL3 shRNA#1-5’	CCGGGCAAGTATGTTCACTATGAAACTCGAGTTTCATAG TGAACATACTTGCTTTTTG
hMETTL3 shRNA#1-3’	AATTCAAAAAGCAAGTATGTTCACTATGAAACTCGAGTT TCATAGTGAACATACTTGC

hMETTL3 shRNA#2-5’:	CCGGGCTGCACTTCAGACGAATTATCTCGAGATAATTCG TCTGAAGTGCAGCTTTTTG
hMETTL3 shRNA#2-3’	AATTCAAAAAGCTGCACTTCAGACGAATTATCTCGAGAT AATTCGTCTGAAGTGCAGC

Supplementary Table S3. The Sequence of shRNAs

SUPPLEMENTARY FIGURE S2

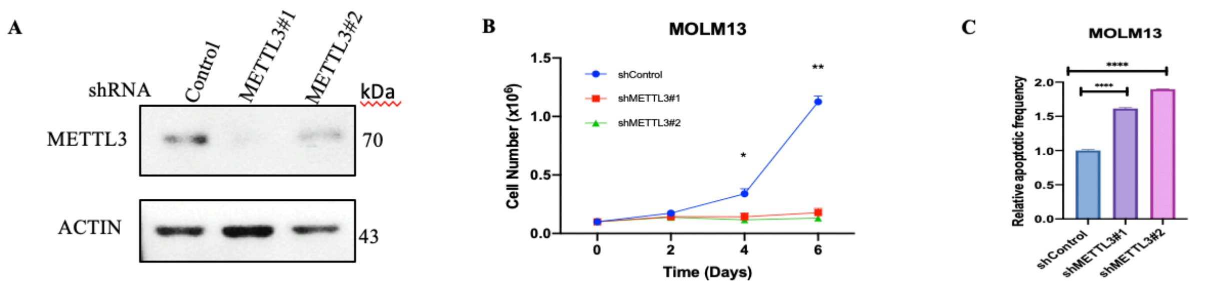

Supplementary Figure S2. METTL3 Knockdown affects the proliferation and survival of human AML cells. A. Western blot analysis of METTL3 expression in MOLM13 cell line expressing PLKO.1 or PLKO.1-METTL3 shRNAs **B.** Viable cell growth of MOLM13 cells expressing PLKO.1 or PLKO.1-METTL3 shRNAs **C.** Flow cytometric analysis of apoptosis of MOLM13 cell lines expressing PLKO.1 or PLKO.1-METTL3 shRNAs.

SUPPLEMENTARY FIGURE S3

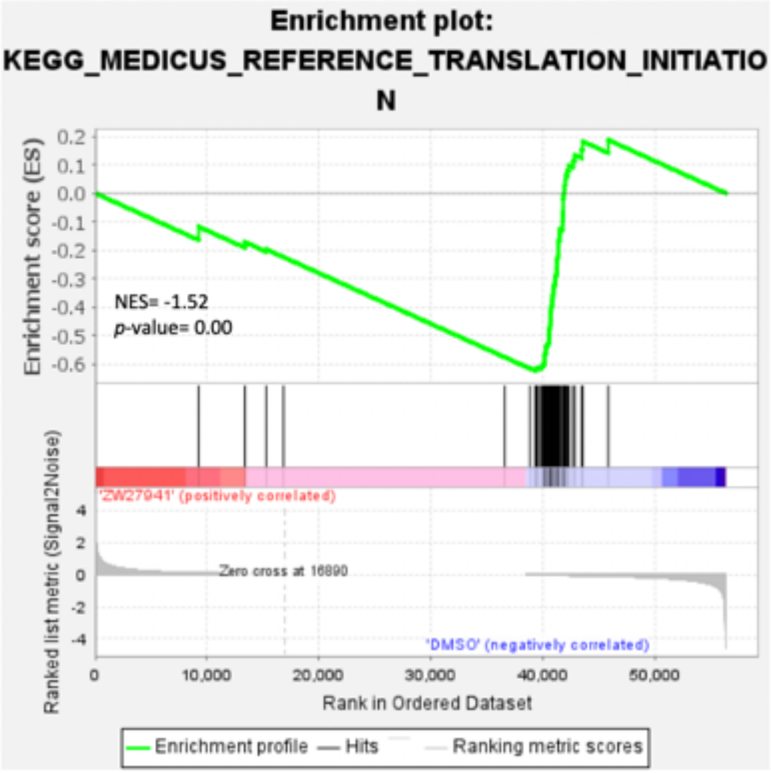

Supplementary Figure S3. GSEA plots showing the enrichment of genes in translation initiation gene set in RNA-Seq following DMSO or ZW27941 treatment in MOLM13 cells.

SUPPLEMENTARY FIGURE S4

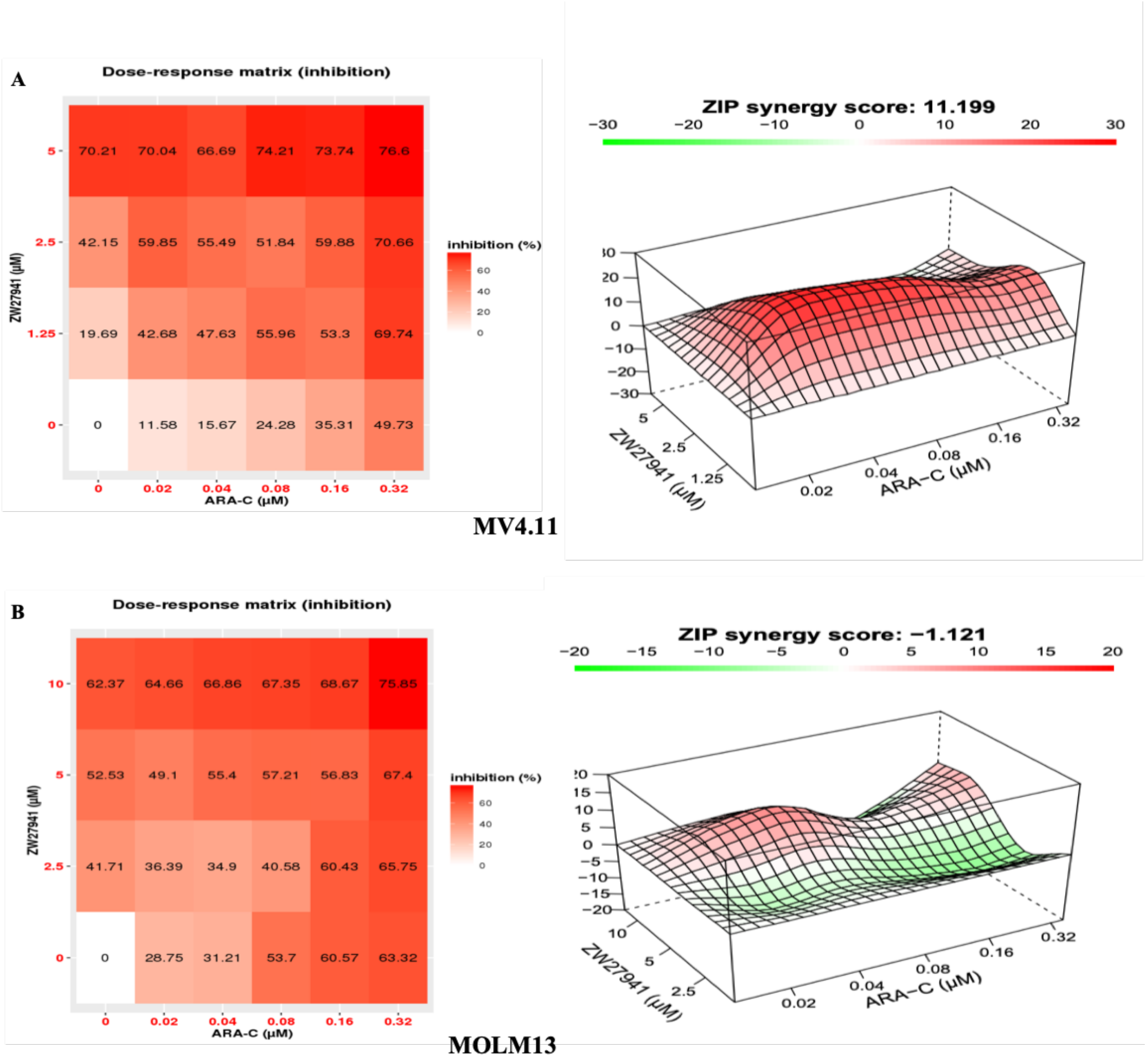

Supplementary Figure S4. ZW27941 enhanced the efficacy of other therapeutic agents used for AML treatment

Drug dose matrix and Synergy landscapes of human MV4.11 (A) and MOLM13 (B) cells treated with increasing concentrations of ZW27941, ARA-C, or their combinations for 72 h. Cell viability was evaluated using the CCK-8 assay. The presented results are representative of three independent experiments. The matrix illustrates the percentage of inhibition of treated cells compared to vehicle controls. Drug combination landscapes were built using the Bioconductor package "synergyfinder."
